# Architecting a Bismacrocycle through a Single C–C Bond Connection

**DOI:** 10.1021/jacsau.6c00910

**Published:** 2026-07-13

**Authors:** Yunlong Li, Haokun Li, Qiyuan Zhou, Mingyu Qu, Pengzhong Chen, Jian Xu, Yuwen Wang, Guangyu Zhu, Zhenpin Lu

**Affiliations:** † Department of Chemistry, 53025 City University of Hong Kong, Kowloon 999077, Hong Kong, P. R. China; ‡ State Key Laboratory of Fine Chemicals, Frontiers Science Center for Smart Materials Oriented Chemical Engineering, School of Chemical Engineering, 12399 Dalian University of Technology, Dalian 116024, P. R. China; § School of Chemistry and Materials Science, Hangzhou Institute for Advanced Study, 638898 University of Chinese Academy of Sciences, Hangzhou 310024, P. R. China; ∥ City University of Hong Kong Shenzhen Research Institute, Shenzhen 518057, P. R. China

**Keywords:** bismacrocycle, C−C bond connection, Scholl reaction, host−guest chemistry, organo-photocatalysts, photocytotoxic anticancer candidate

## Abstract

The covalent linkage of preformed supramolecular entities represents a powerful yet synthetically challenging strategy for constructing sophisticated functional systems, crucial for advances in enzyme mimicry, catalysis, and materials. Addressing the core difficulty of precise, site-selective coupling, we report a direct one-step C–C coupling of carbazole-based macrocycles **2** under Scholl reaction conditions to afford bismacrocycle **1**. Interestingly, when using the smaller ring precursor **4** under similar reaction conditions, we obtain the intermolecular dehydrogenative product **3**. DFT computations provide insight into the distinct formation pathways of monomacrocycle **3** and bismacrocycle **1**. Moreover, we investigated the photophysical properties of compounds **1**–**4**, confirming that all are suitable hosts for C_70_, with their binding constants determined. Notably, bismacrocycle **1** demonstrates exceptional photocatalytic activity for debrominative borylation, outperforming other macrocycles (**2**–**4**) in catalytic efficiency. Finally, we also examined the anticancer activities of these newly synthesized macrocycles. Collectively, this work provides a versatile synthetic strategy for a complex bismacrocycle and establishes its significant dual potential as a selective host, a powerful photocatalyst, and a photocytotoxic anticancer candidate.

## Graphical abstract

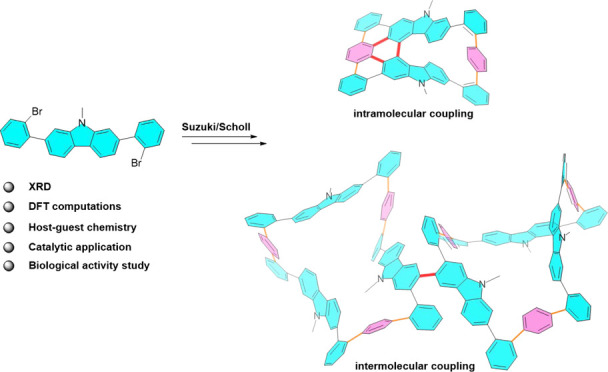

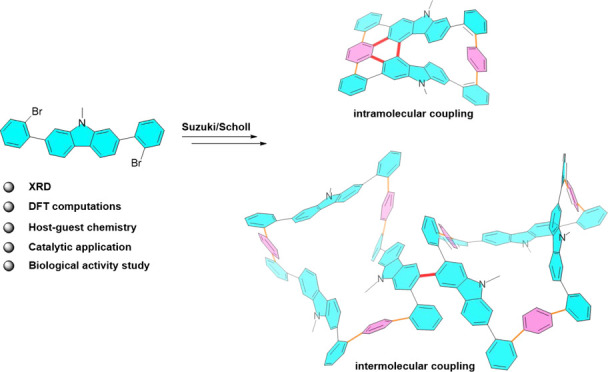

Connecting two or more supramolecular entities is a transformative strategy that enhances the capabilities of individual components, resulting in systems with synergistic and emergent properties.^1^ This integration creates cooperative effects, improved preorganization, and novel functions.^2^ Deliberately linking these building blocks establishes a versatile platform for functional molecular design, enabling advanced applications in catalysis, smart materials, and molecular recognition.^3^ Previously, noncovalent interactions, such as hydrogen bonding and π-stacking, have been employed to synthesize supramolecular polymers.^4^ Although covalent linkages can lead to robust and stable networks, a primary synthetic challenge is the precise connection of two preformed supramolecular entities, which is often complicated by the presence of multiple similar reactive sites.

Supramolecular macrocycles serve as foundational hosts in modern chemistry, with their functional cavities meticulously engineered from scaffolds such as phenol, pyrrole, and glycoluril.^5–8^ By linking two cavities, bis-macrocyclic architectures can be obtained, exhibiting sophisticated cooperative functions, such as enhanced binding affinity, selectivity, and allosteric control reminiscent of proteins. These capabilities position them as powerful candidates for applications in enzyme mimicry,^9^ next-generation therapeutics,^10^ and smart materials,^11^ making the pursuit of efficient synthetic routes a critical goal in the field.

In recent years, a series of bis-macrocycles have been successfully synthesized.^12–19^ The general strategies for their construction typically involve either the simultaneous formation of both rings from acyclic precursors or the linkage of preformed macrocyclic units (see Scheme 1a). However, both approaches generally require careful linker design and multistep syntheses. We therefore propose a more direct and efficient route: constructing the bismacrocyclic core from a single monocyclic compound via a C–C coupling reaction (see Scheme 1b). This strategy is particularly attractive as it eliminates the need for complex precursors and dedicated linker units. The central challenge, however, lies in achieving precise, site-selective coupling to control the final architecture.

**Scheme 1:**
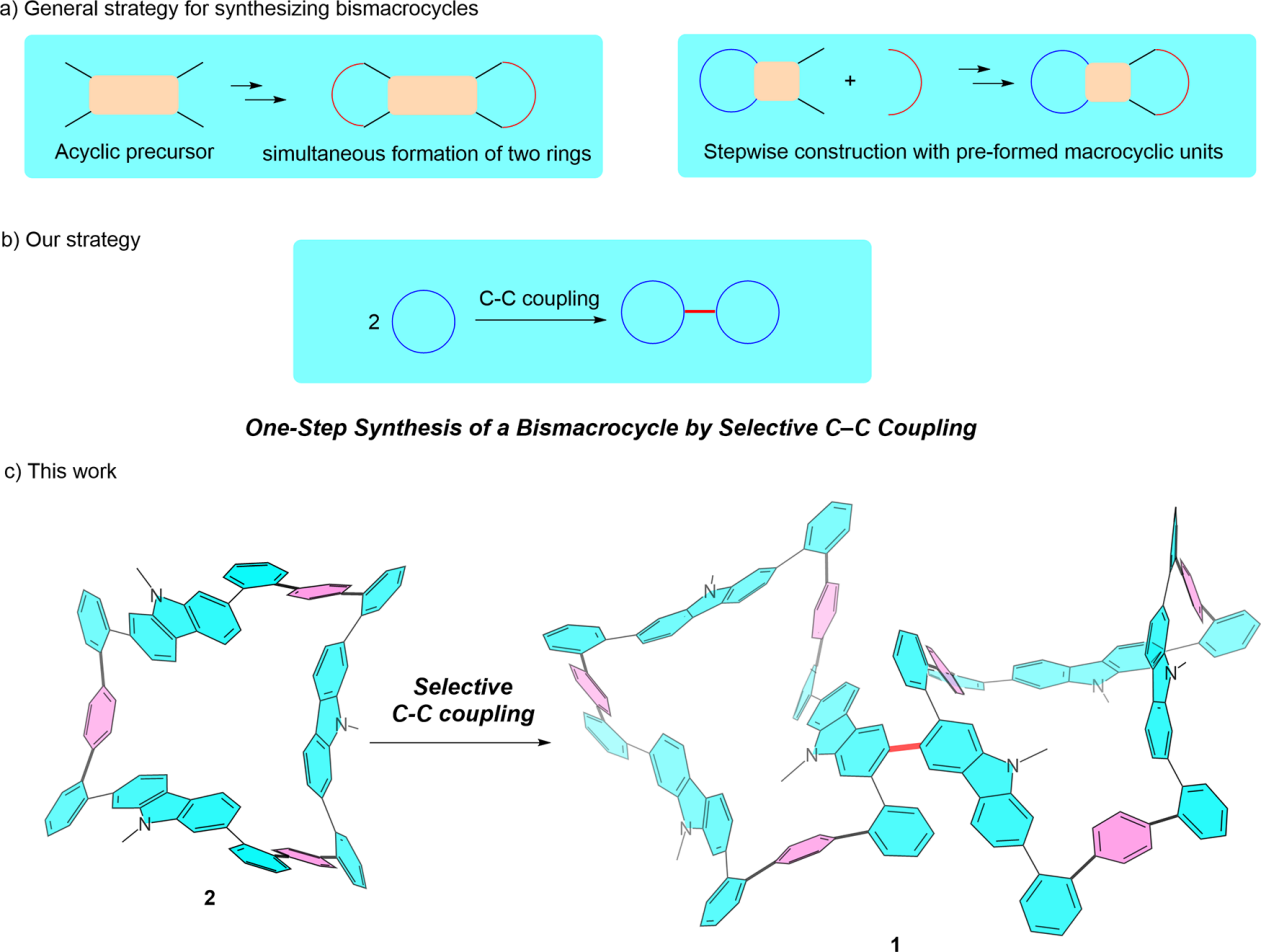
Synthesis of Bismacrocycles

In this context, carbazole-based macrocycles have emerged as highly promising building blocks due to the carbazole unit’s rigid, electron-rich framework, easily modifiable sites, and superior optoelectronic properties.^20^ To explore this, we synthesized two distinct carbazole-based macrocycles (**4** and **2**) via [2 + 2] and [3 + 3] palladium-catalyzed C–C coupling, respectively. Their divergent reactivity under Scholl reaction conditions is particularly notable: macrocycle **2** underwent intermolecular C–C coupling to afford the bismacrocyclic species **1** (Scheme 1c), whereas macrocycle **4** underwent intramolecular dehydrogenative coupling, yielding the monomeric, shovel-shaped scaffold **3**. To elucidate the origins of this selectivity, we probed the reaction mechanisms using DFT computations. Furthermore, we explored the functional potential of the resulting structures, investigating the host–guest chemistry and preliminary applications of macrocycles (**1**–**4**) in catalysis and biological contexts.

## Synthesis and Structure of Bismacrocycle **1**

Initially, carbazole species (**5**) and 1,4-benzenediboronic acid dipinacol ester were selected as subcomponents for constructing new macrocycles (**2** and **4**) through a palladium-catalyzed Suzuki coupling reaction (Scheme 2). After the workup, two macrocyclic products were successfully isolated: compound **4**, generated via a [2 + 2] C–C coupling reaction, was obtained in a 13% yield, while compound **2**, formed through a [3 + 3] C–C coupling reaction, was isolated in a 6% yield. The low yields of these macrocycles are likely attributable to the formation of other cyclized or polymerized species. The structures of macrocycles **4** and **2** were fully characterized by NMR, HRMS, and single-crystal X-ray analyses. The structure of compound **4** appears as a molecular square, where two carbazole units are linked by two phenyl groups (Figure 1a). In contrast, compound **2** consists of three carbazole units and three phenyl groups, forming a rigid, V-shaped scaffold with a distinct molecular cleft (Figure 1b). This structure features a pronounced angular design, with the connections between the carbazole and phenyl groups occurring at the para-positions of the benzene rings. This unique geometry not only resembles a dustpan but also underscores the macrocycle’s potential for versatile interactions.

**Figure 1:**
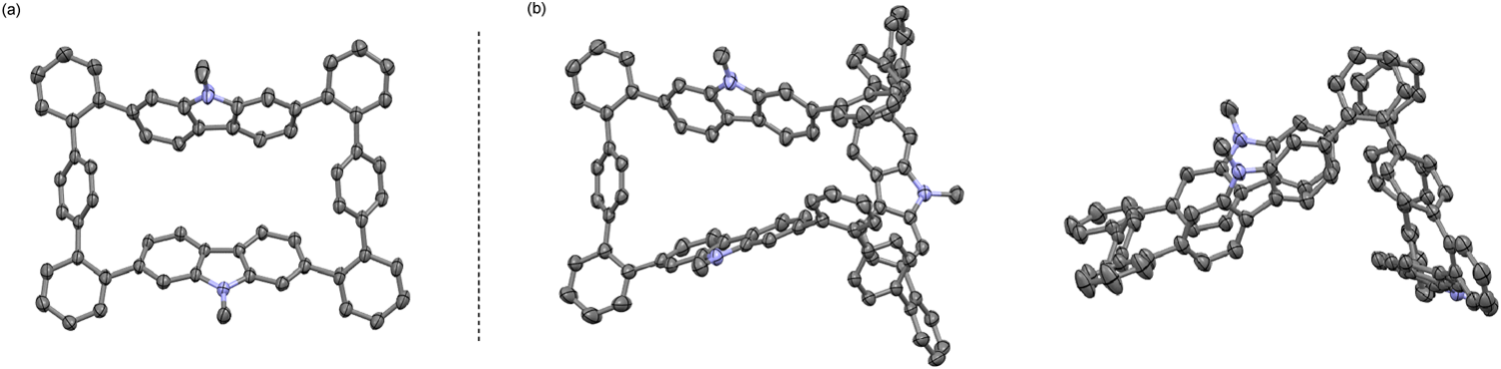
Molecular structures of compounds **4** (a) and **2** (b). Colors: carbon: gray, nitrogen: blue.

**Scheme 2:**
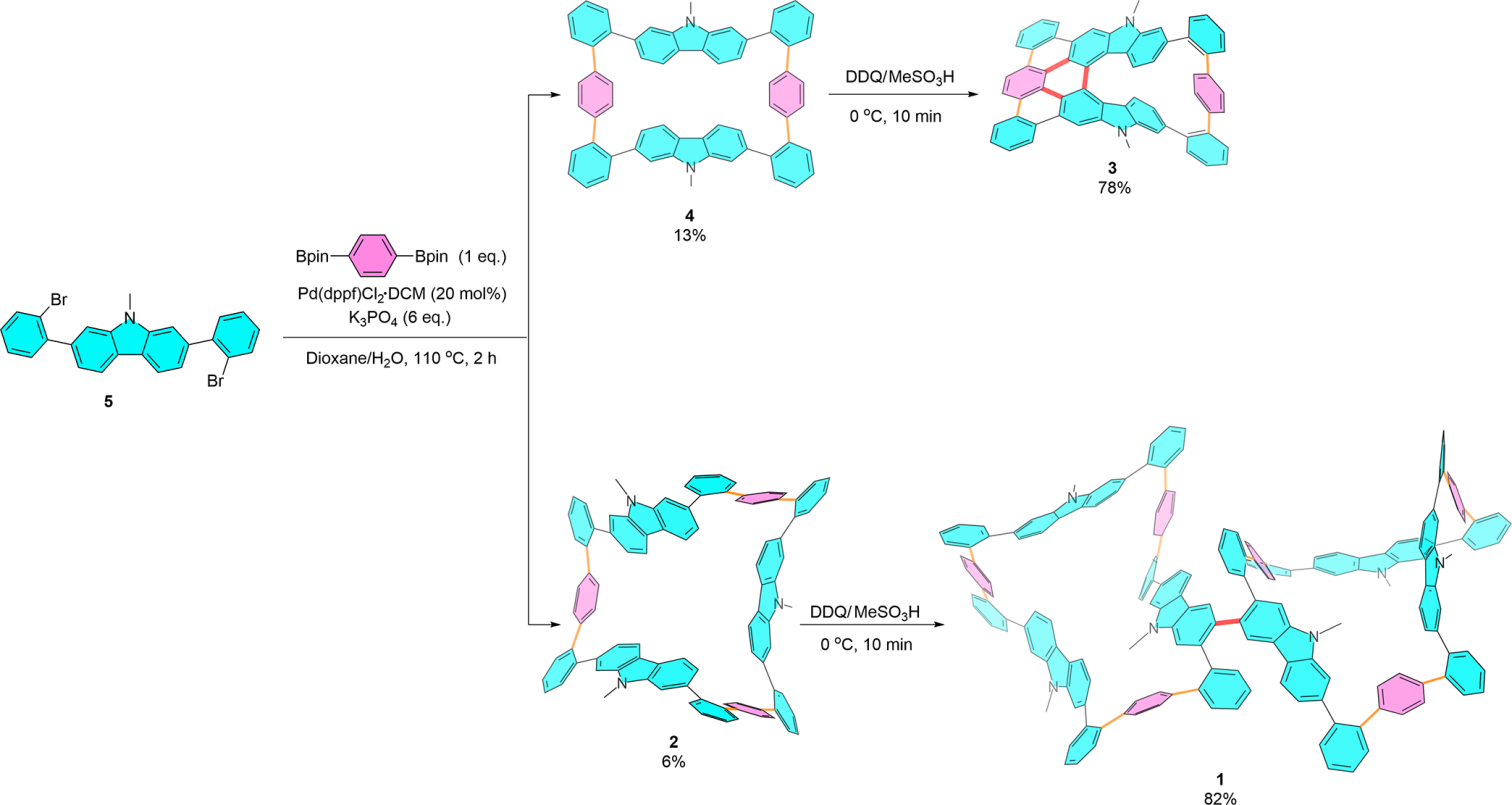
Synthetic Route towards Macrocyclic Compounds **1**, **2**, **3**, **4**

In recent years, Scholl reactions have emerged as a powerful tool for direct C–H couplings of aromatics, enabling the construction of new fused polycyclic aromatics.^21^ We hypothesized that these reactions could facilitate C–C coupling of macrocycles for the synthesis of bismacrocycles. Accordingly, both macrocycles (**2** and **4**) were tested under standard Scholl reaction conditions using DDQ and methanesulfonic acid. In the reaction involving compound **4**, compound **3** was isolated as the major product in a 78% yield. The ^1^H NMR spectrum of compound **3** reveals 12 sets of signals in the aromatic region (See SI, Figure S32), compared to just 5 sets for compound **4** (see SI, Figure S29), which has a highly symmetric structure. The simplified ^1^H and ^13^C NMR spectra of compound **4**, showing a reduced number of resonances, are most reasonably attributed to its intrinsically rigid and highly constrained structural framework, rather than to rapid conformational averaging. Specifically, the covalent linkages formed under the Scholl reaction conditions, together with the extended π-conjugation and steric congestion within the macrocyclic scaffold, significantly restrict internal rotations and conformational flexibility. As a result, the molecule adopts a conformationally locked geometry in solution, giving rise to the observed apparent symmetry. The HRMS spectrum of compound **3** displays a signal at 809.2942 (See SI, Figure S34), indicating the loss of three dihydrogen molecules compared to compound **4**, with no dimerized species observed in the reaction. The structure of compound **3** was further confirmed by single-crystal analyses, consistent with the HRMS results, which showed the formation of three new C–C bonds. Notably, all three C–C couplings occurred on the same side, resulting in the formation of a planar framework with eight fused benzene rings (Figure 2a). Meanwhile, the configuration of the two carbazole units and the connecting phenyl ring was compressed, yielding a curved structure reminiscent of a spoon.

**Figure 2:**
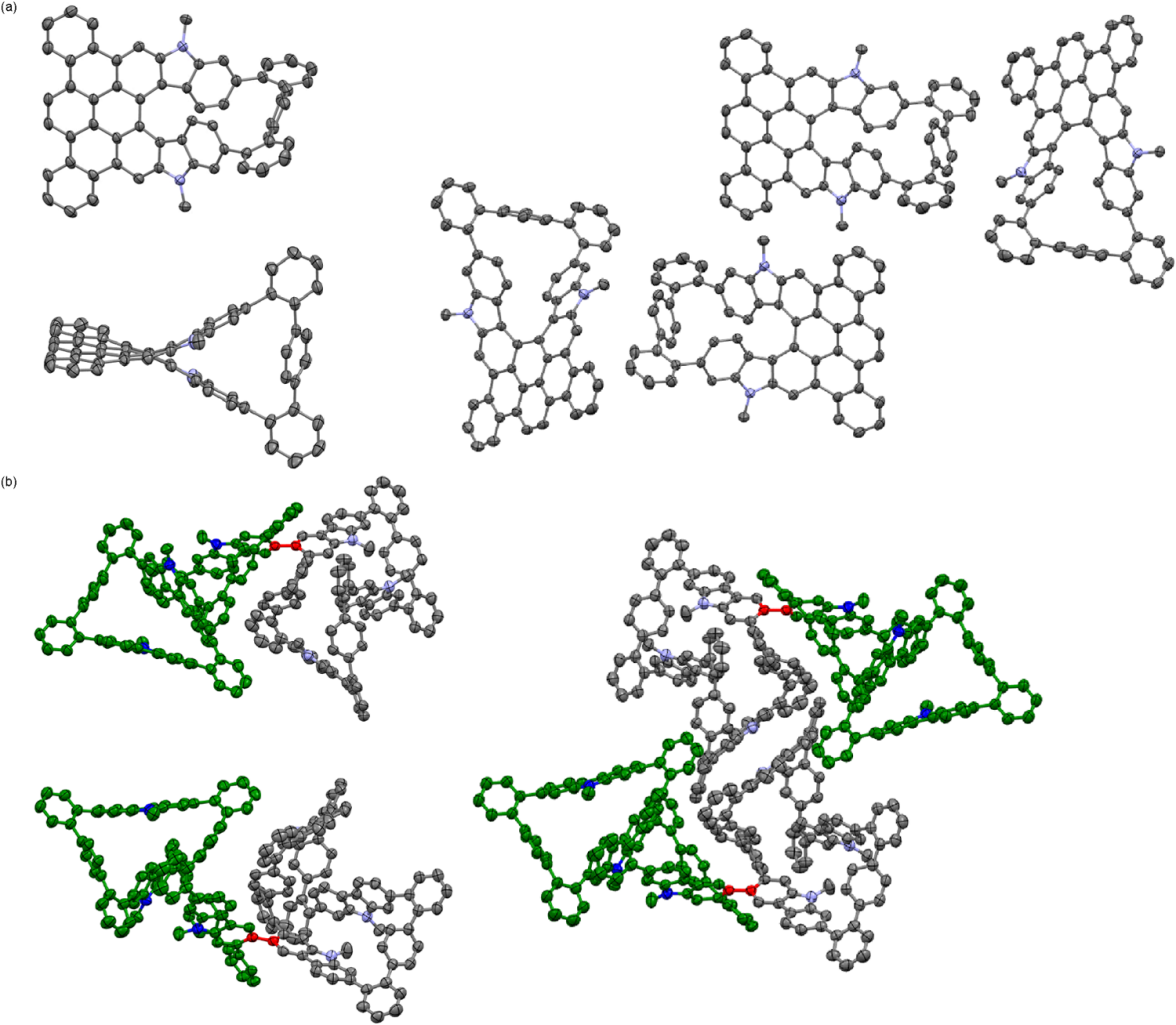
Molecular structures of compounds **3** (a) and **1** (b). Colors: carbon: gray, nitrogen: blue.

Under similar reaction conditions, a new species, compound **1**, was isolated as the major product from the reaction of macrocycle **2**. The ^1^H NMR spectrum of compound **1** reveals a series of broad signals in the aromatic region (see SI, Figure S38). Notably, the HRMS spectrum shows a signal at 2440.9992 (See SI, Figure S40), which is approximately double that of compound **2** (1221.5053), suggesting the formation of a dimeric species. More forcing conditions were also examined, including extending the reaction overnight, increasing the reaction temperature, and using excess DDQ. However, none of these reactions yielded any isolable products (see SI, page 6). Single-crystal X-ray analyses confirmed the formation of the bismacrocycle, indicating that the C–C coupling occurs at the 4-position of the carbazole units, linking two macrocycles (Figure 2b). The formation of the C–C bond has a negligible impact on the structure of one of the macrocyclic rings, which adopts a V-shaped configuration similar to that of compound **2**. However, the geometry of the other ring is significantly distorted, resulting in a more complex folded structure. This structural distortion also accounts for the intricate ^1^H NMR spectrum of compound **1**, as the proton environments in both rings are markedly different. Notably, the bismacrocycle was isolated in an 82% yield, and the reaction was completed in just 10 min. According to DFT computational results, the remarkable regioselectivity of compound **1** is governed by thermodynamics, as the other possible coupling products are substantially less stable (see SI, Figure S6).

## DFT Computations

To understand the distinct reaction pathways involved in the formation of compounds **1** and **3**, we calculated the detailed reaction pathway of **1** using quantum chemistry method.^22^ In this type of Scholl reaction, the formation of the C–C bond can occur via either a radical cation pathway or an arenium ion pathway (Figure 3). In this study, we examined both possibilities for the formation of compound **1**. For the radical cation pathway, the initial one-electron oxidation yields precursor **2-A**. To form the intramolecular C–C coupling product **1**′, an energy barrier of 22.2 kcal/mol must be overcome from **2-A**, via transition state **TS-1A** and intermediate **INT-1A**. In contrast, the generation of the intermolecular C–C coupling product **1** is thermally favored, as it has a significantly lower energy barrier of only 12.9 kcal/mol. Our results for the arenium ion mechanism also support the formation of the dimeric macrocycle product **1**, which is favored with an energy barrier of 8.2 kcal/mol compared to that of the intramolecular C–C coupling product **1**′ (energy barrier of 43.7 kcal/mol). Therefore, in both pathways, our calculations indicate that the major predicted product should be the intermolecular C–C coupling product **1**, as it is both thermodynamically stable and kinetically favorable. This aligns perfectly with our experimental results. Furthermore, we also considered the formation of compound **3**. In this reaction, the intermolecular coupling product **3**′ was not observed, and the reaction energy for compound **3**′ was computed (See SI, Figure S5). The results indicate that the intramolecular product **3** is more stable at −148.5 kcal/mol compared to compound **3**′, which is −43.2 kcal/mol. This demonstrates that compound **3** is also the thermodynamic product. In conclusion, the Scholl reactions in both cases yield only the thermodynamically stable product.

**Figure 3:**
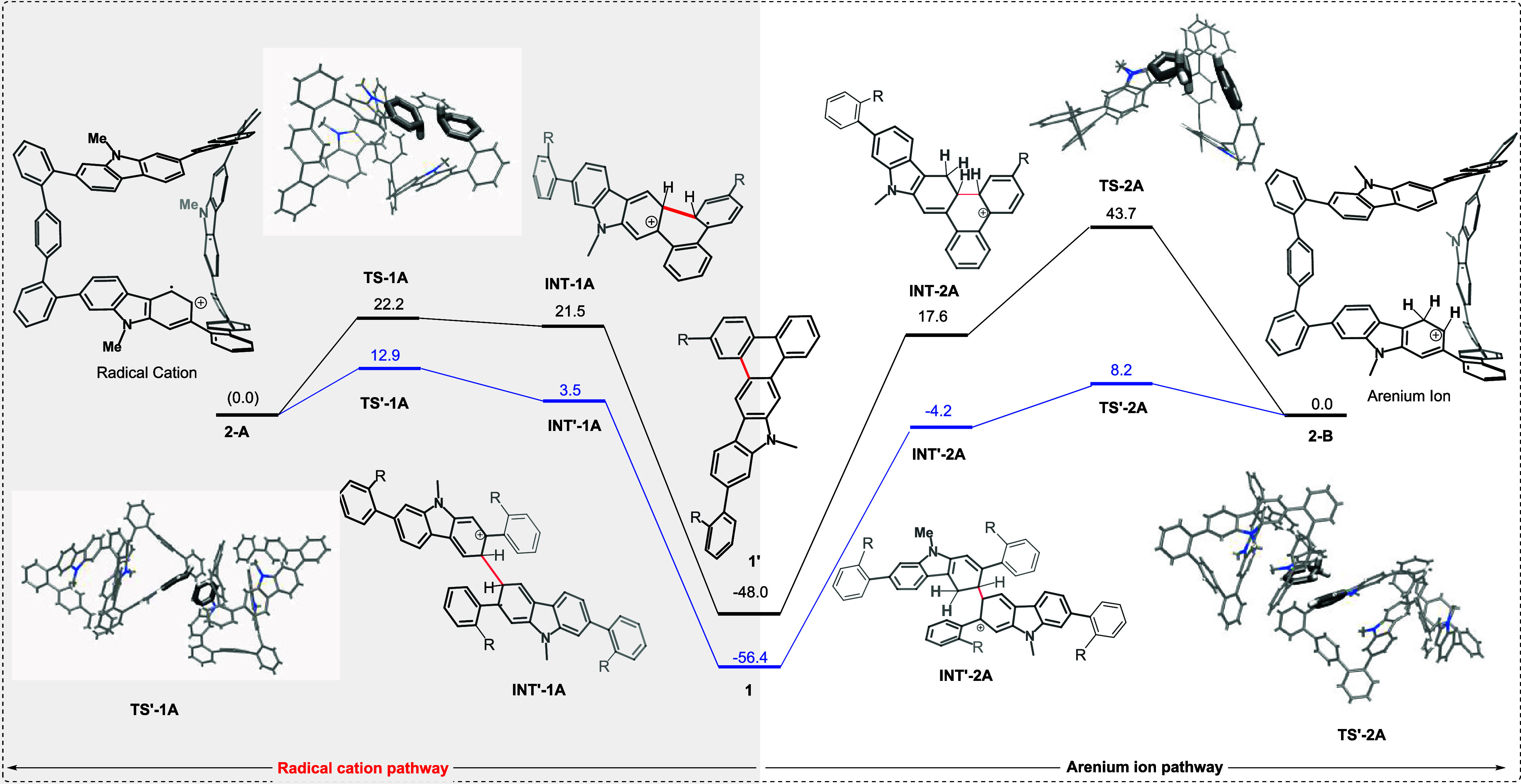
Calculated energy profile for the formation of compounds **1** and **1**′.

## Photophysical Property and Host–Guest Chemistry

The photophysical properties of the macrocyclic compounds (**1–4**) were also investigated (Figure 4). The UV–vis absorption and fluorescence spectra of compounds **1–4** were recorded in dichloromethane as a reference solvent (Figure 4a–d). The absorption spectrum of compound **3** (λ_max_ = 360 nm) is red-shifted by 38 nm compared to compound **4** (λ_max_ = 322 nm), which may be attributed to an extension of the π-conjugated system. In contrast, the absorption wavelengths of compounds **4**, **2**, and **1** are nearly identical (λ_max_ = 321 nm), suggesting the presence of similar chromophores and electron transitions. Furthermore, the emission spectra of compounds **4**, **2**, and **1** exhibit a similar trend, with an emission wavelength (λ_em_) at 401 nm, while compound **3** shows an emission at 478 nm (Figure 4b). Additionally, all four compounds (**1**-**4**) displayed comparable quantum yields, ranging from 0.36 to 0.40 (Figure 4e).^23^ The luminescent lifetimes of compounds **4** and **3**, measured in the liquid state, were found to be 4.06 and 4.80 ns, respectively (Figure 4f,g).

**Figure 4:**
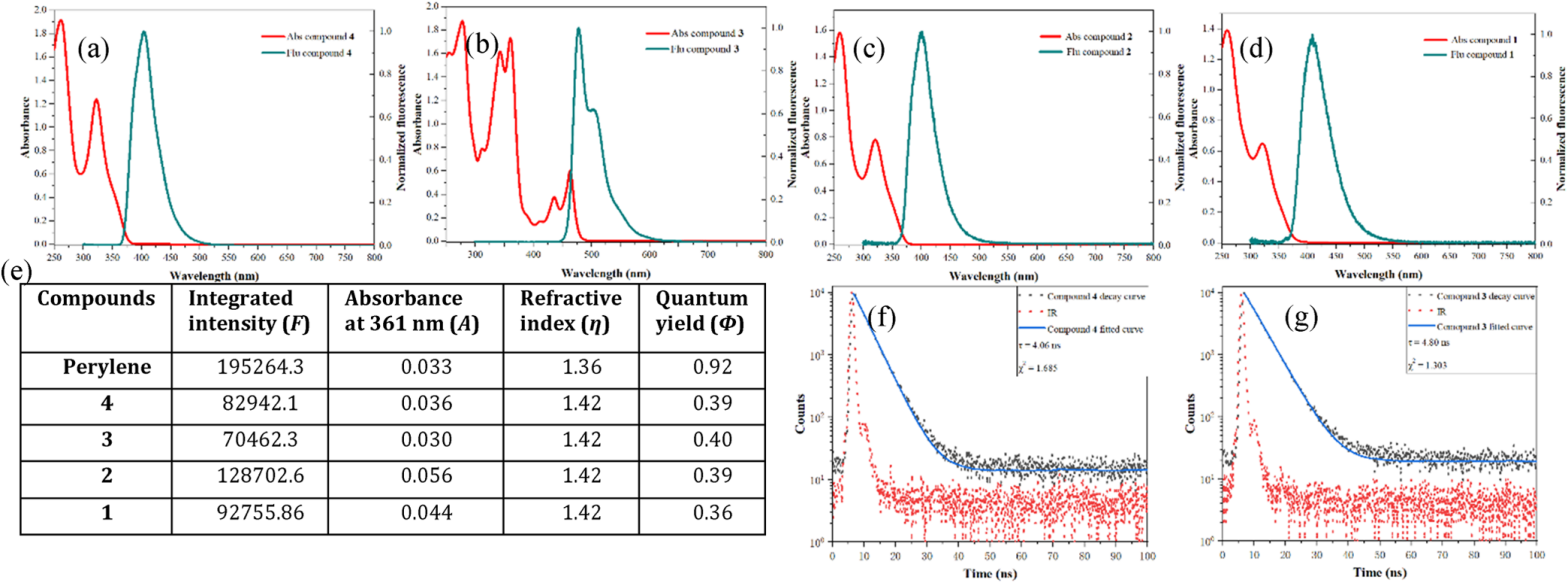
Photophysical properties of compounds (**1**-**4**). (a–d) UV–*vis* absorption (red lines) and emission spectra (green lines) in CH_2_Cl_2_ (2.5 × 10^–5^ M) at room temperature: It showed absorption maxima (λ_abs_) at 262 and 322 nm, and an emission band at 404 nm of **4**. λ_abs_ at 278 and 360 nm, λ_em_ at 478 and 503 nm of **3**. λ_abs_ at 259 and 321 nm, λ_em_ at 401 nm of **2**. λ_abs_ at 259 and 321 nm, λ_em_ at 409 nm of **1**; (e) The fluorescence quantum yields (Φ) were determined by comparing the photoluminescence integrated intensities and absorbance intensities with a standard, perylene (Φ = 0.92); (f, g) Luminescent lifetimes (λ_ex_ = 365 nm) of **4** measured at 404 nm in the liquid state, luminescent lifetimes (λ_ex_ = 373 nm) of **3** measured at 477 nm in the liquid state.

The host–guest chemistry of the newly prepared macrocycles has also been investigated. Initially, we tested the interaction of macrocycle **3** with fullerenes C_60_ and C_70_. While no significant changes were observed in the ^1^H NMR spectrum when macrocycle **3** was mixed with excess C_60_ (2 equiv), a noticeable shift in signals around 7.36 ppm was detected upon the addition of excess C_70_ (see SI, Figure S12). This result indicates that compound **3** exhibits a significant guest–host interaction with C_70_ in the solution. To quantify the C_70_ binding properties of macrocycle **3**, fluorescence titration experiments were conducted in 1,2-dichlorobenzene. Additionally, similar experiments were performed with compounds **1**, **2**, and **4** to assess their interactions with C_70_. The binding constants for the complexes were derived from the fluorescence titration data at 404, 478, 401, and 407 nm (F_0_/F), yielding values of *K*_a_ (C_70_) as follows: 7.0 × 10^4^ M^–1^ for compound **4**, 7.3 × 10^4^ M^–1^ for compound **3**, 5.6 × 10^4^ M^–1^ for compound **2**, and 6.4 × 10^4^ M^–1^ for compound **1** (See SI, Figures S13–S16). Notably, these binding constants indicate a clear preference for macrocycle **3** to bind C_70_ over compounds **4, 2**, and **1**, likely due to external π–π interactions rather than size-matched encapsulation, providing a more rational explanation for the observed selectivity. According to Job’s plot, a 1:1 host–guest binding stoichiometry was established for compounds (**1–4**) with C_**70**_. Interestingly, despite having two rings, compound **1** only interacts with one molecule of C_70_, similar to compound **2**. ^1^H NMR study of the host–guest interaction between compound **1** and C_70_ further supports the formation of a 1:1 host–guest complex (see SI, Figure S12). Computational studies indicate that C_70_ and **1** interact through a partial encapsulation mode, where C_70_ engages cooperatively with both the carbazole and phenyl units (see SI, Figures S7–S9). This behavior may be attributed to the folded structure of the second macrocycle, which hinders further host–guest interactions.

## Catalytic Application

Electron-rich nitrogen-based molecules hold promise as organo-photocatalysts for visible-light-driven synthetic transformations.^24^ We hypothesized that carbazole-based macrocyclic compounds could facilitate reductive dehalogenative borylation. Under irradiation with a 395 nm LED lamp, using compound **3** as the catalyst, pyridine as an additive, and DIPEA and potassium carbonate as bases, we achieved a borylation of aryl halides with a yield of 61% (Table 1, entry 3). Further testing of three additional carbazole-based macrocycles revealed that compound **1** demonstrated the best catalytic performance, yielding 72% (Table 1, entry 1). However, when the light source was switched to 365 nm or omitted entirely, the yield dropped to 37% or resulted in no product formation (Table 1, entries 5–6). We also explored a series of substrates (Scheme 3), finding that both electron-donating and electron-withdrawing groups at the para or meta positions of the aromatic ring were tolerated, leading to the corresponding products: **7a** (4-OMe, 73%), **7b** (4-Me, 52%), **7c** (4-COOEt, 61%), **7d** (4-CN, 53%), and **7e** (3-OMe, 48%). However, the *o*-CN substituted product **7f** was difficult to obtain, likely due to steric hindrance. Additionally, phenylboronic acid esters without other substituents (**7g**) were synthesized with a yield of 50%. When 4-bromo-1,1′:4′,1″-terphenyl was used as a reactant, the borylation product **7h** was obtained in 68% yield. Importantly, when fused-ring aromatic hydrocarbons replaced the simpler aromatic hydrocarbons, the target products were effectively synthesized as well (**7i**: 45% and **7j**: 25%). A plausible mechanism for the borylation of aryl halides is proposed (see SI, Figure S17). In this mechanism, the photoexcited catalyst Cat. 1* is quenched by ArBr through a single-electron transfer (SET) process, resulting in the generation of an aryl radical species. The catalyst is then regenerated to its ground state via reductive quenching mediated by DIPEA, and the borylation of the radical species produces the desired product **7**.

**Scheme 3:**
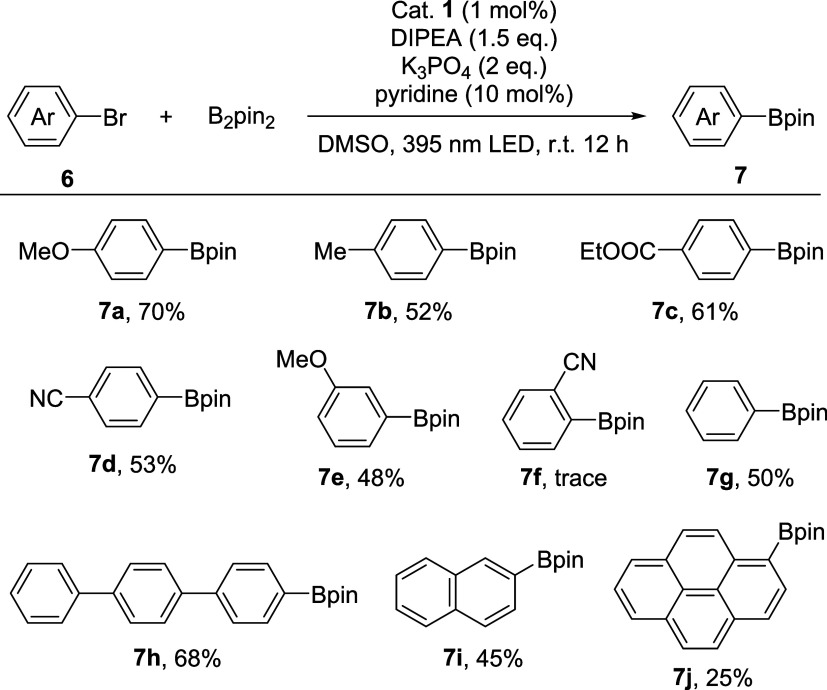
Debrominative Borylation Scope

**Table 1:**
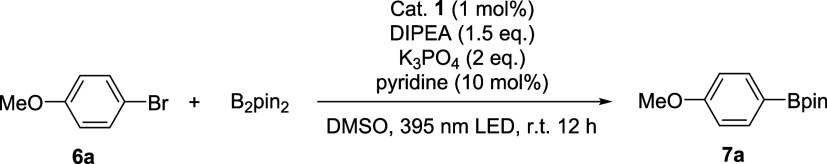
Visible Light-Driven, Biamacrocycle **1** Catalyzed Borylation of Aryl Halides

entry^a^	variation from conditions	yield of **7a** ^b^ (%)
1	none	72
2	Cat. **4** instead of Cat. **1**	9
3	Cat. **3** instead of Cat. **1**	61
4	Cat. **2** instead of Cat. **1**	30
5	365 nm instead of 395 nm	37
6	No light	0

a Conditions: **6a** (0.3 mmol), B_2_Pin_2_ (0.6 mmol), Cat. **1** (0.003 mmol), DIPEA (0.45 mmol), K_3_PO_4_ (0.6 mmol), pyridine (0.03 mmol), DMSO (1 mL), 395 nm LED, r.t., 12 h.

b The yields were determined by ^1^H NMR analysis with CH_2_Br_2_ as the internal standard.

## Biological Activity Study

Finally, we evaluated the biological activity of these newly prepared macrocycles. In the dark, the tested compounds **1**–**4** showed negligible cytotoxicity against A549 and MDA-MB-231 cancer cells and WI-38 normal fibroblasts, whereas cisplatin and doxorubicin were markedly cytotoxic (see SI, Table S7). Under light irradiation, compound **1** reduced the viability of A549 and HeLa cells at its highest tested concentration (50 μM, Figure 5), and compounds **2**–**4** exhibited 72 h half-maximal inhibitory concentration (IC_50_) values ranging from 27.4 to 33.9 μM (see SI, Table S8 and Figure S18). The cell images demonstrate that compound **3** did not accumulate in mitochondria, lysosomes, or plasma membranes (Figure S19) yet effectively killed A549 cells upon irradiation by inducing apoptosis and necrosis (see SI, Figure S20). These death pathways were corroborated by PI/Annexin V double staining, which revealed a marked increase in late apoptotic and necrotic populations after light exposure (see SI, Figures S21 and S22). Overall, among the tested macrocycles, compound **3** emerges as the most promising photocytotoxic anticancer candidate.

**Figure 5:**
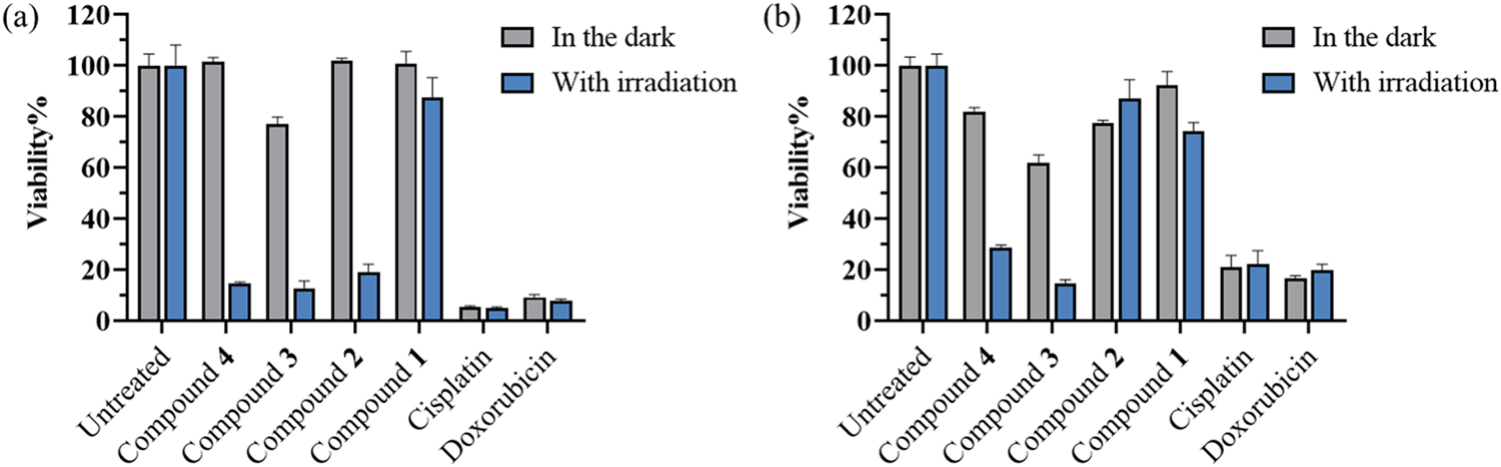
Cell viability of (a) A549 and (b) HeLa cells after treatment with compounds **1** to **4** (50 μM), cisplatin (50 μM), or doxorubicin (20 μM) for 6 h, followed by 1 h white-light (400–700 nm, 7.6 mW/cm^2^) irradiation, and evaluated at 72 h total treatment time.

In conclusion, we have synthesized bismacrocycle **1** through a one-step C–C coupling of carbazole-based macrocycle **2** under standard Scholl coupling conditions. In contrast, the smaller ring compound **4** yielded an intermolecular dehydrogenative product **3**. DFT computational results indicate that both compounds **1** and **3** are the more thermodynamically stable products, with the divergent formation of these two species likely attributed to differences in their strain forces. Furthermore, all the newly prepared macrocycles are confirmed to be suitable hosts for the fullerene C_70_. Despite the presence of two rings in compound **1**, only one C_70_ molecule interacts with the host, likely due to the folded geometry of the second ring, which restricts further host–guest interactions. Compound **1** also shows promising potential as an organophotocatalyst for debrominative borylation reactions, exhibiting catalytic efficiency that surpasses that of other macrocycles (**2**–**4**). Finally, our preliminary biological activity studies indicate that these macrocycles exhibit noteworthy photocytotoxicity toward cancer cells, with compound **3** emerging as the most promising anticancer drug candidate among them.

## Supplementary Material



## Notes

Supplementary figures, detailed experimental procedures, and characterization data for all new compounds are included in the supporting information (PDF)
