# Supplementary material for: Architecting a Bismacrocycle through a Single C–C Bond Connection
Source: JACS Au. 2026 Jul 13;6(7):4344–52. doi: 10.1021/jacsau.6c00910 (PMC13417300; doi:10.1021/jacsau.6c00910)
Supplement: Supplementary file 1 [file au6c00910_si_001.pdf]

Supporting Information

Architecting a Bismacrocycle Through a Single C–C Bond Connection

Yunlong Li,[1‡] Haokun Li,[1‡] Qiyuan Zhou,[1,4‡] Mingyu Qu,[1] Pengzhong Chen,[2] Jian Xu,[3] Yuwen Wang,[3] Guangyu Zhu,[1,4*] and Zhenpin Lu[1*]

zhenpilu@cityu.edu.hk , guangzhu@cityu.edu.hk

[1] Department of Chemistry, City University of Hong Kong, 999077 Kowloon, Hong Kong, P. R. China
[2] State Key Laboratory of Fine Chemicals, Frontiers Science Center for Smart Materials Oriented Chemical Engineering, School of Chemical Engineering, Dalian University of Technology, Dalian 116024, China.
[3] School of Chemistry and Materials Science, Hangzhou Institute for Advanced Study, University of Chinese Academy of Sciences, Hangzhou 310024, China
[4] City University of Hong Kong Shenzhen Research Institute, Shenzhen 518057, P. R. China.

Table of Contents

1. General Information S2

2. Experiment Procedure and Characterization Data S3

2.1 Synthetic route towards macrocyclic compounds S3

2.2 X-Ray crystallographic studies for **4**, **3**, **2** and **1** S12

2.3 DFT computation S20

2.4 Photophysical properties of the macrocyclic compounds **4**, **3**, **2** and **1** S24

2.5 The host-guest interactions between **3** and **1** with C_{70} S27

2.6 Fluorescent titration and Job's plot experiments S28

2.7 Visible light-driven, biamacrocycle **1** catalyzed borylation of aryl halides S32

2.8 Biological activity study S36

3. Copies of Spectrums S41

4. References S61

1. General Information

Reactions were carried out in oven-dried or flame-dried glassware under a nitrogen or argon atmosphere, unless otherwise noted. Solvents used in the workup, extraction, and column chromatography were obtained from commercial suppliers and used without further purification. Reactions were magnetically stirred and monitored by thin-layer chromatography (TLC, 0.25 mm) on pre-coated silica gel plates from Merck. Flash chromatography was performed using silica gel 60 (particle size 0.040–0.062 mm) supplied by Grace. ^{1}H and ^{13}C NMR spectra were recorded on Bruker AV-300, 400, and 600 spectrometers. Chemical shifts are reported in parts per million (ppm) relative to internal standards: chloroform (7.26 ppm for ^{1}H and 77.16 ppm for ^{13}C), dichloromethane (5.32 ppm for ^{1}H and 53.84 ppm for ^{13}C), dimethyl sulfoxide (2.50 ppm for ^{1}H and 39.5 ppm for ^{13}C), or benzene (7.16 ppm for ^{1}H and 128.06 ppm for ^{13}C). Abbreviations for signal coupling are as follows: s (singlet), d (doublet), t (triplet), q (quartet), and m (multiplet). High-resolution mass spectra were recorded using a Bruker autoflex maX MALDI-TOF/TOF. UV-vis spectra were obtained with a UV-Visible Scanning Spectrophotometer (Shimadzu 1700). All other substrates were commercially available and used as received.

2. Experiment Procedure and Characterization Data

2.1 Synthetic route towards macrocyclic compounds

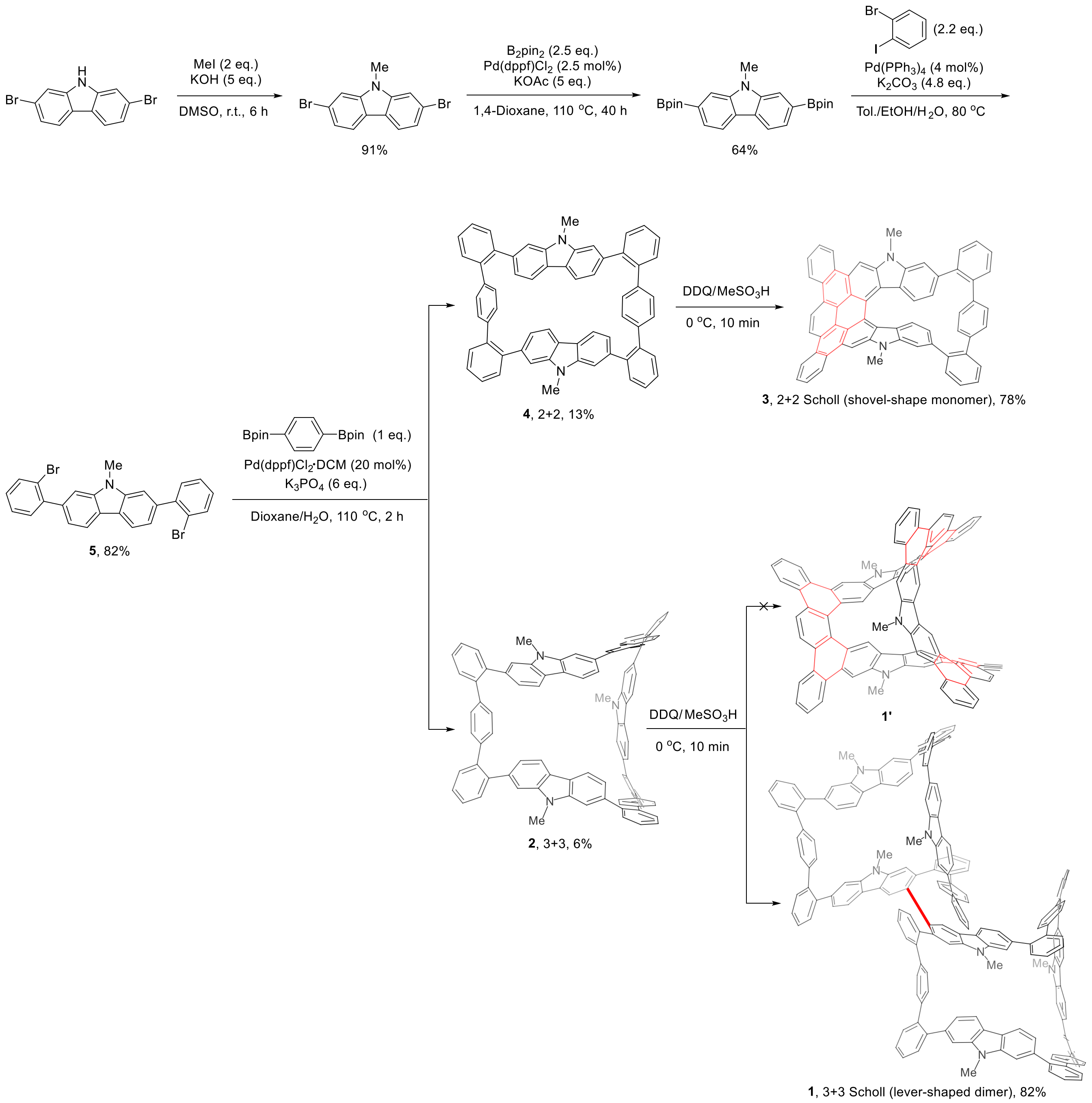

1, 3+3 Scholl (lever-shaped dimer), 82%

2.1.1 General procedure for the synthesis of 2,7-dibromo-9-methyl-9*H*-carbazole

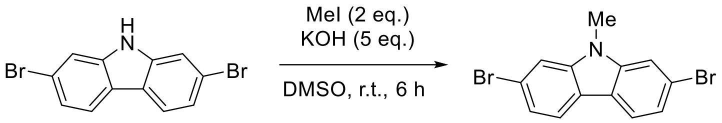

A mixture of 2,7-dibromo-9*H*-carbazole (4.875 g, 15.0 mmol), MeI (4.258 g, 30.0 mmol), and KOH (4.203 g, 75.0 mmol) in 41 mL of DMSO was stirred at 27 °C for 6 hours. The reaction mixture was poured into water (100 mL) and extracted with DCM (200 mL). The organic layer was separated and dried over anhydrous sodium sulfate, all volatiles were evaporated under reduced pressure. The resulting mixture was purified by column chromatography on silica gel (eluent: hexane/dichloromethane = 5:1, v/v), yielding the compound as a white solid (4626 mg, 91%).

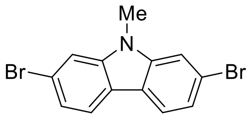

^{1}H NMR (400 MHz, $CDCl_3$) δ = 7.86 (d, J = 8.2 Hz, 2H), 7.51 (d, J = 1.7 Hz, 2H), 7.35 – 7.32 (m, 2H), 3.74 (s, 3H) ppm;

^{13}C NMR (101 MHz, $CDCl_3$) δ = 141.99, 122.73, 121.54, 121.33, 119.86, 111.99, 29.41 ppm.

HRMS (EI) *m/z* calcd for $C_{13}H_{10}Br_2N$ $[M+H]^+$: 337.9180; Found: 337.9187.

2.1.2 General procedure for the synthesis of 9-methyl-2,7-bis(4,4,5,5-tetramethyl-1,3,2-dioxaborolan-2-yl)-9*H*-carbazole

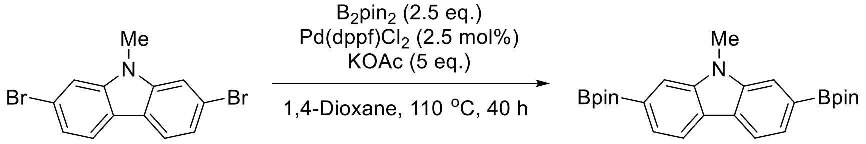

A mixture of 2,7-dibromo-9-methyl-9*H*-carbazole (3.390 g, 10.0 mmol), B_2Pin_2 (6.349 g, 25.0 mmol), Pd(dppf)Cl_2 (182 mg, 0.25 mmol), and KOAc (4.907 g, 50.0 mmol) in 40 mL of 1,4-dioxane was stirred at 110 °C for 40 hours. The reaction mixture was poured into water (100 mL) and extracted with DCM (200 mL). The organic layer was separated and dried over anhydrous sodium sulfate, and all volatiles were then evaporated under reduced pressure. The resulting mixture was purified by column chromatography on silica gel (eluent: hexane/dichloromethane = 3:1, v/v), yielding the compound as a white solid (2.721 g, 64%).

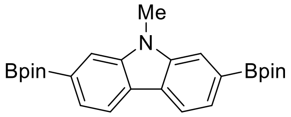

^{1}H NMR (400 MHz, $CDCl_3$) δ = 8.13 (d, *J* = 7.8 Hz, 2H), 7.92 (s, 2H), 7.70 (d, *J* = 7.7 Hz, 2H), 3.94 (s, 3H), 1.41 (s, 24H) ppm;

^{13}C NMR (101 MHz, $CDCl_3$) δ = 141.13, 125.16, 125.04, 120.16, 115.27, 83.97, 77.36, 29.35, 25.08 ppm.

HRMS (EI) *m/z* calcd for $C_{25}H_{34}B_2NO_4$ $[M+H]^+$: 434.2674; Found: 434.2696.

2.1.3 General procedure for the synthesis of 2,7-bis(2-bromophenyl)-9-methyl-9*H*-carbazole (5)

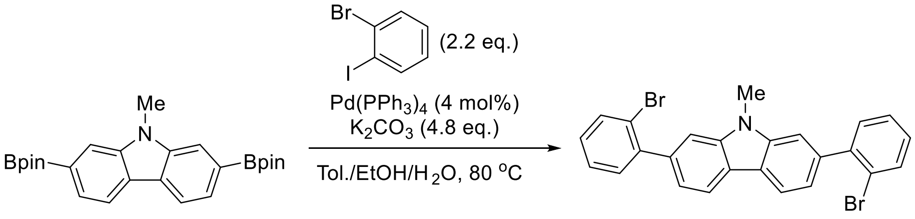

5, 82%

A mixture of 9-methyl-2,7-bis(4,4,5,5-tetramethyl-1,3,2-dioxaborolan-2-yl)-9*H*-carbazole (0.897 g, 2.11 mmol), 1-bromo-2-iodobenzene (1.313 g, 4.64 mmol), $Pd(PPh_3)_4$ (97.1 mg, 0.084 mmol), and K_2CO_3 (1.396 g, 10.1 mmol) in 30 mL of toluene/EtOH/H_2O (2:1:1, v/v/v) was stirred at 80 °C for 24 hours. The reaction mixture was poured into water (30 mL) and extracted with DCM (60 mL). The organic layer was separated and dried over anhydrous sodium sulfate, and all volatiles were then evaporated under reduced pressure. The resulting mixture was purified by column chromatography on silica gel (eluent: hexane/dichloromethane = 8:1, v/v), yielding compound **5** as a white solid (850 mg, 82%).

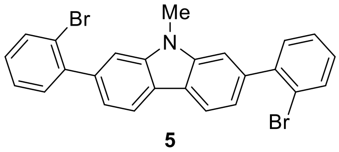

5

^{1}H NMR (400 MHz, $CDCl_3$) δ = 8.19 (d, J = 8.0 Hz, 2H), 7.77 – 7.74 (m, 2H), 7.54 – 7.46 (m, 4H), 7.44 – 7.40 (m, 2H), 7.35 – 7.33 (m, 2H), 7.29 – 7.24 (m, 2H), 3.90 (s, 3H) ppm.

^{13}C NMR (101 MHz, $CDCl_3$) δ = 143.56, 141.28, 139.02, 133.22, 131.78, 128.73, 127.42, 123.13, 122.03, 120.91, 119.96, 109.69, 29.31 ppm.

HRMS (EI) *m/z* calcd for $C_{25}H_{18}Br_2N$ $[M+H]^+$: 489.9806; Found: 489.9854.

2.1.4 General procedure for the synthesis of 2+2 coupling product (4) and 3+3 coupling product (2)

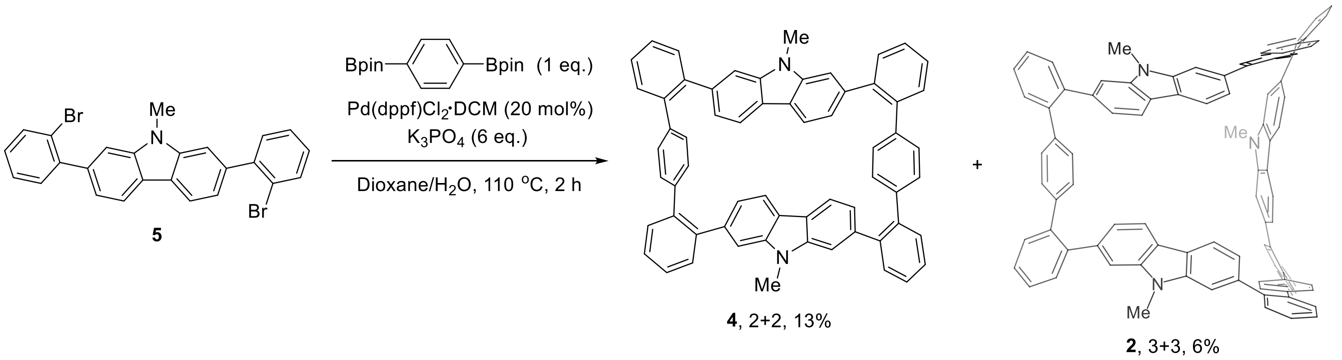

A mixture of 2,7-bis(2-bromophenyl)-9-methyl-9*H*-carbazole (**5**) (491 mg, 1 mmol), 1,4-bis(4,4,5,5-tetramethyl-1,3,2-dioxaborolan-2-yl)benzene (330 mg, 1 mmol), Pd(dppf)Cl_2·DCM (163 mg, 0.2 mmol), and K_3PO_4 (1.274 g, 6 mmol) in 23 mL of 1,4-dioxane/H_2O (5:1, v/v) was stirred at 110 °C for 2 hours. The reaction mixture was poured into water (23 mL) and extracted with DCM (46 mL). The organic layer was separated and dried over anhydrous sodium sulfate, and all volatiles were then evaporated under reduced pressure. The resulting mixture was purified by column chromatography on silica gel (eluent: hexane/ethyl acetate = 9:1, v/v), yielding compound **4** as a white solid (53 mg, 13%) and **2** as a white solid (24.5 mg, 6%).

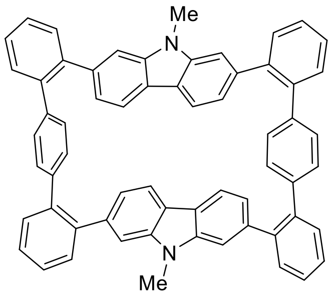

4

^{1}**H NMR** (400 MHz, $CDCl_3$) δ = 7.58 – 7.54 (m, 10H), 7.53 – 7.51 (m, 6H), 7.49 – 7.47 (m, 6H), 7.47 – 7.45 (m, 10H), 6.89 – 6.89 (m, 4H), 3.88 (s, 6H) ppm.

^{13}**C NMR** (101 MHz, $CDCl_3$) δ = 141.82, 140.91, 140.44, 140.25, 139.05, 131.67, 131.42, 129.77, 127.78, 127.42, 122.36, 121.31, 118.94, 109.44, 29.15 ppm.

MALDI-TOF Mass ([$C_{62}H_{43}N_2$]$^+$): simulated: 815.3426, test: 815.3417.

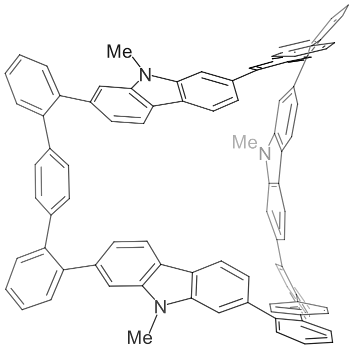

2

Screened reaction conditions and yields:

Condition a: Pd(dppf)Cl_2·DCM (20 mol%), K_3PO_4 (6 eq.), 1,4-dioxane/H_2O (5:1, v/v), 110 °C, 2 hours, 6% yield;

Condition b: $Pd(PPh_3)_4$ (20 mol%), K_3PO_4 (6 eq.), 1,4-dioxane/H_2O (5:1, v/v), 110 °C, 2 hours, 3% yield;

Condition c: $Pd_2(dba)_3$ (20 mol%), K_3PO_4 (6 eq.), 1,4-dioxane/H_2O (5:1, v/v), 110 °C, 2 hours, 0% yield;

Condition d: $Pd(OAc)_2$ (20 mol%), PPh_3 (40 mol%), K_3PO_4 (6 eq.), 1,4-dioxane/H_2O (5:1, v/v), 110 °C, 2 hours, 0% yield;

Condition e: Pd(dtbpf)Cl_2 (20 mol%), K_3PO_4 (6 eq.), 1,4-dioxane/H_2O (5:1, v/v), 110 °C, 2 hours, 1% yield;

Condition f: Pd(dppf)Cl_2·DCM (20 mol%), ^{t}BuONa (6 eq.), 1,4-dioxane/H_2O (5:1, v/v), 110 °C, 2 hours, 4% yield;

Condition g: Pd(dppf)Cl_2·DCM (20 mol%), K_3PO_4 (6 eq.), toluene/H_2O (5:1, v/v), 110 °C, 2 hours, 4% yield;

Condition h: Pd(dppf)Cl_2·DCM (20 mol%), K_3PO_4 (6 eq.), THF/H_2O (5:1, v/v), 110 °C, 2 hours, 0% yield;

Condition i: Pd(dppf)Cl_2·DCM (20 mol%), K_3PO_4 (6 eq.), m-xylene/H_2O (5:1, v/v), 110 °C, 2 hours, 1% yield;

Condition j: Pd(dppf)Cl_2·DCM (20 mol%), K_3PO_4 (6 eq.), MeCN/H_2O (5:1, v/v), 110 °C, 2 hours, 0% yield;

Condition k: Pd(dppf)Cl_2·DCM (20 mol%), K_3PO_4 (6 eq.), DMF/H_2O (5:1, v/v), 110 °C, 2 hours, 0% yield;

Condition l: Pd(dppf)Cl_2·DCM (20 mol%), K_3PO_4 (6 eq.), 1,4-dioxane/H_2O (5:1, v/v), 100 °C, 2 hours, 5% yield.

^{1}H NMR (400 MHz, $CDCl_3$) δ = 7.56 – 7.51 (m, 12H), 7.51 – 7.43 (m, 12H), 7.43 – 7.36 (m, 6H), 7.12 (s, 12H), 7.08 (s, 6H), 6.94 – 6.90 (m, 6H), 3.51 (s, 9H) ppm.

^{13}C NMR (101 MHz, $CDCl_3$) δ = 141.42, 141.07, 140.33, 140.01, 139.34, 131.30, 130.80, 129.51, 127.33, 127.25, 121.18, 121.05, 119.63, 109.85, 28.51 ppm.

MALDI-TOF Mass ($[C_{93}H_{63}N_3]^+$): simulated: 1221.5022, test: 1221.5053.

2.1.5 General procedure for the synthesis of 2+2 Scholl product (3)

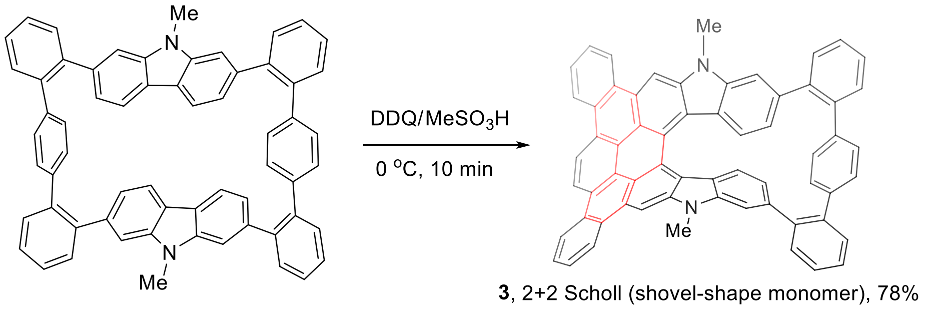

A mixture of compound (**4**) (30 mg, 0.0368 mmol) and DDQ (61.8 mg, 0.272 mmol) in 662 μL (0.0102 mmol) of $MeSO_3H$ and 60 mL of DCM was stirred at 0 °C for 10 minutes under a N_2 atmosphere. The reaction mixture was quenched with 5 mL of MeOH, and poured into water (20 mL), and extracted with DCM (40 mL). The organic layer was separated and dried over anhydrous sodium sulfate, and all volatiles were then evaporated under reduced pressure. The resulting mixture was purified by column chromatography on silica gel (eluent: hexane/dichloromethane = 5:1, v/v), yielding compound **3** as a yellow solid (23.2 mg, 78%).

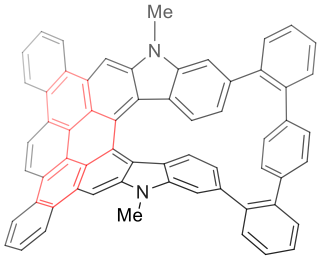

3

^{1}**H NMR** (400 MHz, $CDCl_3$) δ = 9.11 (s, 2H), 9.05 – 9.01 (m, 2H), 8.99 (s, 2H), 8.94 – 8.91 (m, 2H), 7.85 – 7.78 (m, 4H), 7.46 – 7.40 (m, 4H), 7.38 – 7.33 (m, 4H), 7.32 – 7.29 (m, 2H), 7.21 – 7.18 (m, 2H), 7.01 – 6.97 (m, 2H), 6.79 (d, J = 8.3 Hz, 2H), 6.24 – 6.21 (m, 2H), 4.22 (s, 6H) ppm.

^{13}**C NMR** (151 MHz, $CDCl_3$) δ = 143.46, 142.28, 141.79, 141.14, 140.40, 139.98, 130.77, 130.57, 129.85, 129.00, 128.94, 128.91, 128.71, 127.37, 127.11, 126.82, 125.97, 124.25, 124.14, 123.85, 123.58, 121.99, 121.95, 121.87, 121.17, 121.04, 120.34, 108.58, 102.45, 29.86 ppm.

MALDI-TOF Mass ($[C_{62}H_{37}N_2]^+$): simulated: 809.2957, test: 809.2942.

2.1.6 General procedure for the synthesis of 3+3 Scholl product (1)

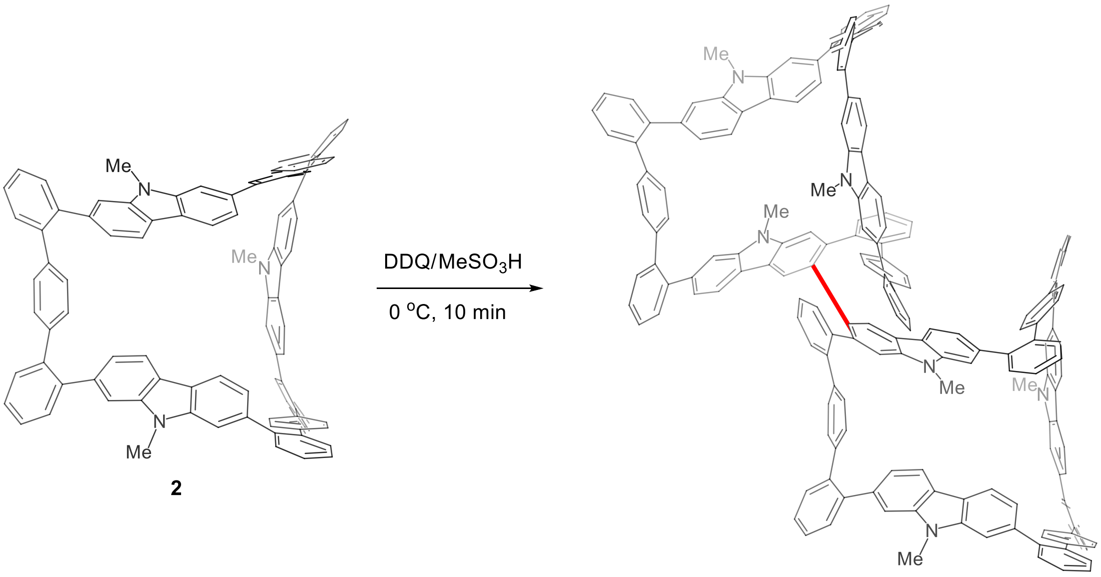

1, 3+3 Scholl (lever-shaped dimer), 82%

A mixture of compound (**2**) (50 mg, 0.0409 mmol) and DDQ (37.2 mg, 0.164 mmol) in 398 μL (0.0061 mmol) of $MeSO_3H$ and 50 mL of DCM was stirred at 0 °C for 10 minutes under a N_2 atmosphere. The reaction mixture was quenched with 3 mL of MeOH, and poured into water (25 mL), and extracted with DCM (50 mL). The organic layer was separated and dried over anhydrous sodium sulfate, and all volatiles were then evaporated under reduced pressure. The resulting mixture was purified by column chromatography on silica gel (eluent: hexane/dichloromethane = 5:1, v/v), yielding compound **1** as a white solid (40.9 mg, 82%).

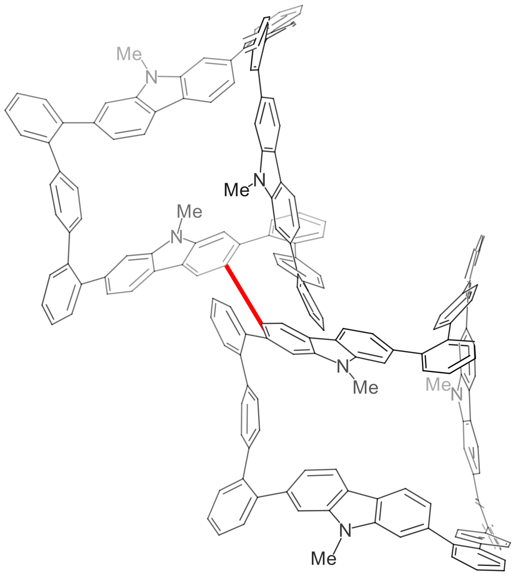

1

^{1}H NMR (400 MHz, $CDCl_3$) δ = 7.54 – 7.45 (m, 28H), 7.44 – 7.35 (m, 28H), 7.34 – 7.29 (m, 6H), 7.19 – 7.09 (m, 14H), 7.04 – 6.04 (m, 12H), 6.92 – 6.89 (m, 4H), 6.81 (s, 4H), 6.69 – 6.62 (m, 6H), 6.06 (s, 4H), 3.48 (s, 6H), 3.13 (s, 6H), 3.00 (s, 6H) ppm.

^{13}C NMR (151 MHz, $CDCl_3$) δ = 141.79, 141.77, 141.49, 141.48, 141.06, 140.96, 140.85, 140.84, 140.60, 140.59, 140.48, 140.31, 140.30, 140.23, 140.21, 139.64, 139.63, 139.36, 139.35, 138.74, 138.73, 132.55, 131.88, 131.47, 131.45, 131.26, 130.90, 130.89, 130.69, 130.67, 130.07, 130.06, 130.04, 130.02, 129.60, 129.58, 129.48, 129.46, 129.36, 129.34, 128.62, 128.61, 127.33, 127.30, 127.05, 127.04, 125.84, 125.83, 124.62, 121.60, 121.59, 121.08, 121.06, 121.04, 121.03, 121.01, 121.00, 120.71, 120.69, 120.27, 120.12, 119.78, 119.77, 119.43, 110.69, 109.84, 108.74, 27.36, 25.67, 22.84 ppm.

MALDI-TOF Mass ($[C_{186}H_{124}N_6]^+$): simulated: 2440.9887, test: 2440.9992.

2.2 X-Ray crystallographic studies for 4, 3, 2 and 1

Single-crystal X-ray diffraction studies for compounds **4, 3, 2** and **1** were carried out on a SMART APEX diffractometer with graphite-monochromated Mo Kα radiation. Cell parameters were obtained by global refinement of the positions of all collected reflections. Intensities were corrected for Lorentz and polarization effects and empirical absorption. The structures were solved by direct methods and refined by full-matrix least squares on F2. All non-hydrogen atoms were refined anisotropically. All hydrogen atoms were placed in calculated positions. Structure solution and refinement were performed using the SHELXL-97 package. The X-ray crystallographic files, in CIF format, are available from the Cambridge Crystallographic Data Centre on quoting the deposition number CCDC 2500172, 2500173, 2500174, and 2500175 for compounds **4, 3, 2** and **1**.

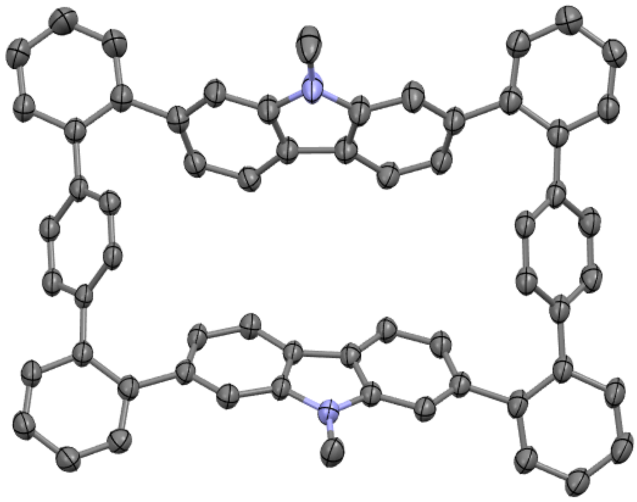

Figure S1. Molecular structure of compound **4**.

Table S1. Crystal data and structure refinement for **4**

Identification code	cu_250312TLF_LYL_122_2_0m_a, 2500172
Empirical formula	$C_{125}H_{86}Cl_2N_4$ (2($C_{62}H_{42}N_2$), CCl_2)
Formula weight	1714.87
Temperature	190 K
Wavelength	1.54184 Å
Crystal system, space group	Triclinic, P-1
Unit cell dimensions	a = 11.9046(4) Å α = 108.034(2)°
	b = 11.9170(4) Å β = 95.747(2)°
	c = 17.3010(6) Å γ = 94.713(2)°
Volume	2305.46(14) Å^3
Z, Density (calculated)	1, 1.235 g/cm^3
Absorption coefficient	1.061 mm^{-1}
F(000)	898.0
Crystal size	0.1 x 0.1 x 0.02 mm^3
Theta range for data collection	5.42 to 133.748 deg.
Index ranges	-14<=h<=14, -14<=k<=14, -20<=l<=20
Reflections collected / unique	50625 / 8161 [R(int) = 0.0776]
Completeness to theta = 66.874 deg	99.4 %
Absorption correction	Semi-empirical from equivalents
Max. and min. transmission	0.753 and 0.681
Refinement method	Full-matrix least-squares on F^2
Data / restraints / parameters	8161 / 0 / 597
Goodness-of-fit on F^2	1.052
Final R indices [I>2sigma(I)]	R_1 = 0.0769, wR_2 = 0.2041
R indices (all data)	R_1 = 0.1183, wR_2 = 0.2423
Largest diff. peak and hole	0.55 and -0.88 e.Å^{-3}

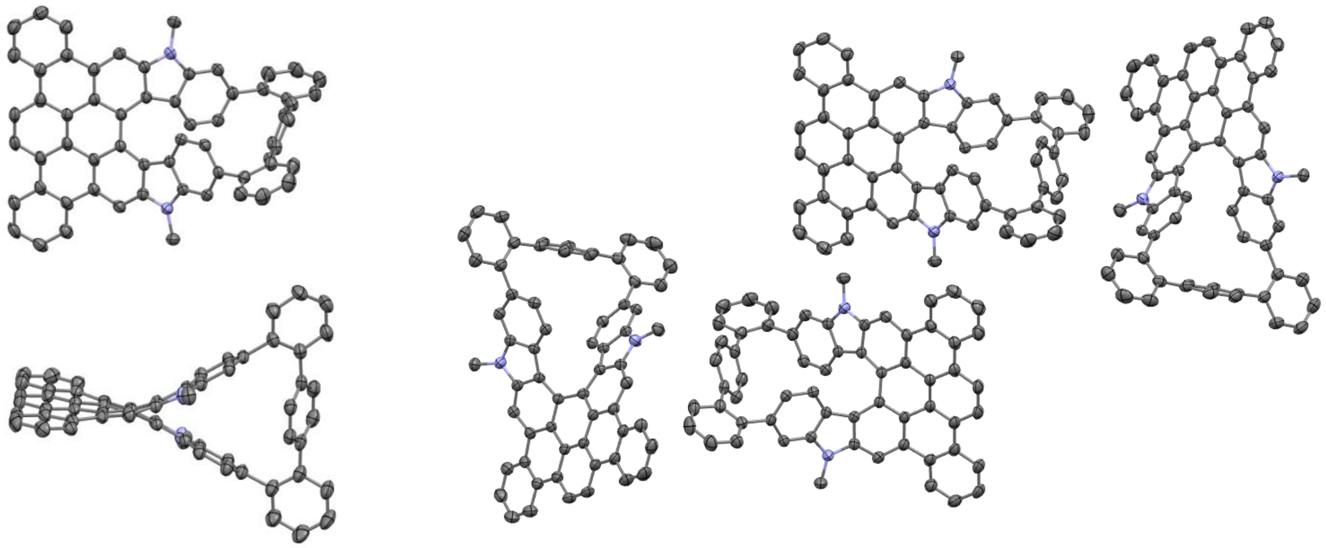

Figure S2. Molecular structure of compound **3**.

Table S2. Crystal data and structure refinement for **3**

Identification code	cu_129, 2500173
Empirical formula	$C_{62}H_{36}N_2$
Formula weight	808.93
Temperature	190 K
Wavelength	1.54178 Å
Crystal system, space group	Monoclinic, P 1 21 / n 1
Unit cell dimensions	a = 5.49450(10) Å α = 90º
	b = 18.1564(5) Å β = 90.563(2)º
	c = 39.0059(10) Å γ = 90º
Volume	3891.05(16) $Å^3$
Z, Density (calculated)	4, 1.381 g/cm^3
Absorption coefficient	0.611 mm^{-1}
F(000)	1688.0
Theta range for data collection	4.53 to 136.722 deg.
Index ranges	-5<=h<=6, -21<=k<=21, -47<=l<=46
Reflections collected / unique	117423 / 7159 [R(int) = 0.0584]
Completeness to theta = 68.361 deg	100.0 %
Absorption correction	Semi-empirical from equivalents
Max. and min. transmission	0.753 and 0.680
Refinement method	Full-matrix least-squares on F^2
Data / restraints / parameters	7159 / 0 / 579
Goodness-of-fit on F^2	1.028
Final R indices [I>2sigma(I)]	R_1 = 0.0473, wR_2 = 0.1278
R indices (all data)	R_1 = 0.0615, wR_2 = 0.1395
Largest diff. peak and hole	0.42 and -0.25 e.$Å^{-3}$

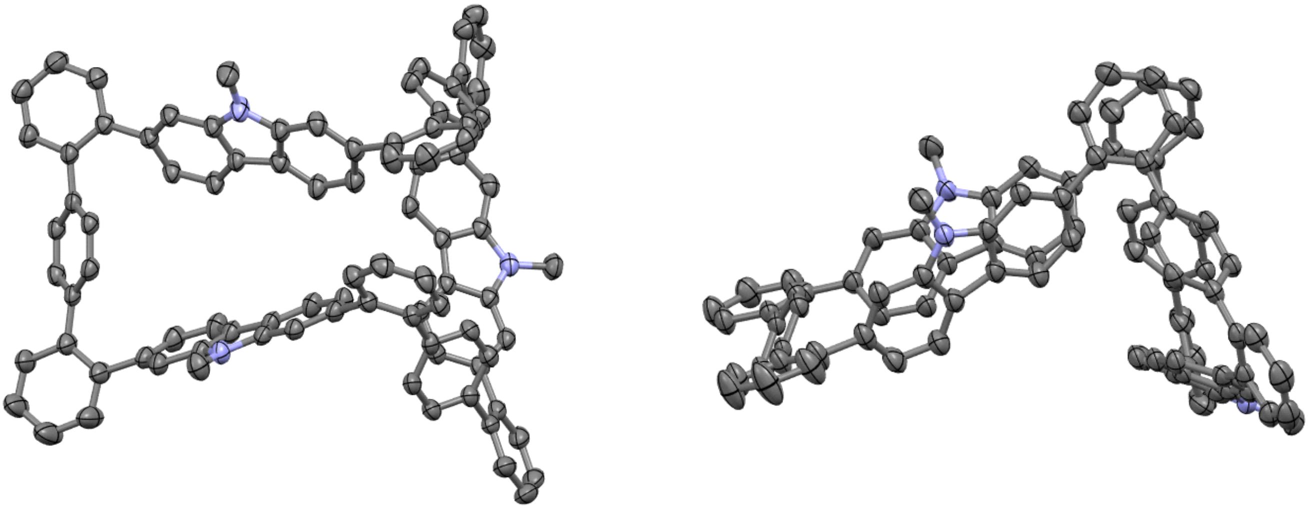

Figure S3. Molecular structure of compound **2**.

Table S3. Crystal data and structure refinement for **2**

Identification code	cu_250905_Xu_3plus3, 2500174
Empirical formula	$C_{110.5}H_{83}N_3$ ($C_{93}H_{63}N_3$, 2.5 C_7H_8)
Formula weight	1452.79
Temperature	190 K
Wavelength	1.54178 Å
Crystal system, space group	Triclinic, P-1
Unit cell dimensions	a = 15.127(2) Å α = 74.799(7)°
	b = 17.506(2) Å β = 85.325(7)°
	c = 17.965(3) Å γ = 66.896(7)°
Volume	4221.4(10) $Å^3$
Z, Density (calculated)	2, 1.143 g/cm^3
Absorption coefficient	0.498 mm^{-1}
F(000)	1534.0
Crystal size	0.08 x 0.06 x 0.05 mm^3
Theta range for data collection	5.098 to 145.62 deg.
Index ranges	-16<=h<=18, -21<=k<=21, -22<=l<=22
Reflections collected / unique	66310 / 16587 [R(int) = 0.0738]
Completeness to theta = 72.810 deg	98.5 %
Absorption correction	Semi-empirical from equivalents
Max. and min. transmission	0.752 and 0.682
Refinement method	Full-matrix least-squares on F^2
Data / restraints / parameters	16587 / 0 / 868
Goodness-of-fit on F^2	1.014
Final R indices [I>2sigma(I)]	R_1 = 0.0766, wR_2 = 0.2326
R indices (all data)	R_1 = 0.1011, wR_2 = 0.2570
Largest diff. peak and hole	0.27 and -0.33 e.$Å^{-3}$

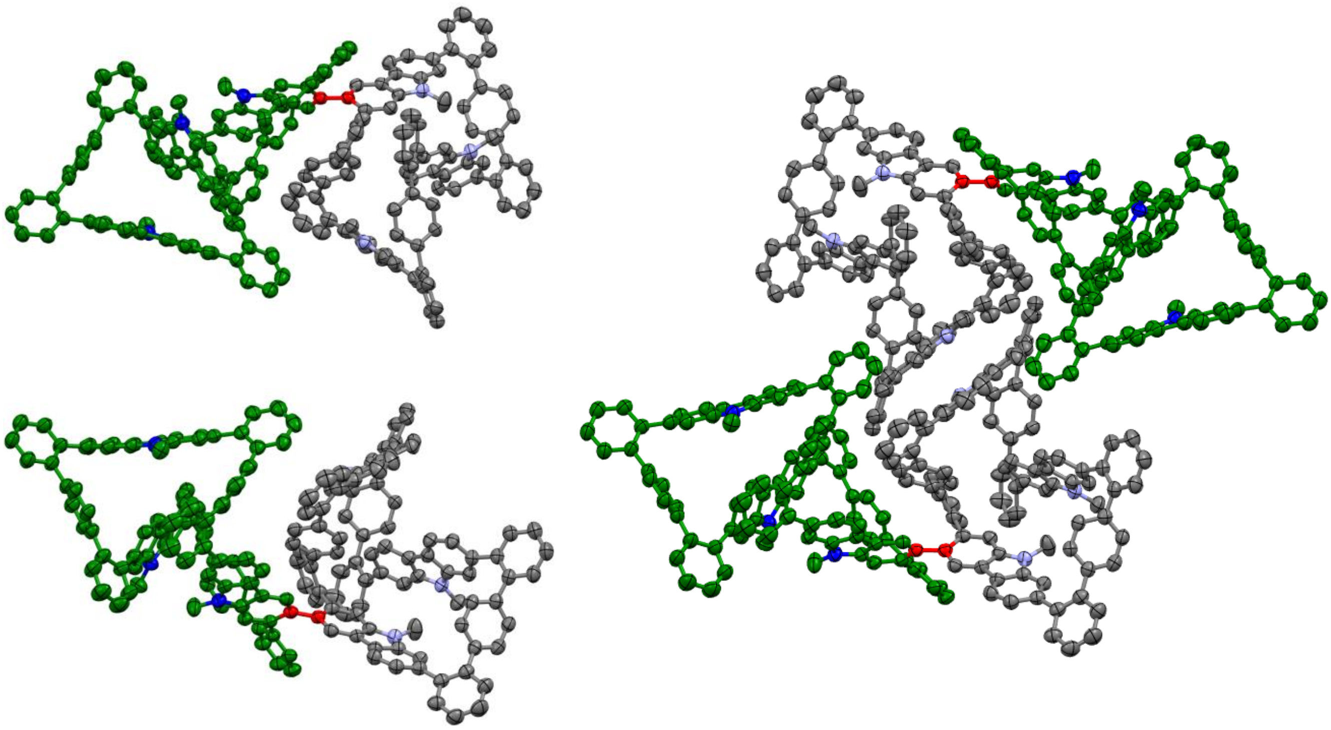

Figure S4. Molecular structure of compound **1**.

Table S4. Crystal data and structure refinement for **1**

Identification code	cu_250909_Xu_3plus3_school_7th, 2500175
Empirical formula	$C_{238.5}H_{184}N_6$ ($C_{186}H_{124}N_6$, 7.5 C_7H_8)
Formula weight	3133.91
Temperature	150 K
Wavelength	1.54178 Å
Crystal system, space group	Triclinic, P-1
Unit cell dimensions	a = 17.8150(13) Å α = 69.443(4)°
	b = 21.1806(15) Å β = 84.326(4)°
	c = 24.6957(17) Å γ = 79.714(4)°
Volume	8578.3(11) $Å^3$
Z, Density (calculated)	2, 1.213 g/cm^3
Absorption coefficient	0.527 mm^{-1}
F(000)	3314.0
Crystal size	0.08 x 0.08 x 0.06 mm^3
Theta range for data collection	4.51 to 146.068 deg.
Index ranges	-21<=h<=21, -26<=k<=26, -28<=l<=30
Reflections collected / unique	216702 / 33785 [R(int) = 0.1113]
Completeness to theta = 73.034 deg	98.4 %
Absorption correction	Semi-empirical from equivalents
Max. and min. transmission	0.754 and 0.668
Refinement method	Full-matrix least-squares on F^2
Data / restraints / parameters	33785 / 1 / 1735
Goodness-of-fit on F^2	0.910
Final R indices [I>2sigma(I)]	R_1 = 0.0786, wR_2 = 0.1849
R indices (all data)	R_1 = 0.1368, wR_2 = 0.2299
Largest diff. peak and hole	0.22 and -0.36 e.$Å^{-3}$

2.3 DFT computations

Method

All calculations were performed using the ORCA software package (version 6.1.0).[1] Geometry optimizations were carried out at the GFN2-XTB[2] level of theory with the ALPB implicit solvation for DCM solvent. Frequency analyses were conducted to ensure no imaginary frequencies for all stable compounds and only one imaginary frequency for each transition state. To obtain more accurate electronic energies, single-point energy calculations were performed at the higher-level wB97M-V[3]/def2-TZVPP[4] level of theory (and wB97M-V[3]/def2-TZVP[4] level of theory with gCp[5] correction for the C_{70} interactions) with the SMD[6] implicit solvation model was employed for all compounds for the DCM solvent effects.

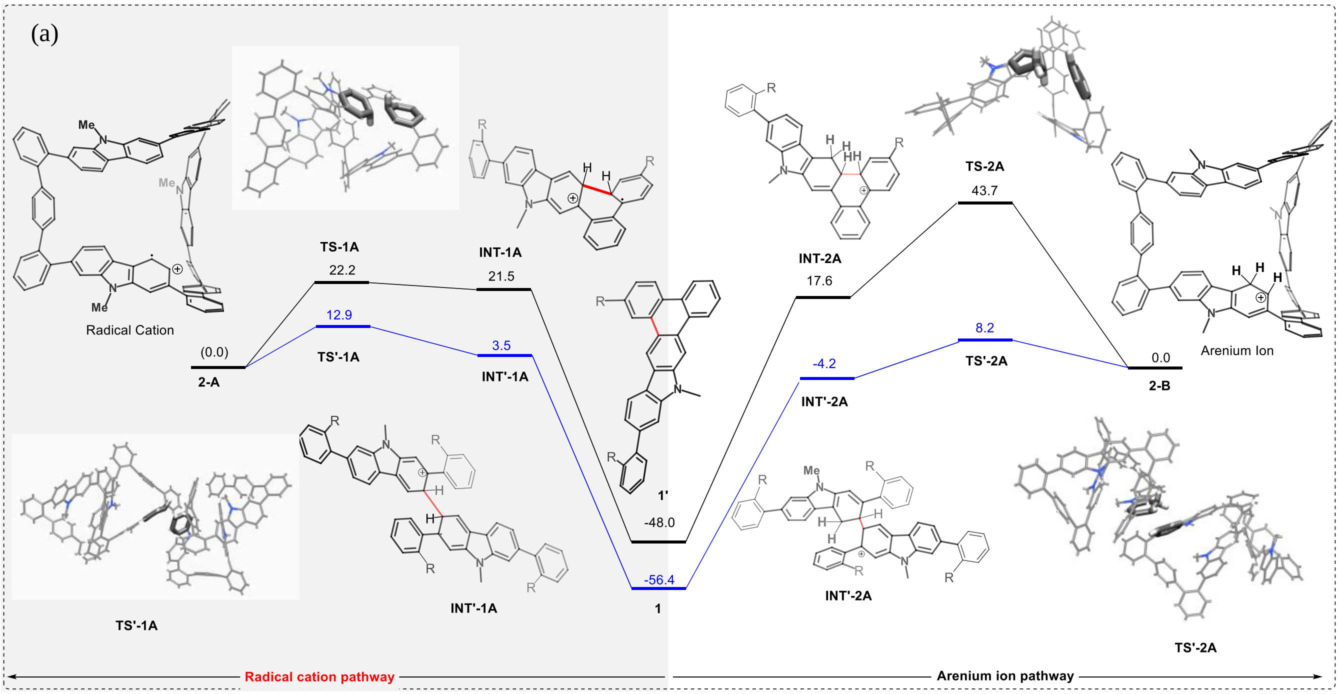

(b)

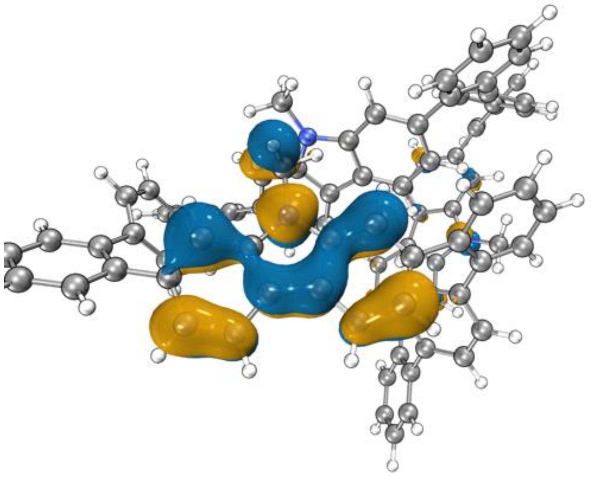

(c)

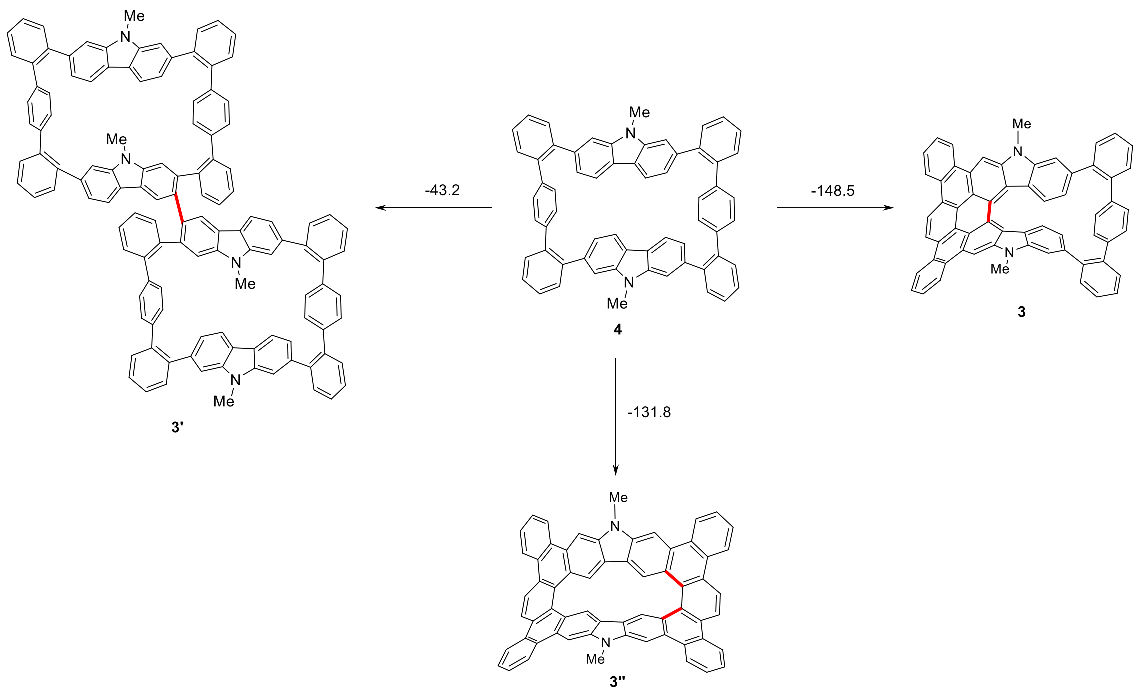

Figure S5. (a) Calculated free energy profile for the formation of compounds **1** and **1’**, the relative free energies are given in kcal/mol; (b) The highest occupied orbital molecular (HOMO) of compound **2**; (c) DFT for the selectivity of the Scholl reactions for compound **4**.

a)

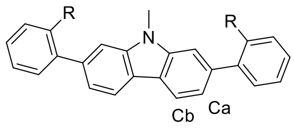

b)

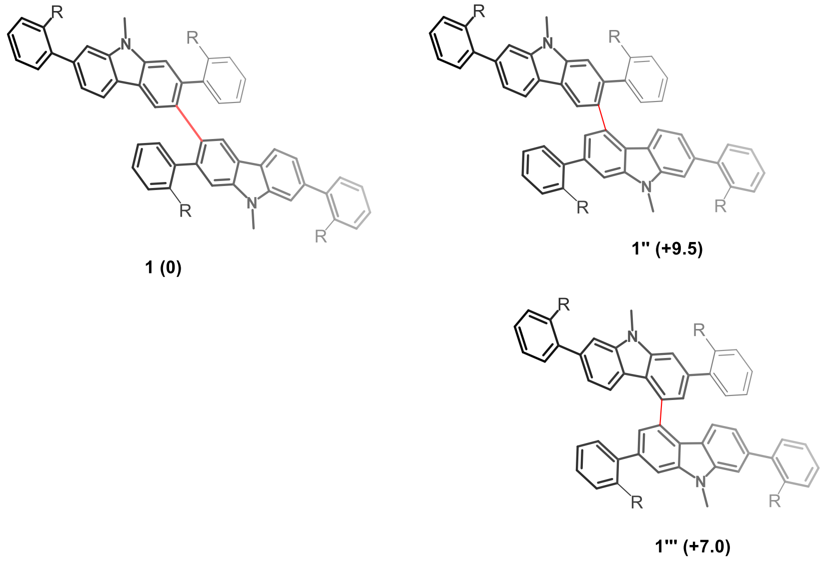

Figure S6. (a) The distinct two carbon site for intermolecular couplings; (b) DFT for the selectivity of the Scholl reactions for compound **1** to the other reaction sites.

The Job's plot analysis indeed indicates a 1:1 stoichiometry between bismacrocycle **1** and C_{70}. To determine whether the binding mode involves sandwich-type encapsulation or partial encapsulation, we performed computational evaluations on three possible binding geometries:

- cp1: C_{70} interacts primarily with the carbazole units.
- cp2: C_{70} interacts mainly with the triphenyl units.
- cp3: C_{70} is partially encapsulated through cooperative interactions with both carbazole and phenyl units.

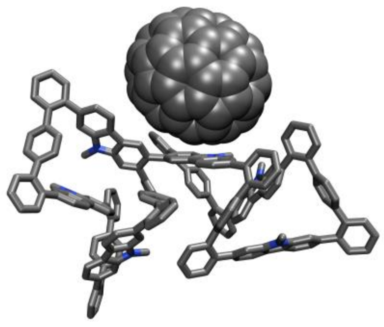

Figure S7. Molecular structure of cp1.

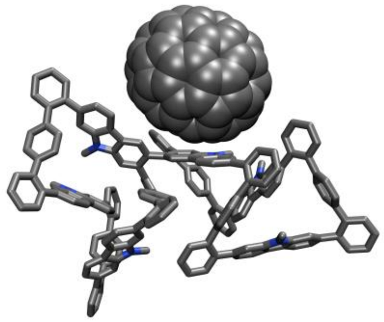

Figure S8. Molecular structure of cp2.

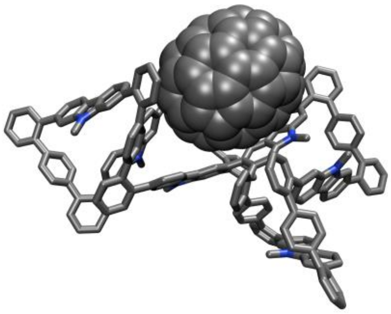

Figure S9. Molecular structure of cp3.

Table S5. The calculated interaction energies (E) clearly favor the partial encapsulation model.

**Binding mode**	**E (kcal/mol)**	**ΔG (kcal/mol)**
cp1 (carbazole-only)	–23.7	–8.4
cp2 (triphenyl-only)	–12.9	–2.0
cp3 (partial encapsulation)	–41.0	–12.4

Cp3 exhibits the most negative interaction energy and Gibbs free energy change, indicating it is thermodynamically the most favorable binding motif. Therefore, the computational results suggest that the binding mode is partial encapsulation of C_{70} within the bismacrocyclic cavity, rather than a sandwich-type structure.

2.4 Photophysical properties of the macrocyclic compounds 4, 3, 2 and 1

2.4.1 UV−vis absorption and fluorescence spectra of 4, 3, 2 and 1

The photophysical properties of the macrocycle compounds were also investigated, which solution in CH_2Cl_2 (concentration $2.5*10^{-5}$ M) at room temperature. (a) It showed absorption maxima (λ_{abs}) at 262 nm and 322 nm, along with an emission band at 404 nm in CH_2Cl_2 solution of macrocycle **4**, 2+2; (b) λ_{abs} at 278 nm and 360 nm, λ_{em} at 478 nm and 503 nm of **3**, 2+2 Scholl; (c) λ_{abs} at 259 nm and 321 nm, λ_{em} at 401 nm of **2**, 3+3; (d) λ_{abs} at 259 nm and 321 nm, λ_{em} at 409 nm of **1**, 3+3 Scholl, which might have different electron transitions or contain different chromophores.

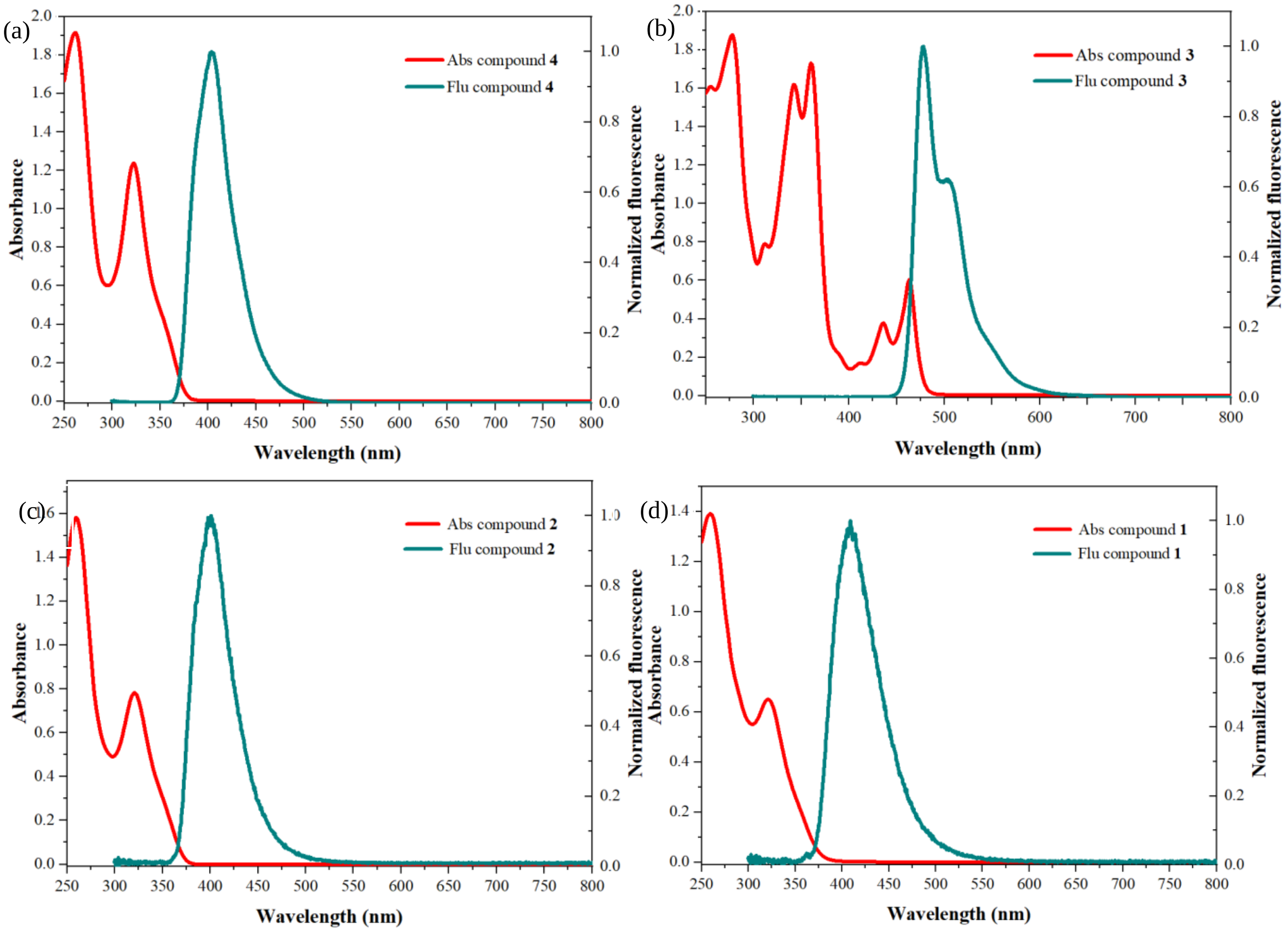

Figure S10. (a-d) UV–*vis* absorption (red lines) and emission spectra (green lines) in CH_2Cl_2 (2.5×10^{-5} M) at room temperature.

2.4.2 Quantum yield of 4, 3, 2 and 1

The fluorescence quantum yields (Φ) were determined by comparing the photoluminescence integrated intensities and absorbance intensities with a standard, perylene (Φ = 0.92).[7] The quantum yield was the calculated by using the following equation:[8]

$$\Phi_s = (F_s/F_r)(A_r/A_s)(\eta_s/\eta_r)^2\Phi_r$$

Where F is the integrated intensities (area under emission peak), A is the absorbance, η is the refractive index and Φ is the quantum yield, the subscript s and r refer to sample and reference, respectively.

Perylene was dissolved in EtOH (η = 1.36), while **4**, **3**, **2** and **1** were dissolved in CH_2Cl_2 (η = 1.42). Absorbance values were kept below 0.06 at the excitation wavelength in order to minimize reabsorption effects.

Table S6. Quantum yield of 4, 3, 2 and 1

**Compounds**	**Integrated intensity (*F*)**	**Absorbance at 361 nm (*A*)**	**Refractive index (*η*)**	**Quantum yield (*Φ*)**
**Perylene**	195264.3	0.033	1.36	0.92
**4**	82942.1	0.036	1.42	0.39
**3**	70462.3	0.030	1.42	0.40
**2**	128702.6	0.056	1.42	0.39
**1**	92755.86	0.044	1.42	0.36

2.4.3 Luminescent lifetimes of 4 and 3

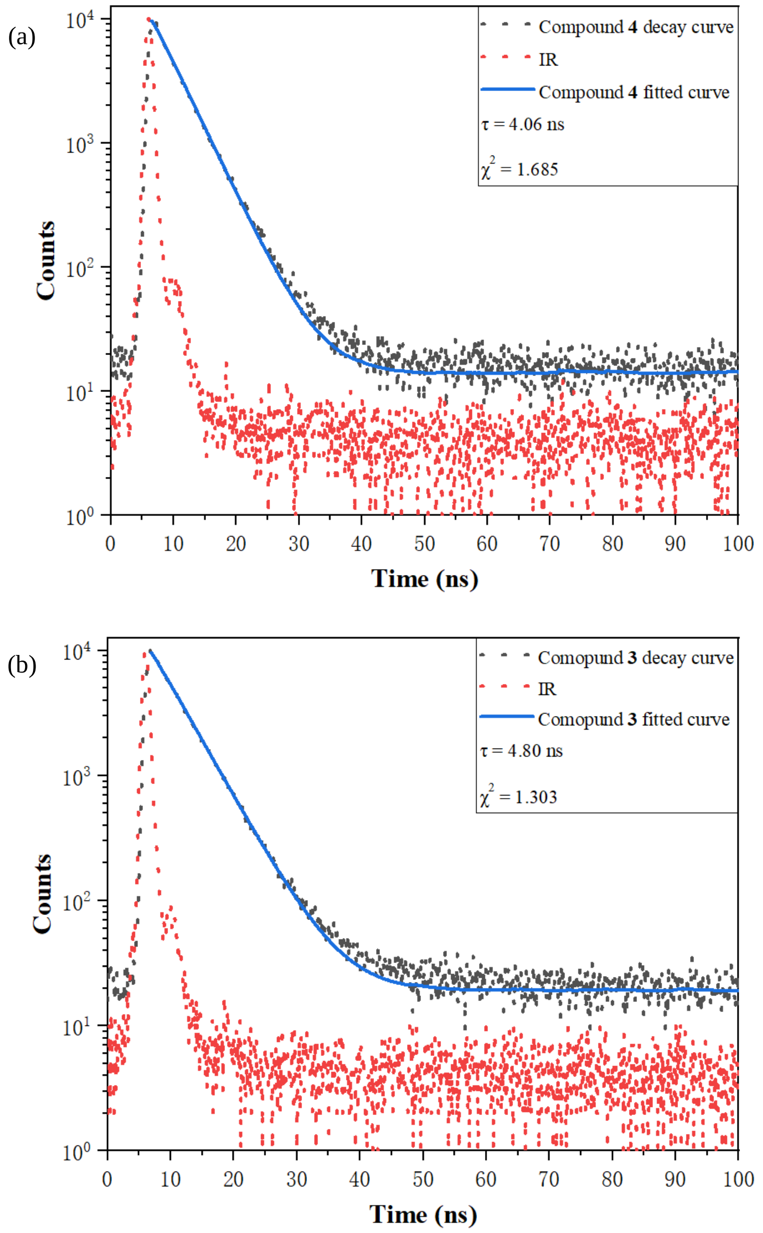

Figure S11. (a) Luminescent lifetimes (λ_{ex} = 373 nm) of macrocycle **4** measured at 477 nm in the liquid state. (b) Luminescent lifetimes (λ_{ex} = 365 nm) of macrocycle **3** measured at 404 nm in the liquid state.

2.5 The host-guest interactions between 3 and 1 with C_{70}

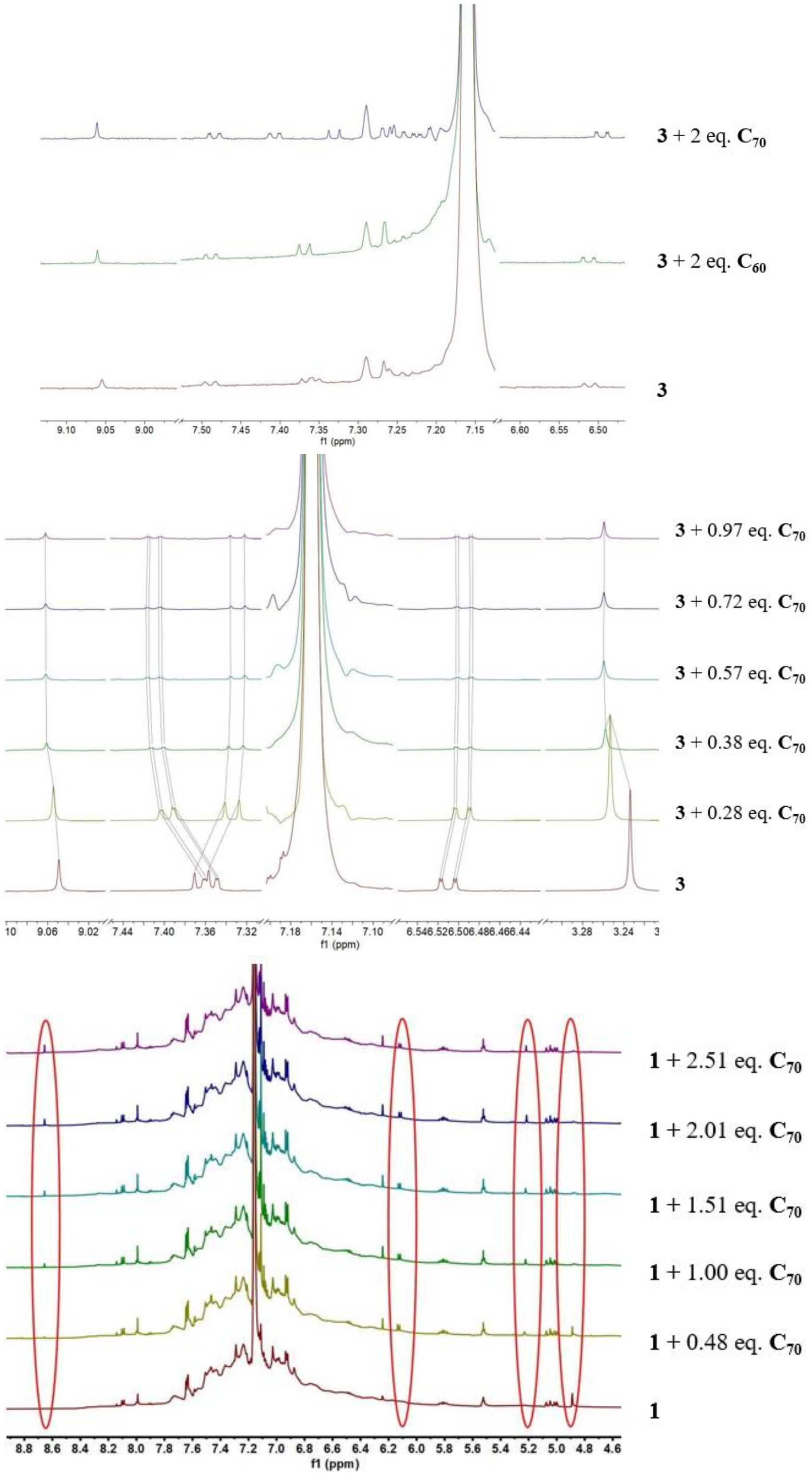

Figure S12. The NMR titration between **3** and **1** with C_{70} in C_6D_6.

2.6 Fluorescent titration and Job's plot experiments

(a) Fluorescent spectra (Ex = 322 nm) of **4** ($2*10^{-6}$ M) titrated with C_{70}, where the concentration range of C_{70} is 0 to $2.9*10^{-4}$ M; (b) Changes of fluorescent intensity at 404 nm were used for nonlinear fitting with K_a of $7.0*10^4$ M^{-1}; (c, d) Job's plot based on the UV spectra changes at 472 nm for complexation between **4** and C_{70} in 1,2-dichlorobenzene. (Procedure: A solution of **4** in 1,2-dichlorobenzene and a solution of fullerene C_{70} in 1,2-dichlorobenzene were mixed in different ratios to prepare 12 samples with a fixed total concentration (c(**4**) + c(C_{70}) = $4*10^{-6}$ M). The UV absorbance of each sample was measured, and the changes in absorbance intensity (at 472 nm) were monitored for Job's plot analysis. The results indicated that **4** formed 1:1 host–guest complexes with C_{70} in 1,2-dichlorobenzene.

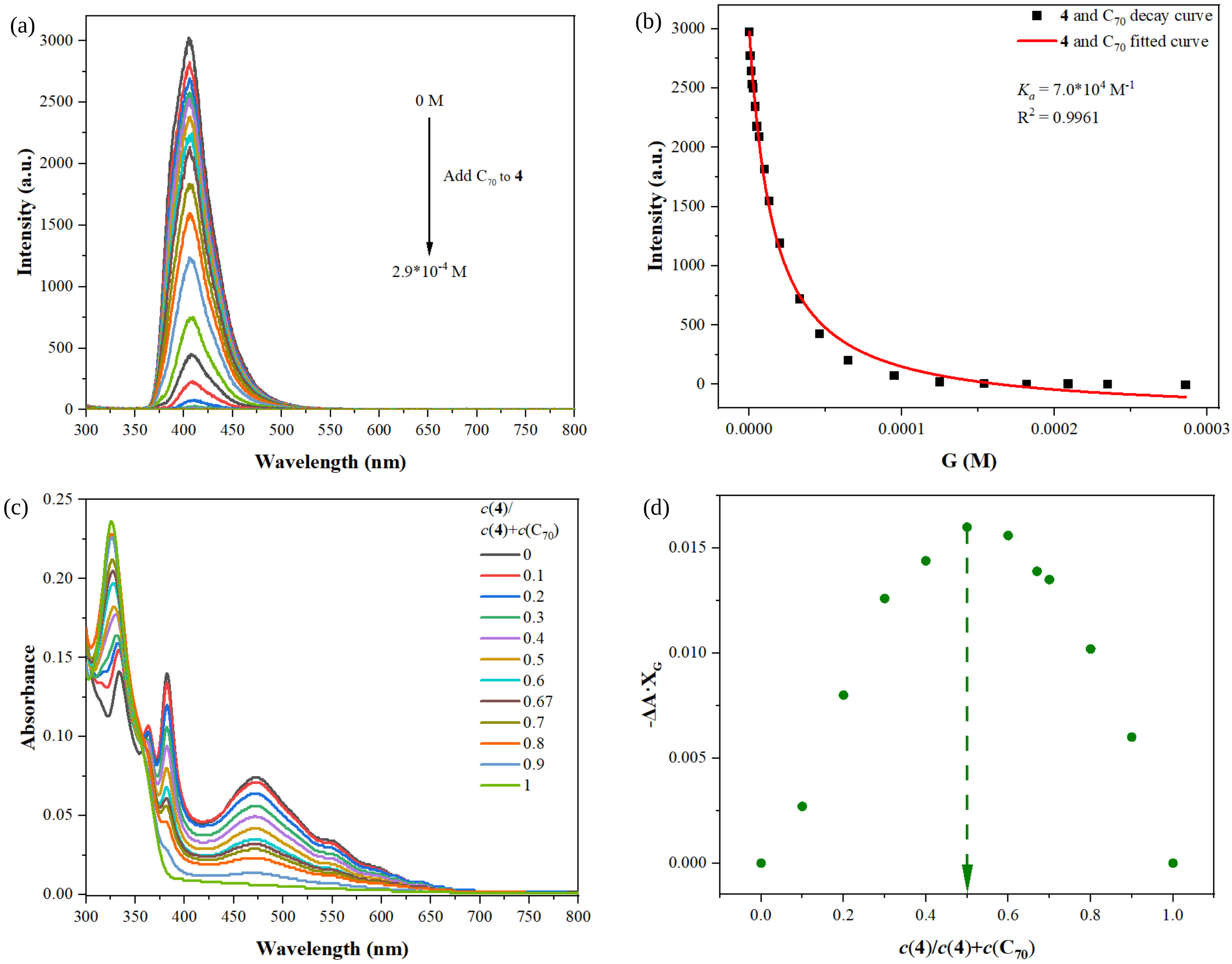

Figure S13. (a-d) Fluorescent titration and Job's plot experiments: fullerene C_{70} binding behavior of **4** in 1,2-dichlorobenzene.

(a) Fluorescent spectra (Ex = 361 nm) of **3** ($2*10^{-6}$ M) titrated with C_{70}, where the concentration range of C_{70} is 0 to $2.9*10^{-4}$ M; (b) The changes in fluorescent intensity at 478 nm were used for nonlinear fitting with K_a of $7.3*10^4$ M^{-1}; (c, d) Job's plot based on the UV spectra changes at 383 nm for complexation between **3** and C_{70} in 1,2-dichlorobenzene (c(**3**) + c(C_{70}) = $4.9*10^{-6}$ M). The UV absorbance of each sample was measured, and the changes in absorbance intensity (at 383 nm) were monitored for Job's plot analysis. The results indicated that **3** Scholl formed 1:1 host–guest complexes with C_{70} in 1,2-dichlorobenzene.

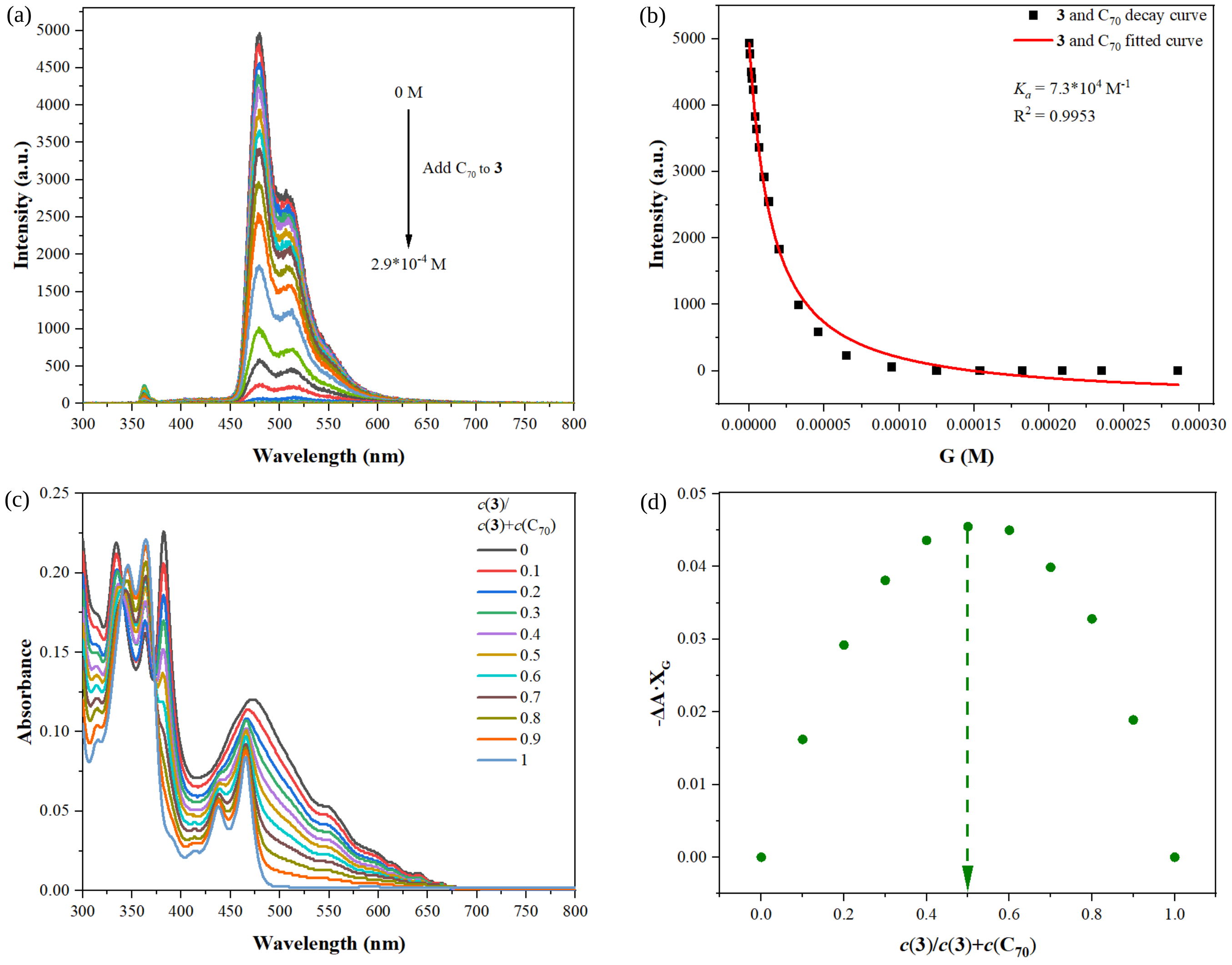

Figure S14. (a-d) Fluorescent titration and Job's plot experiments: fullerene C_{70} binding behavior of **3** in 1,2-dichlorobenzene.

(a) Fluorescent spectra (Ex = 321 nm) of **2** ($2*10^{-6}$ M) titrated with C_{70}, where the concentration range of C_{70} is 0 to $3.3*10^{-4}$ M; (b) The changes in fluorescent intensity at 401 nm were used for nonlinear fitting with K_a of $5.6*10^4$ M^{-1}; (c, d) Job's plot based on the UV spectra changes at 472 nm for complexation between **2** and C_{70} in 1,2-dichlorobenzene (c(**2**) + $c(C_{70})$ = $4*10^{-6}$ M). The UV absorbance of each sample was measured, and the changes in absorbance intensity (at 472 nm) were monitored for Job's plot analysis. The results indicated that **2** formed 1:1 host−guest complexes with C_{70} in 1,2-dichlorobenzene.

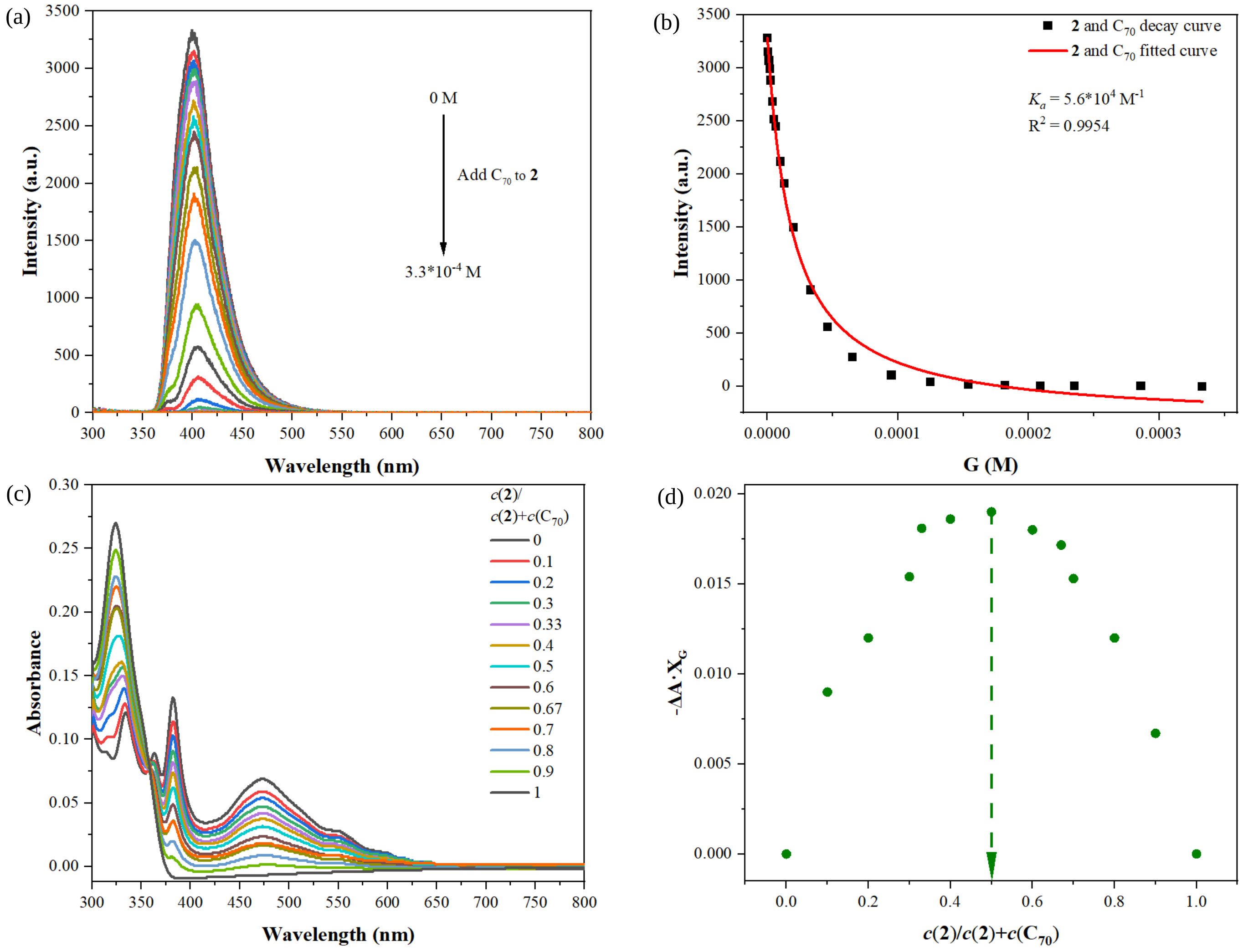

Figure S15. (a-d) Fluorescent titration and Job's plot experiments: fullerene C_{70} binding behavior of **2** in 1,2-dichlorobenzene.

(a) Fluorescent spectra (Ex = 321 nm) of **1** ($2*10^{-6}$ M) titrated with C_{70}, where the concentration range of C_{70} is 0 to $2.9*10^{-4}$ M; (b) The changes in fluorescent intensity at 407 nm were used for nonlinear fitting with K_a of $6.4*10^4$ M^{-1}; (c, d) Job's plot based on the UV spectra changes at 472 nm for complexation between **1** and C_{70} in 1,2-dichlorobenzene (c(**1**) + $c(C_{70})$ = $4*10^{-6}$ M). The UV absorbance of each sample was measured, and the changes in absorbance intensity (at 472 nm) were monitored for Job's plot analysis. The results indicated that **1** formed 1:1 host−guest complexes with C_{70} in 1,2-dichlorobenzene.

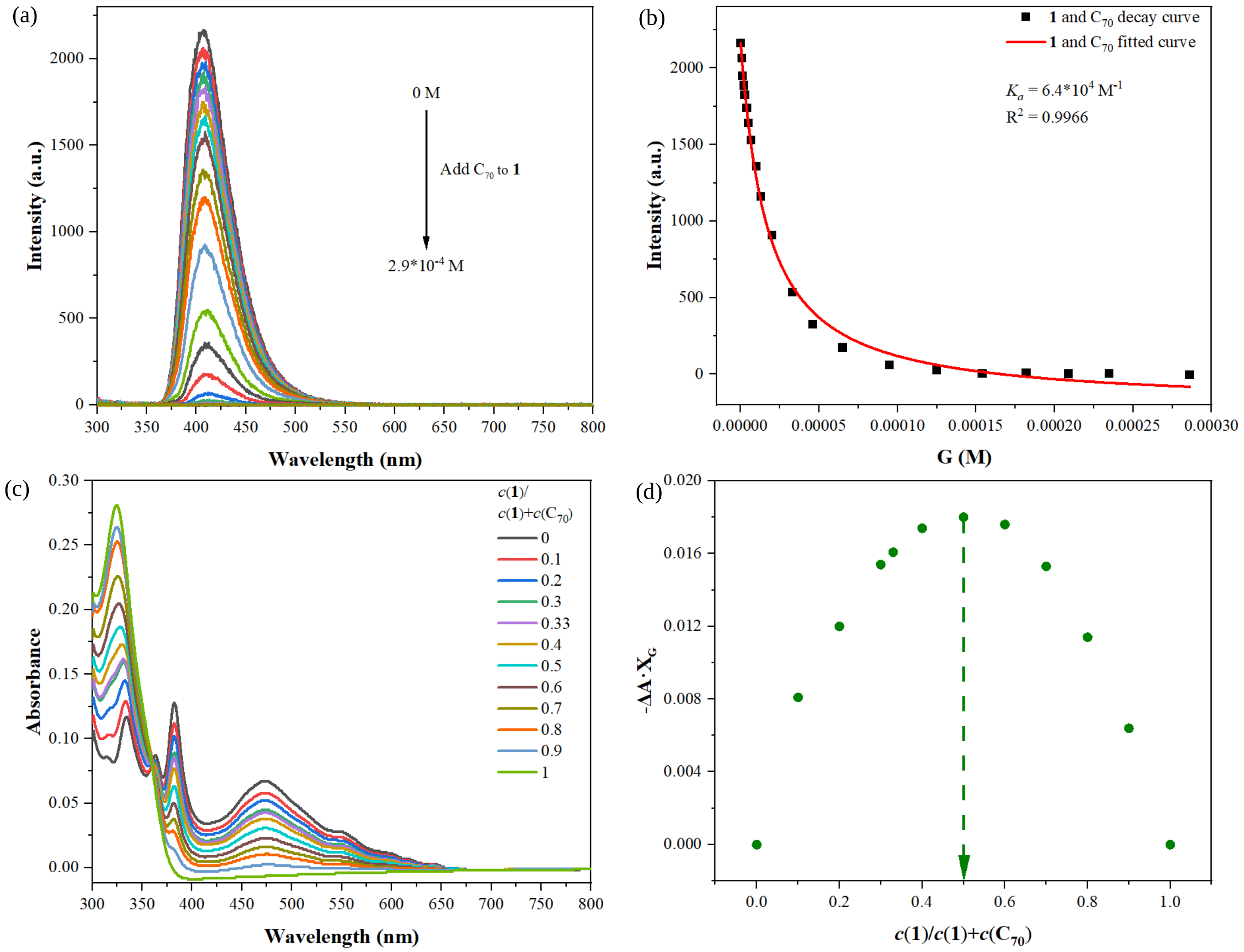

Figure S16. (a-d) Fluorescent titration and Job's plot experiments: fullerene C_{70} binding behavior of **1** in 1,2-dichlorobenzene.

2.7 Visible light-driven, biamacrocycle 1 catalyzed borylation of aryl halides

2.7.1 Screening of the reaction conditions

By means of the reaction of 1-bromo-4-methoxybenzene (**6a**) with bis(pinacolato)diboron as the model reaction, the reaction conditions were optimized as follows.

Table S7. Screening of the reaction conditions of 6a and bis(pinacolato)diboron

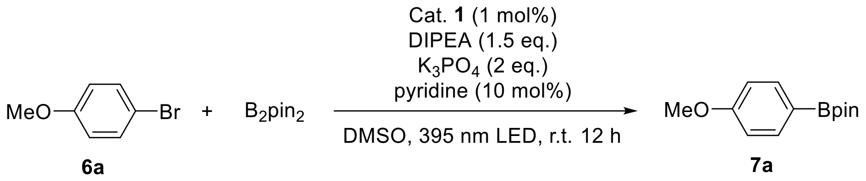

Entry[a]	Variation from conditions	Yield of **7a**[b] (%)
1	none	72
2	Cat. **4** instead of Cat. **1**	9
3	Cat. **3** instead of Cat. **1**	61
4	Cat. **2** instead of Cat. **1**	30
5	365 nm instead of 395 nm	37
6	No light	0

[a]Conditions: **6a** (0.3 mmol), B_2Pin_2 (0.6 mmol), Cat. **1** (0.003 mmol), DIPEA (0.45 mmol), K_3PO_4 (0.6 mmol), pyridine (0.03 mmol), DMSO (1 mL), 395 nm LED, r.t., 12 h. [b]The yields were determined by ^1H NMR analysis with CH_2Br_2 as the internal standard.

2.7.2 General procedure for the synthesis of aryl boronate (7)

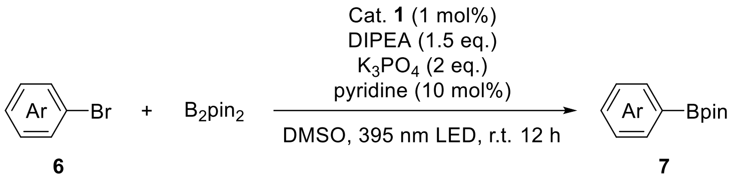

A typical procedure for the synthesis of 2-(4-methoxyphenyl)-4,4,5,5-tetramethyl-1,3,2-dioxaborolane (7a): A mixture of 1-bromo-4-methoxybenzene (56 mg, 0.3 mmol), B_2Pin_2 (152 mg, 0.6 mmol), Cat. **1** (7.3 mg, 0.003 mmol), DIPEA (58 mg, 0.45 mmol), K_3PO_4 (127 mg, 0.6 mmol), and pyridine (2.4 mg, 0.03 mmol) in 1 mL DMSO was stirred at 25 °C for 12 h under a N_2 atmosphere under 395 nm LED irradiation. The reaction mixture was poured into water (10 mL) and extracted with DCM (20 mL). The organic layer was separated and dried over anhydrous sodium sulfate, and all volatiles were then evaporated under reduced pressure. The resulting mixture was purified by column chromatography on silica gel (eluent: hexane/dichloromethane = 3:1, v/v), yielding compound **7a** as a colorless liquid (51 mg, 73%).

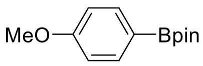

7a

^{1}H NMR (600 MHz, $CDCl_3$) δ 7.76 (d, J = 8.6 Hz, 2H), 6.90 (d, J = 8.6 Hz, 2H), 3.83 (s, 3H), 1.34 (s, 12H) ppm; **^{13}C NMR** (151 MHz, $CDCl_3$) δ 162.28, 136.64, 113.44, 83.67, 55.21, 24.99 ppm. HRMS (EI) *m/z* calcd for $C_{13}H_{20}BO_3$ $[M+H]^+$: 235.1506; Found: 235.1494.

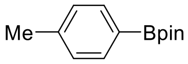

7b

4,4,5,5-Tetramethyl-2-(*p*-tolyl)-1,3,2-dioxaborolane (7b): 34 mg, 52% yield, colorless solid. **^{1}H NMR** (400 MHz, $CDCl_3$) δ 7.71 (d, J = 7.9 Hz, 2H), 7.19 (d, J = 7.4 Hz, 2H), 2.37 (s, 3H), 1.34 (s, 12H) ppm; **^{13}C NMR** (101 MHz, $CDCl_3$) δ 141.54, 134.94, 128.66, 83.77, 25.00, 21.87 ppm. HRMS (EI) *m/z* calcd for $C_{13}H_{20}BO_2$ $[M+H]^+$: 219.1556; Found: 219.1552.

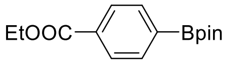

7c

Ethyl 4-(4,4,5,5-tetramethyl-1,3,2-dioxaborolan-2-yl)benzoate (7c): 50.4 mg, 61% yield, colorless solid. **^{1}H NMR** (600 MHz, $CDCl_3$) δ 8.00 (d, J = 8.0 Hz, 2H), 7.85 (d, J = 8.1 Hz, 2H), 4.38 – 4.34 (m, 2H), 1.39 – 1.36 (m, 3H), 1.33 (s, 12H) ppm; **^{13}C NMR** (151 MHz, $CDCl_3$) δ 166.72, 134.71, 128.63, 84.23, 83.56, 61.10, 24.96, 14.40 ppm. HRMS (EI) *m/z* calcd for $C_{15}H_{22}BO_4$ $[M+H]^+$: 277.1611; Found: 277.1613.

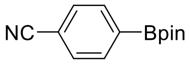

7d

4-(4,4,5,5-Tetramethyl-1,3,2-dioxaborolan-2-yl)benzonitrile (7d): 36.4 mg, 53% yield, colorless solid. **^{1}H NMR** (400 MHz, $CDCl_3$) δ 7.88 (d, J = 8.2 Hz, 2H), 7.64 (d, J = 8.2 Hz, 2H), 1.35 (s, 12H) ppm; **^{13}C NMR** (101 MHz, $CDCl_3$) δ 135.23, 131.27, 119.01, 114.68, 84.64, 25.00 ppm. HRMS (EI) *m/z* calcd for $C_{13}H_{17}BNO_2$ $[M+H]^+$: 230.1352; Found: 230.1348.

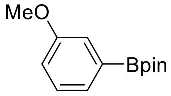

7e

2-(3-Methoxyphenyl)-4,4,5,5-tetramethyl-1,3,2-dioxaborolane (7e): 33.7 mg, 48% yield, colorless liquid. **^{1}H NMR** (600 MHz, $CDCl_3$) *δ* 7.42 – 7.40 (m, 1H), 7.34 (d, *J* = 2.8 Hz, 1H), 7.31 – 7.28 (m, 1H), 7.03 – 6.99 (m, 1H), 3.83 (s, 3H), 1.35 (s, 12H) ppm; **^{13}C NMR** (151 MHz, $CDCl_3$) *δ* 159.16, 129.04, 127.29, 118.84, 118.00, 83.92, 55.33, 24.97 ppm. HRMS (EI) *m/z* calcd for $C_{13}H_{20}BO_3$ $[M+H]^+$: 235.1506; Found: 235.1501.

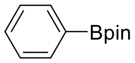

7g

4,4,5,5-tetramethyl-2-phenyl-1,3,2-dioxaborolane (7g): 30.6 mg, 50% yield, colorless liquid. **^{1}H NMR** (400 MHz, $CDCl_3$) *δ* 7.86 – 7.81 (m, 2H), 7.51 – 7.45 (m, 1H), 7.41 – 7.36 (m, 2H), 1.37 (s, 12H) ppm; **^{13}C NMR** (101 MHz, $CDCl_3$) *δ* 134.87, 131.37, 127.83, 83.88, 24.99 ppm. HRMS (EI) *m/z* calcd for $C_{12}H_{18}BO_2$ $[M+H]^+$: 205.1400; Found: 205.1396.

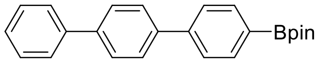

7h

2-([1,1':4',1''-Terphenyl]-4-yl)-4,4,5,5-tetramethyl-1,3,2-dioxaborolane (7h): 72.7 mg, 68% yield, colorless liquid. **^{1}H NMR** (300 MHz, $CDCl_3$) *δ* 7.95 – 7.88 (m, 2H), 7.74 – 7.62 (m, 8H), 7.51 – 7.43 (m, 2H), 7.40 – 7.33 (m, 1H), 1.38 (s, 12H) ppm; **^{13}C NMR** (151 MHz, $CDCl_3$) *δ* 143.49, 140.83, 140.60, 140.04, 135.46, 128.97, 127.73, 127.69, 127.67, 127.53, 127.22, 126.47, 84.00, 25.04 ppm. HRMS (EI) *m/z* calcd for $C_{24}H_{26}BO_2$ $[M+H]^+$: 357.2026; Found: 357.2031.

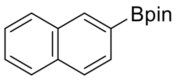

7i

4,4,5,5-Tetramethyl-2-(naphthalen-2-yl)-1,3,2-dioxaborolane (7i): 34.6 mg, 45% yield, colorless liquid. **^{1}H NMR** (600 MHz, $CDCl_3$) *δ* 8.50 – 8.42 (m, 1H), 7.96 – 7.90 (m, 2H), 7.89 – 7.84 (m, 2H), 7.57 – 7.49 (m, 2H), 1.43 (s, 12H) ppm; **^{13}C NMR** (151 MHz, $CDCl_3$) *δ* 136.38, 135.16, 132.94, 130.53, 128.75, 127.82, 127.10, 127.07, 125.90, 84.01, 25.02 ppm. HRMS (EI) *m/z* calcd for $C_{16}H_{20}BO_2$ $[M+H]^+$: 255.1556; Found: 255.1551.

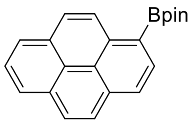

7j

4,4,5,5-Tetramethyl-2-(pyren-1-yl)-1,3,2-dioxaborolane (7j): 25.1 mg, 25% yield, yellow solid. **^{1}H NMR** (600 MHz, $CDCl_3$) *δ* 9.16 – 9.09 (m, 1H), 8.60 – 8.58 (m, 1H), 8.24 (d, *J* = 7.6 Hz, 1H), 8.21 – 8.17 (m, 3H), 8.13 (d, *J* = 8.9 Hz, 1H), 8.08 (d, *J* = 8.9 Hz, 1H), 8.04 – 8.01 (m, 1H), 1.53 (s, 12H) ppm; **^{13}C NMR** (151 MHz, $CDCl_3$) *δ* 136.58, 134.00, 133.59, 131.25, 130.91, 128.66, 128.17, 127.91, 127.61, 125.82, 125.47, 125.32, 124.75, 124.53, 124.21, 84.02, 25.19 ppm. HRMS (EI) *m/z* calcd for $C_{22}H_{22}BO_2$ $[M-H]^-$: 329.1718; Found: 329.1706.

2.7.3 Plausible mechanism for the borylation of aryl halides catalyzed by Cat. 1

A plausible mechanism for the borylation of aryl halides is proposed in Figure S14, the photo-excited Cat. **1*** is quenched by ArBr *via* a SET process, leading to the generation of a aryl radical species. The catalyst regenerates to its ground state *via* reductive quenching mediated by DIPEA, and borylation of the radical species furnishes the desired products ArBpin.

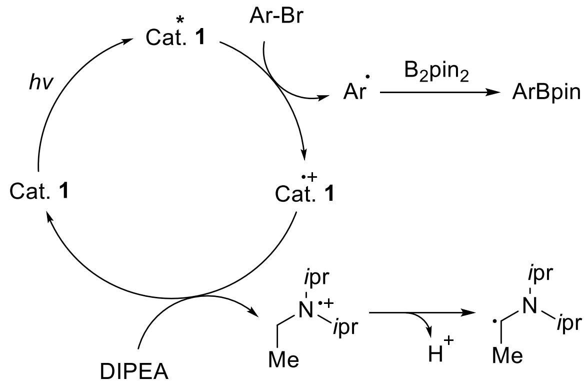

Figure S17. Mechanistic proposal for the borylation of aryl halides catalyzed by Cat. **1**.

2.8 Biological activity study

2.8.1 Cell culture conditions

Human lung carcinoma A549, human breast cancer MDA-MB-231, and human cervical carcinoma HeLa cells were cultured in Dulbecco's Modified Eagle Medium (DMEM) containing 10% FBS and 100 units of penicillin/streptomycin at 37 °C with 5% CO_2. Human fetal lung fibroblast WI-38 cells were cultured in Minimum Essential Medium (MEM) containing 10% FBS, 20 mM L-glutamine, 10 mM sodium pyruvate, 1% Non-Essential Amino Acids Solution (NEAA), and 100 units of penicillin/streptomycin at 37 °C with 5% CO_2. Unless otherwise mentioned, all media applied in cell treatments contained 1 % DMF.

2.8.2 Cytotoxicity test in the dark

A549, MDA-MB-231, and WI-38 cells were seeded into 96-well plates at a density of 2,000 cells per well and incubated for 48 h. The cells were treated with testing compounds (up to 50 μM) for 72 h. The compound-containing media were replaced with an FBS-free medium containing 3-(4,5-dimethylthiazol-2-yl)-2,5-diphenyltetrazolium bromide (MTT, 1 mg/mL), and the plates were further incubated for 1 h. The MTT-containing medium was then removed, and 100 μL per well of DMSO was added to dissolve the formazan crystal. The IC_{50} value was calculated based on the absorbance at 570 and 730 nm, which was measured by a Biotek microplate reader, and presented as the average of results from three independent experiments (n = 3).

Table S8. Dark cytotoxicity

**Compounds/ IC_{50} (μM)**	**A549**	**MDA-MB-231**	**WI-38**
Compound **4**	>50	>50	>50
Compound **3**	>50	>50	>50
Compound **2**	>50	>50	>50
Compound **1**	>50	>50	>50
Cisplatin	5.9±0.6	3.7±1.2	2.0±0.5
Doxorubicin	0.65±0.07	0.49±0.09	0.34±0.10

2.8.3 Photocytotoxicity tests

A549 and HeLa cells were seeded into 96-well plates at a density of 2,000 cells per well and incubated for 48 h. The cells were treated with testing compounds (up to 50 μM) for 6 h. The compound-containing media were replaced with a phenol-red-free medium. The cells were irradiated with a white LED (400-700 nm, 7.6 mW/cm²) for 1 h and then incubated for another 65 h. The cells were then incubated with MTT (1 mg/mL) for 1 h. The MTT-containing medium was then removed, and 100 μL per well of DMSO was added to dissolve the formazan crystal. The IC_{50} value was calculated based on the absorbance at 570 and 730 nm, which was measured by a Biotek microplate reader, and presented as the average of results from three independent experiments (n = 3).

Table S9. Photocytotoxicity for 72 h

Compounds/ IC_{50} (μM)	A549		HeLa	
	In the dark	With irradiation	In the dark	With irradiation
Compound **4**	>50	29.8±1.0	>50	33.9±7.0
Compound **3**	>50	29.4±2.8	>50	27.4±2.4
Compound **2**	>50	30.8±3.1	>50	>50
Compound **1**	>50	>50	>50	>50
Cisplatin	6.2±0.4	6.5±0.4	1.9±0.2	2.0±0.2
Doxorubicin	0.43±0.07	0.49±0.11	0.68±0.07	0.61±0.09

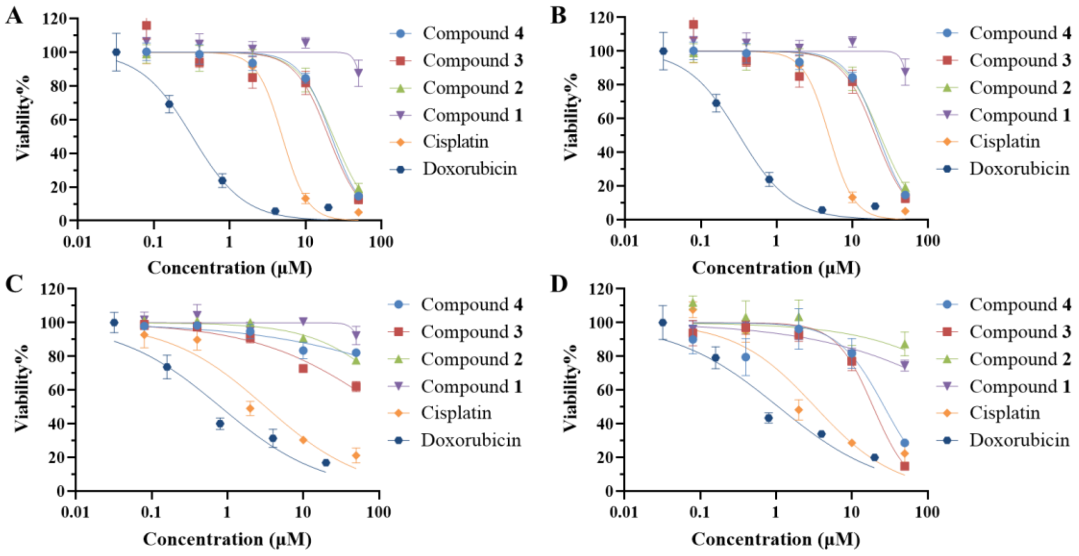

Figure S18. Viability curves of A549 and HeLa cells treated with compounds **4** to **1**, as well as cisplatin and doxorubicin, with a total treatment time of 72 h. (**A**) A549 in the dark; (**B**) A549 with irradiation; (**C**) HeLa in the dark; (**D**) HeLa with irradiation.

2.8.4 Colocalization

A549 cells were seeded in an 8-well chamber slide at a density of 10,000 cells per well and incubated for 48 h. The cells were treated with 50 μM compound **3** for 6 h. The cells were washed with phenol-red-free media three times and incubated with 5 μg/mL of the markers (Mito-tracker deep red; Lyso-tracker deep red; CellMark plasma membrane marker deep red), respectively, for 10 min at 37°C. The cells were imaged by a laser scanning confocal microscope (blue channel: excitation at 405 nm, emission at 430 to 530 nm; red channel: excitation at 637 nm, emission at 660 to 730 nm).

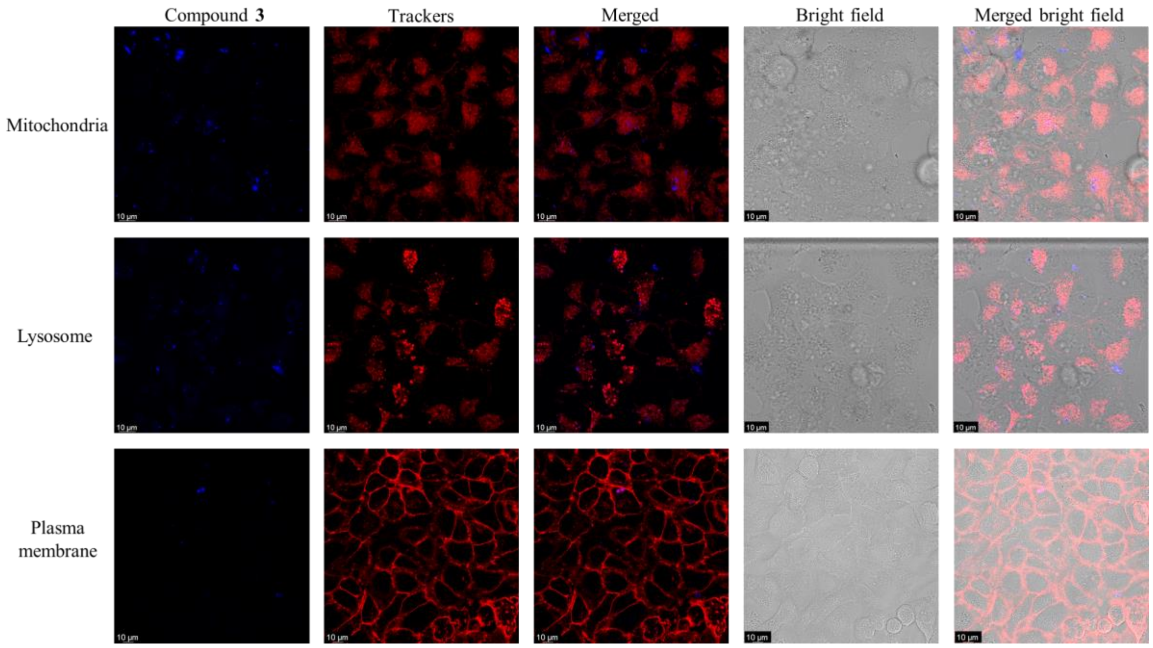

Figure S19. Co-localization of the trackers (mitochondria, lysosome, and plasma membrane) and compound **3** in A549 cells.

2.8.5 Cell imaging

A549 cells were seeded in a 6-well plate at a density of 100,000 cells per well and incubated for 72 h. The cells were treated with 50 µM compound **3** and 20 µM doxorubicin for 6 h. The compound-containing media were replaced with a phenol-red-free medium. The cells were irradiated with a white LED (400-700 nm, 7.6 mW/cm²) for 1 h and then incubated for another 2 h. The cells were imaged by an optical microscope.

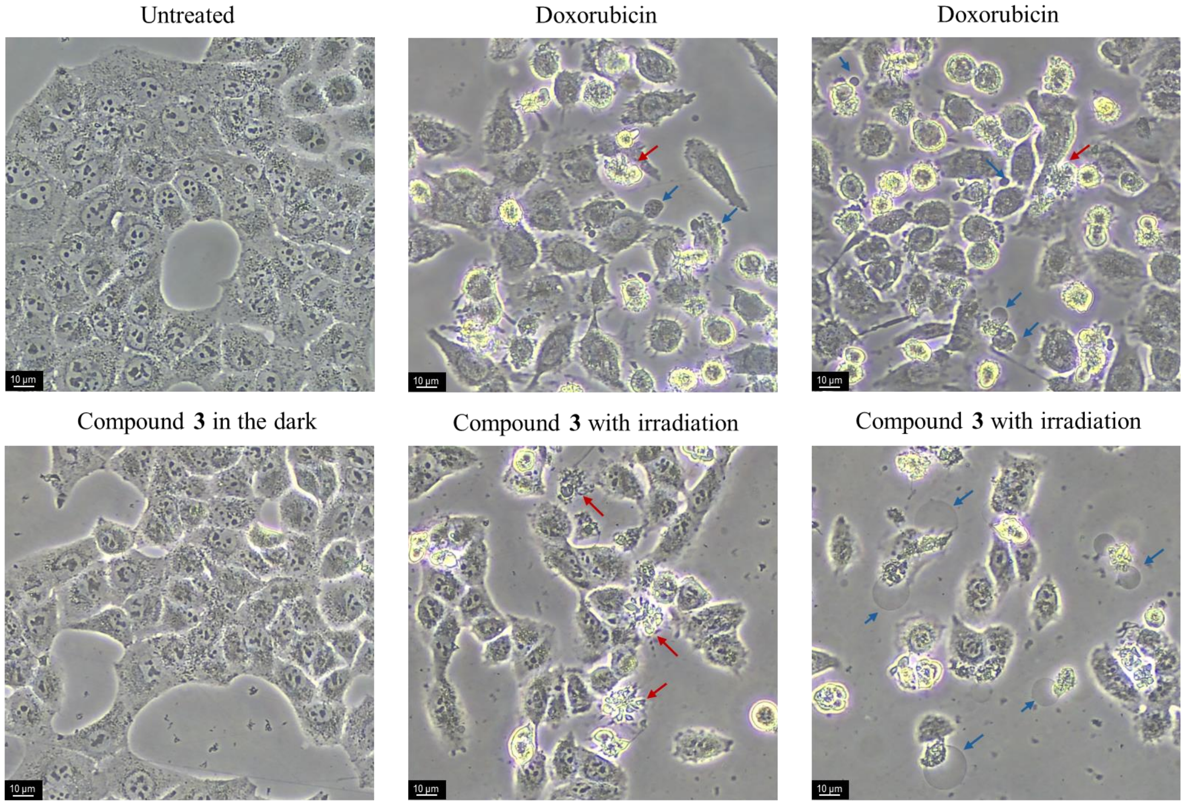

Figure S20. Morphologies of the A549 cells treated with compound **3** and doxorubicin. Compound **3** induces cell apoptosis (red arrow) and necrosis (blue arrow).

2.8.6 PI/Annexin V double staining

A549 cells were seeded in a 6-well plate at a density of 100,000 cells per well and incubated for 48 h. The cells were treated with 50 μM compound **3** for 6 h. The compound-containing media were replaced with a phenol-red-free medium. The cells were irradiated with a white LED (400-700 nm, 7.6 mW/cm²) for 1 h and then incubated for another 17 h. The cells treated with 20 μM doxorubicin for 6 h were included as a positive control. Afterward, the cells were washed with PBS twice and harvested by trypsinization, and the washing solutions were also collected. The cell suspensions were spun at 600 g for 3 min, and the cell pellets were washed twice with Annexin-binding buffer (10 mM HEPES, 140 mM NaCl, and 2.5 mM $CaCl_2$, pH = 7.4). Then the cells were double-stained with Annexin V-633 and propidium iodide at r.t. for 15 min. The cell suspensions were then analyzed by a flow cytometer.

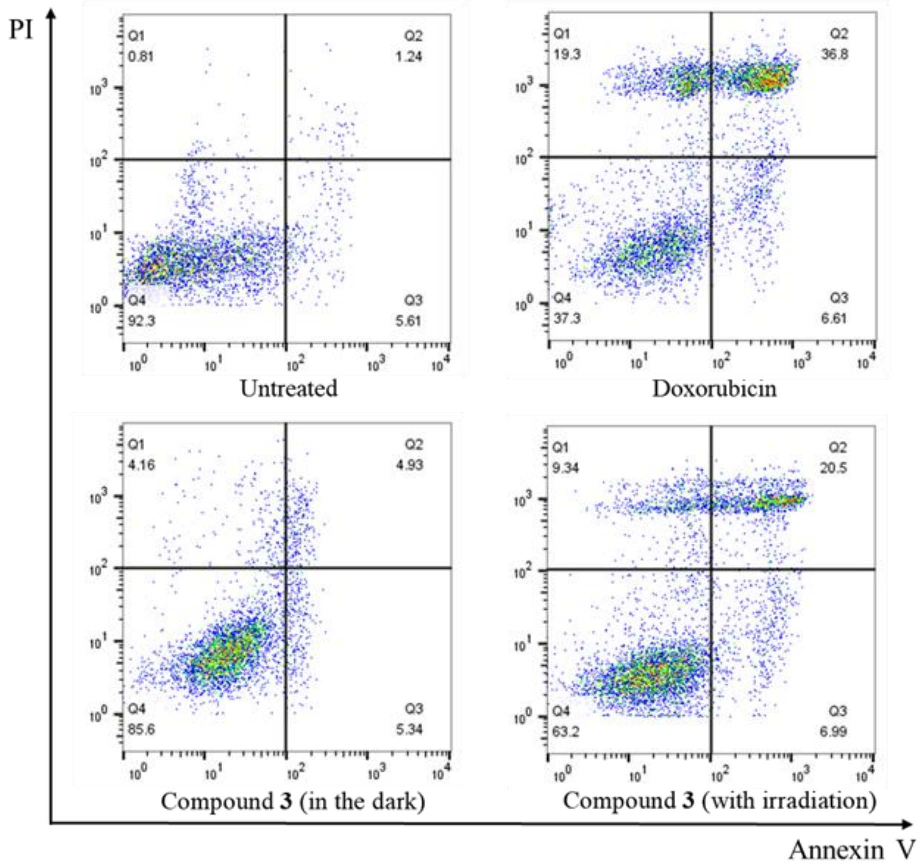

Figure S21. PI/Annexin V-633 double staining of A549 cells treated with compound **3** and doxorubicin.

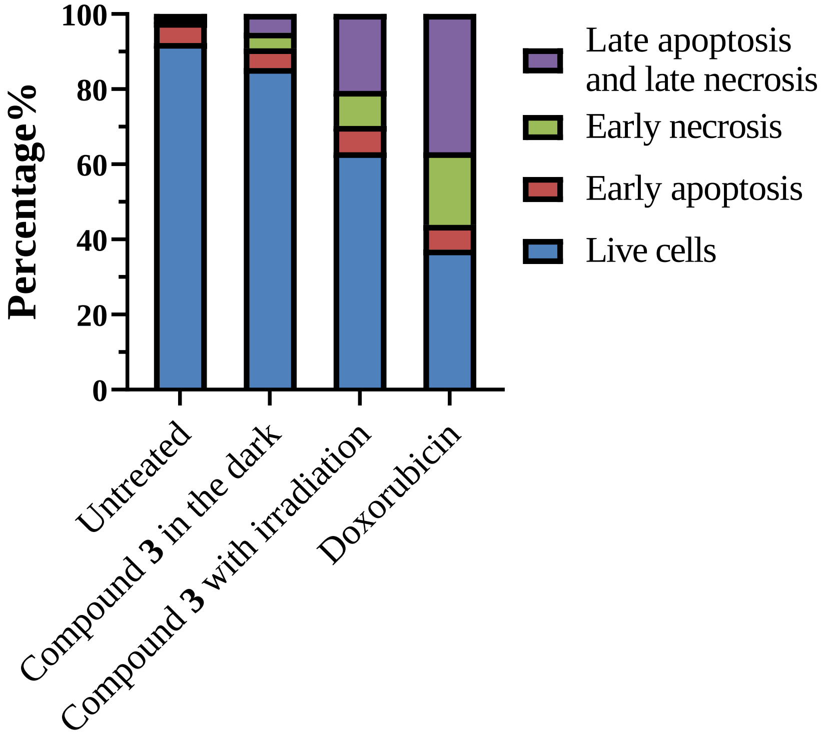

Figure S22. Analysis of cell death modes of A549 cells treated with compound **3** and doxorubicin.

3. Copies of Spectrums

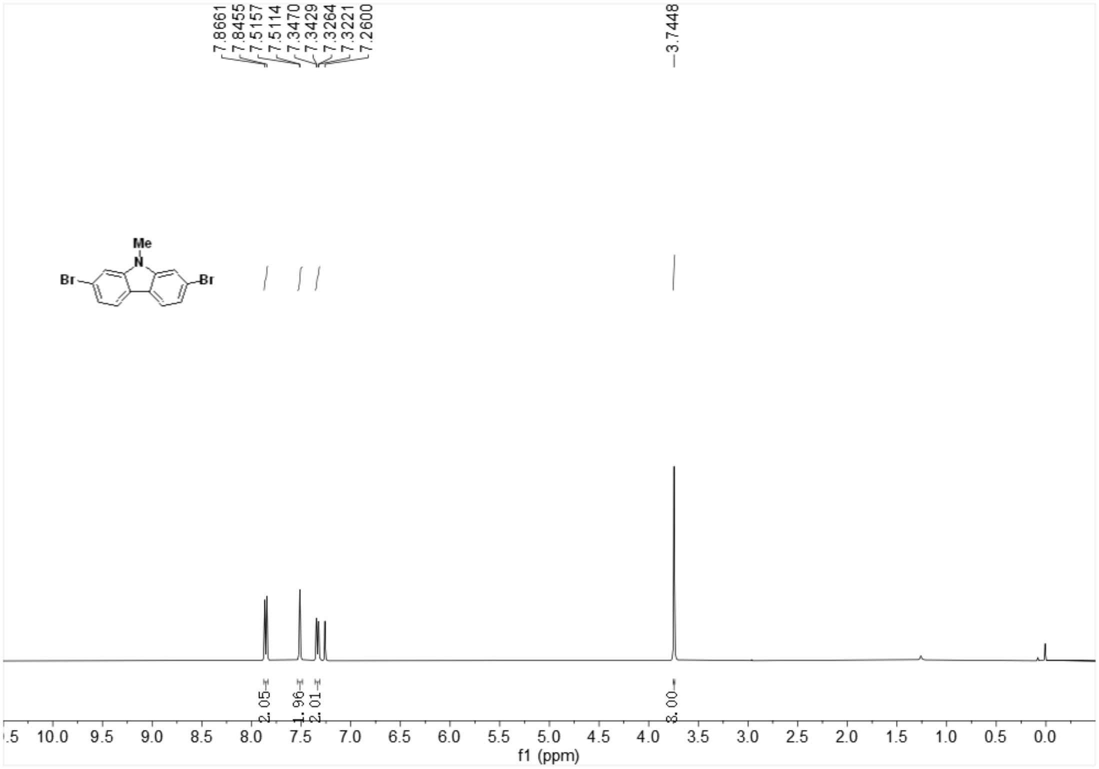

Figure S23. ^{1}H NMR spectrum of compound 2,7-dibromo-9-methyl-9*H*-carbazole ($CDCl_3$, 25 ^{o}C, 400 MHz).

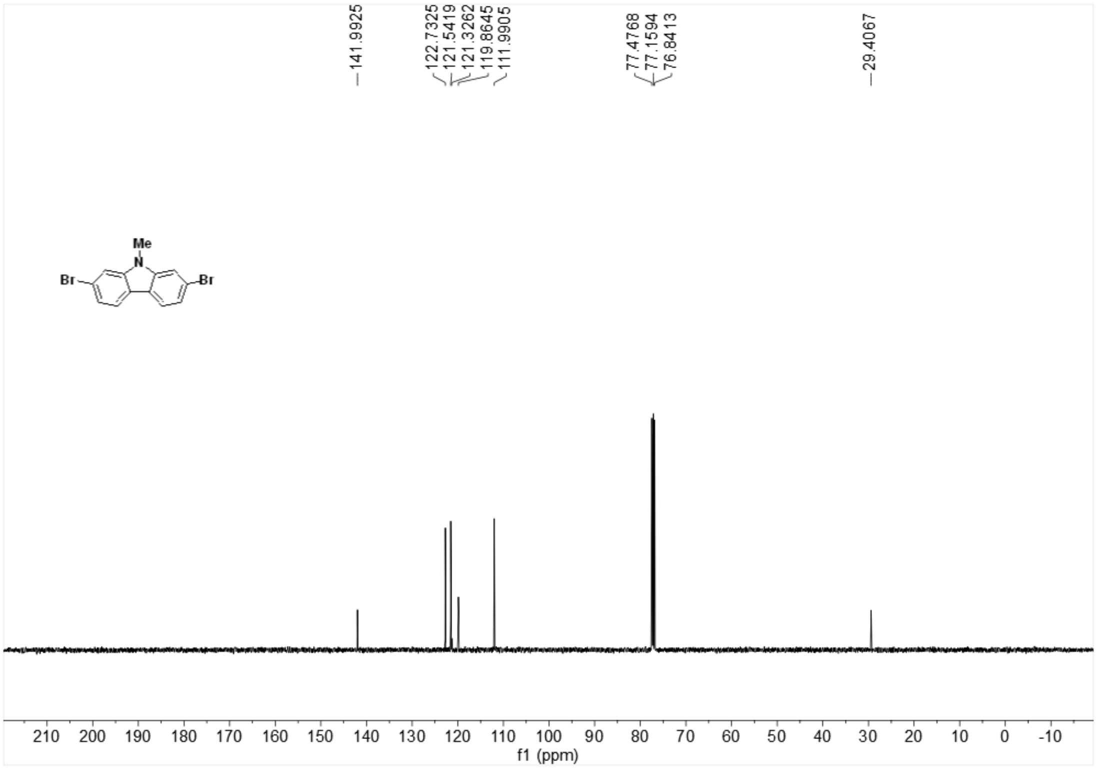

Figure S24. ^{13}C NMR spectrum of compound 2,7-dibromo-9-methyl-9*H*-carbazole ($CDCl_3$, 25 ^{o}C, 101 MHz).

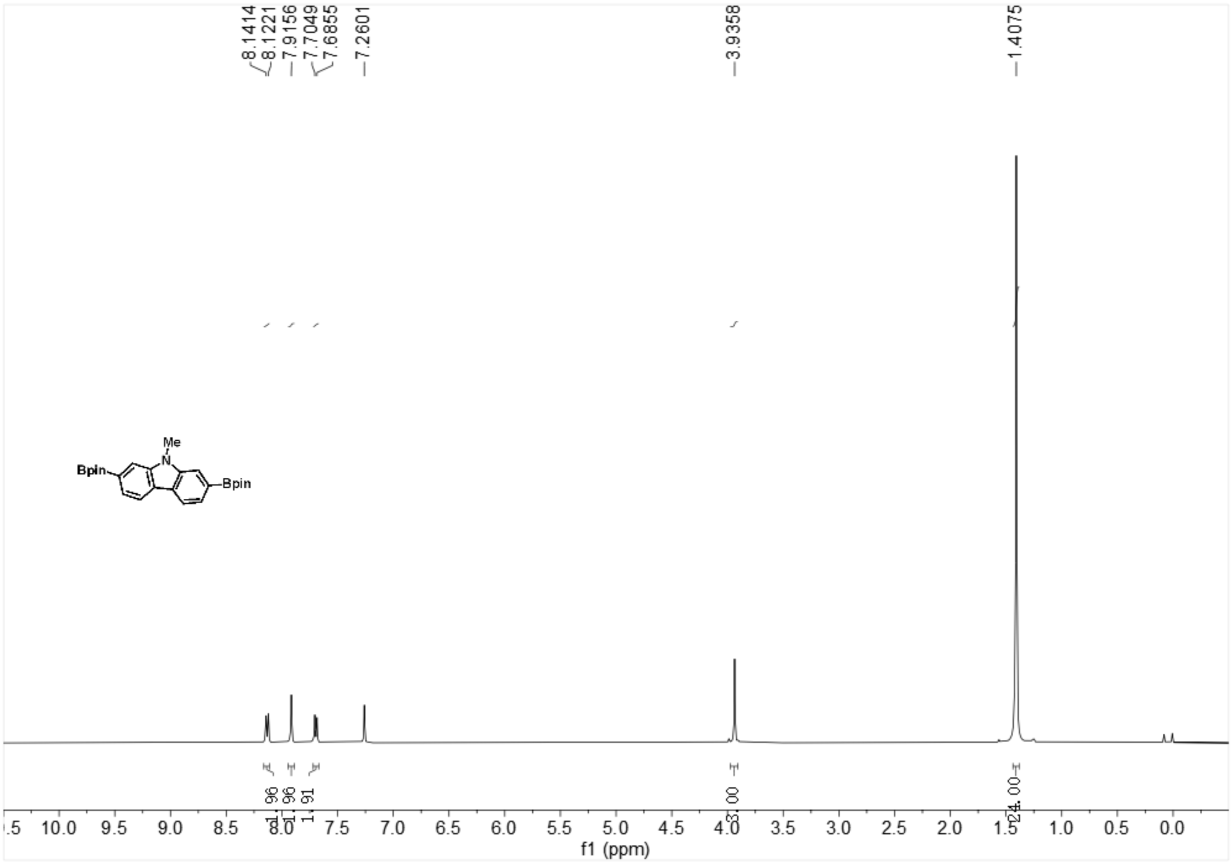

Figure S25. ^{1}H NMR spectrum of compound 9-methyl-2,7-bis(4,4,5,5-tetramethyl-1,3,2-dioxaborolan-2-yl)-9*H*-carbazole ($CDCl_3$, 25 °C, 400 MHz).

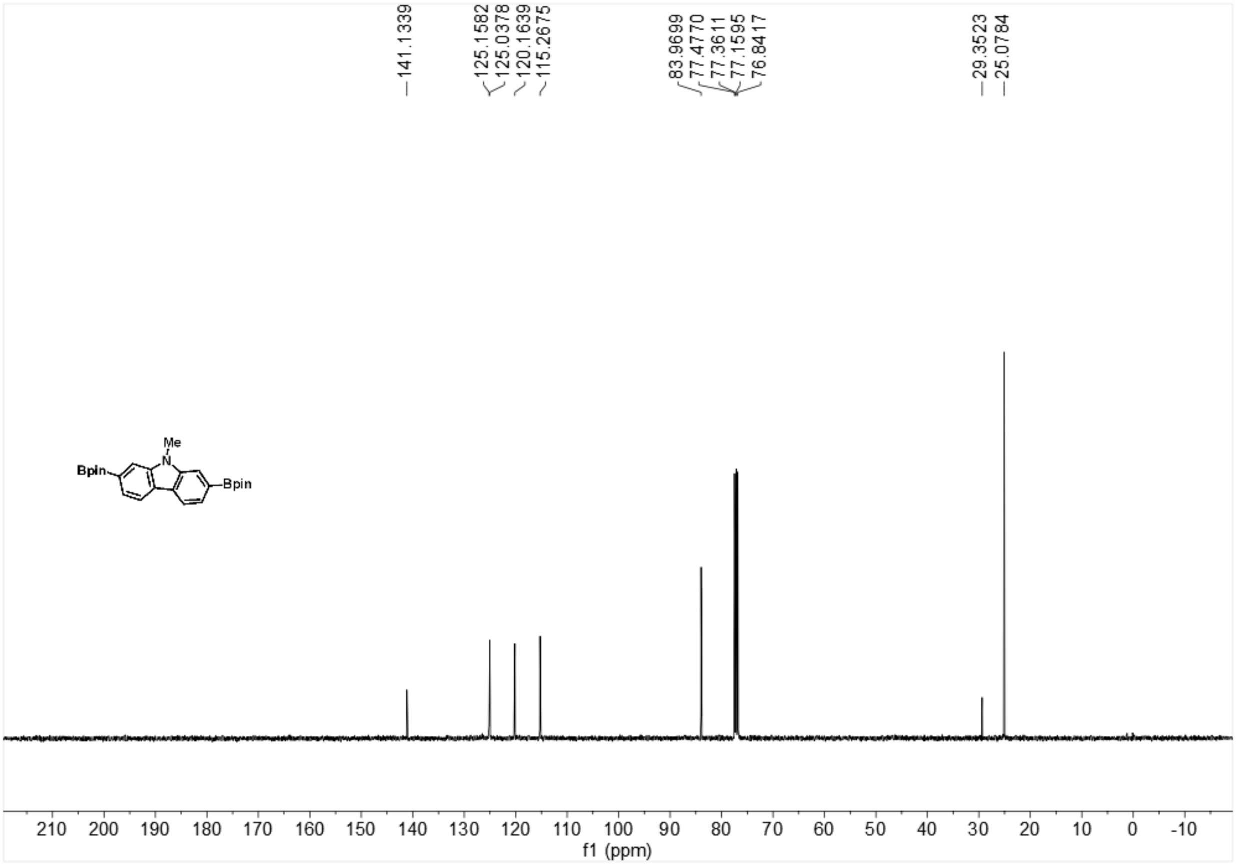

Figure S26. ^{13}C NMR spectrum of compound 9-methyl-2,7-bis(4,4,5,5-tetramethyl-1,3,2-dioxaborolan-2-yl)-9*H*-carbazole ($CDCl_3$, 25 °C, 101 MHz).

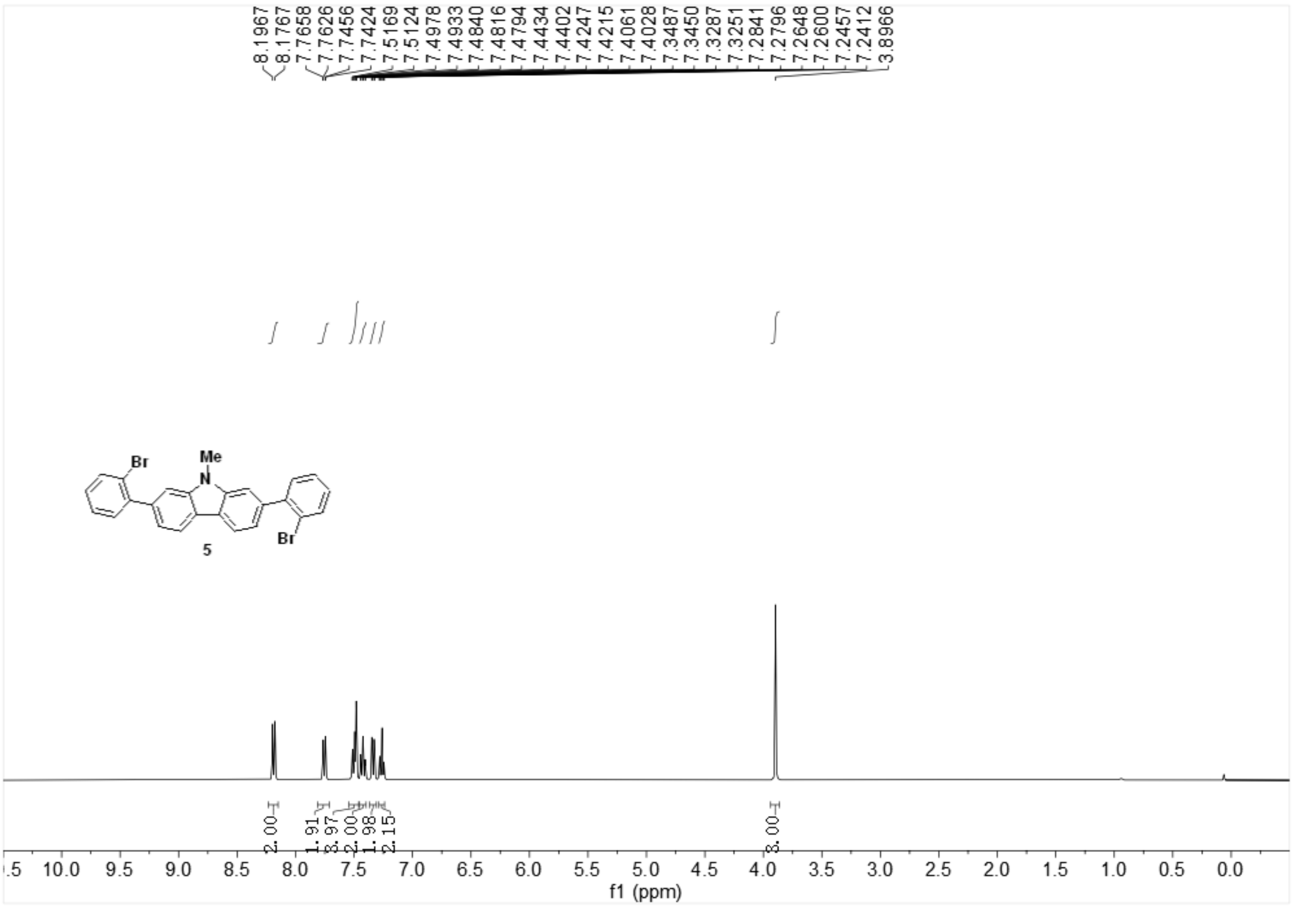

Figure S27. ^{1}H NMR spectrum of compound **5** ($CDCl_3$, 25 °C, 400 MHz).

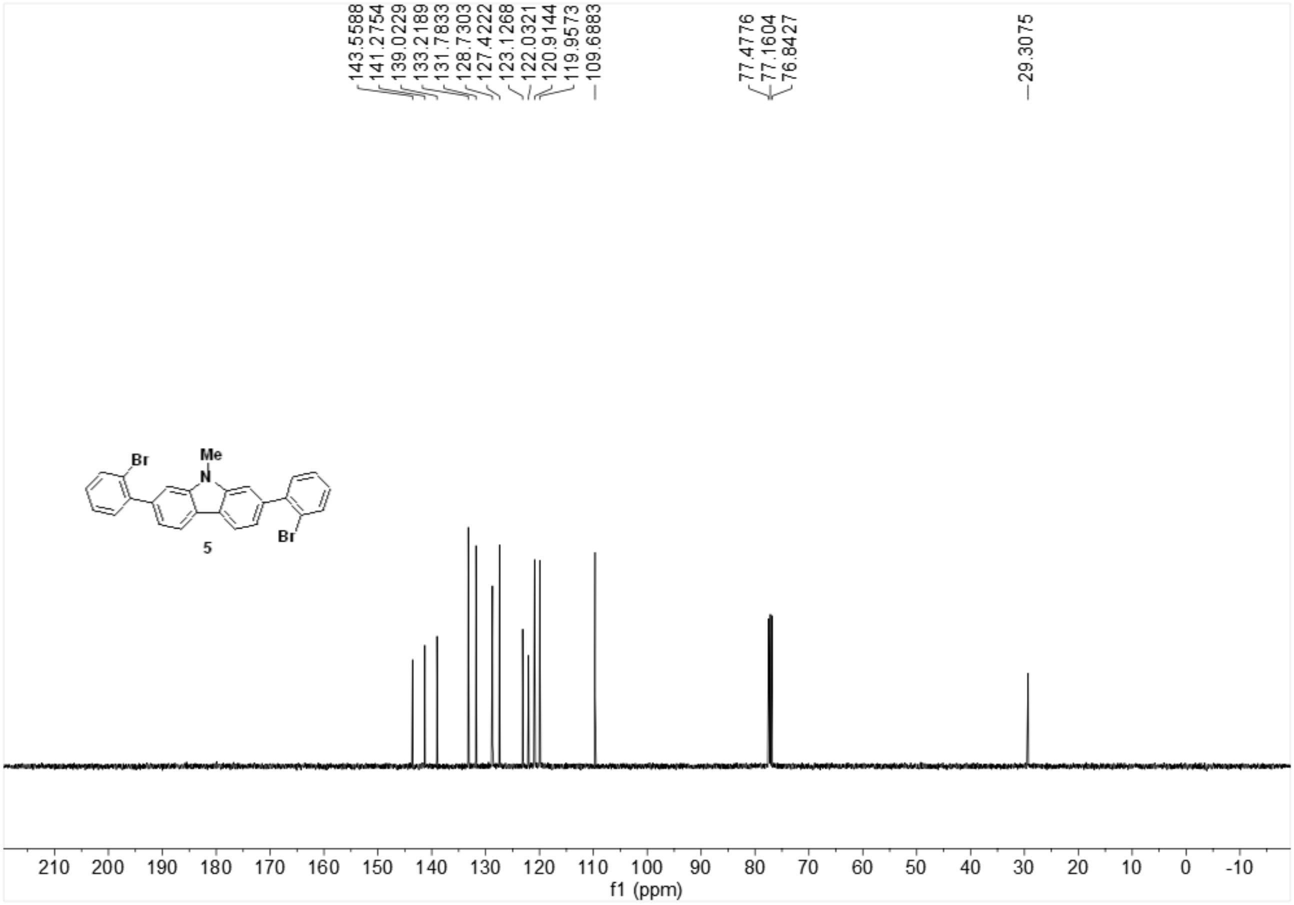

Figure S28. ^{13}C NMR spectrum of compound **5** ($CDCl_3$, 25 °C, 101 MHz).

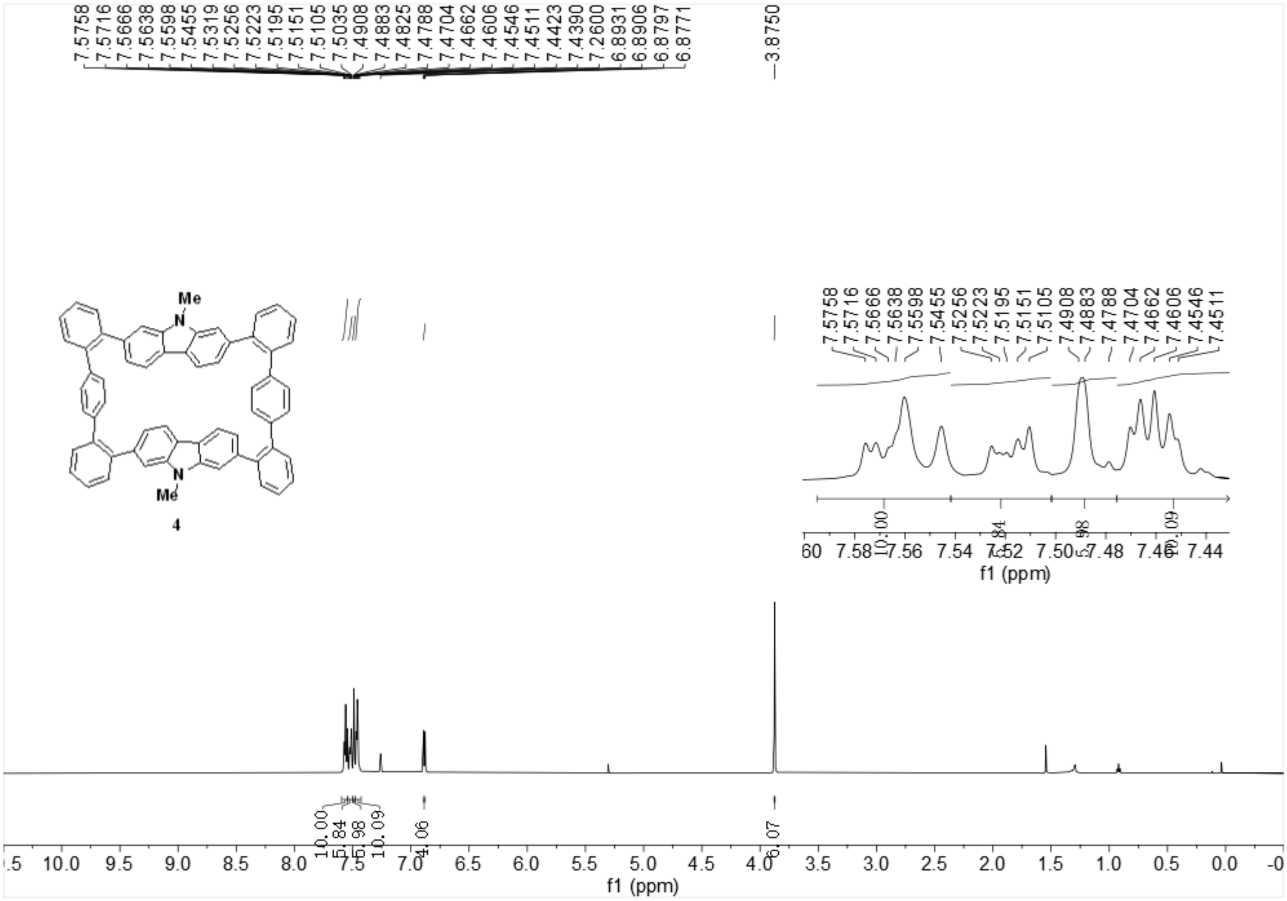

Figure S29. ^{1}H NMR spectrum of compound **4** ($CDCl_3$, 25 °C, 400 MHz).

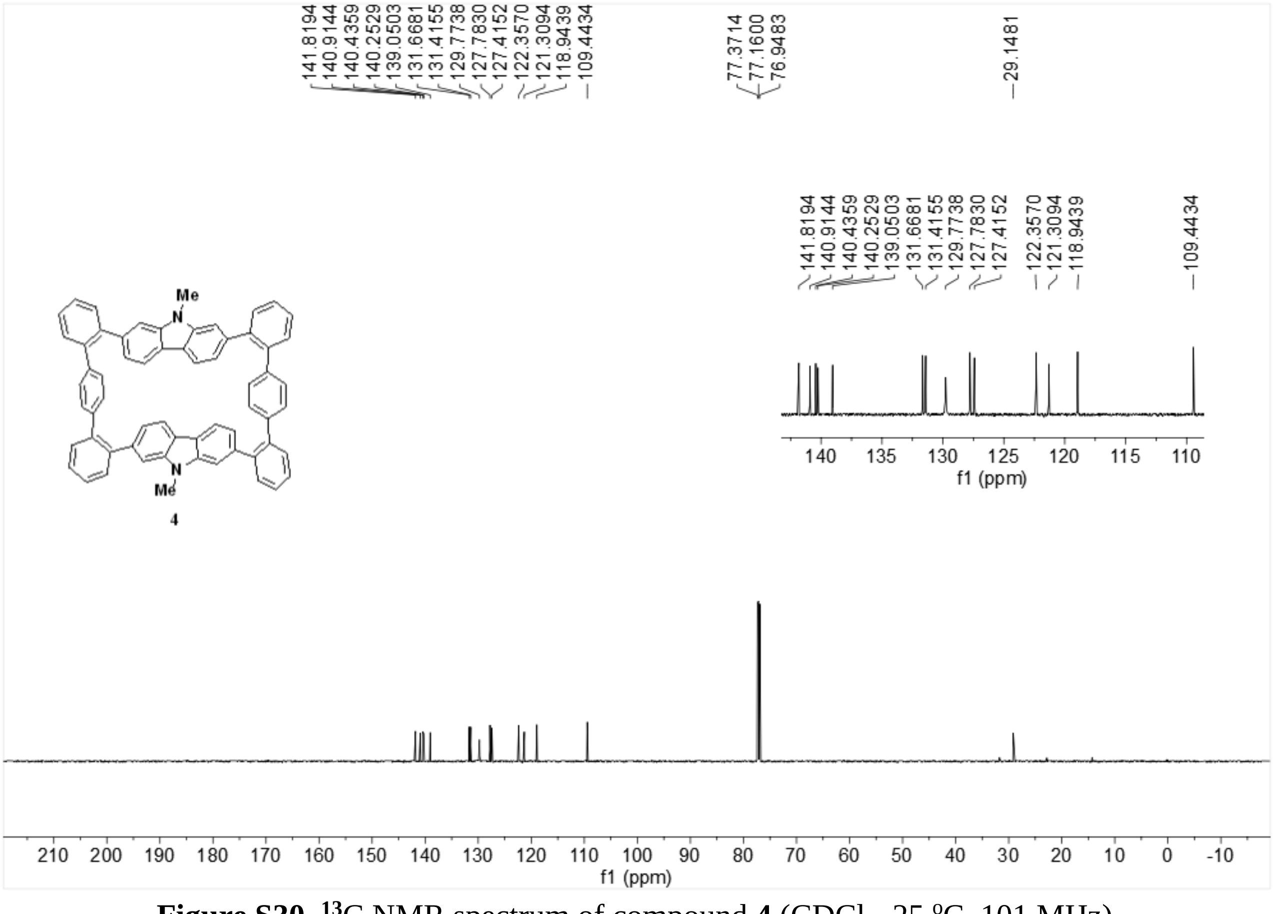

Figure S30. ^{13}C NMR spectrum of compound **4** ($CDCl_3$, 25 °C, 101 MHz).

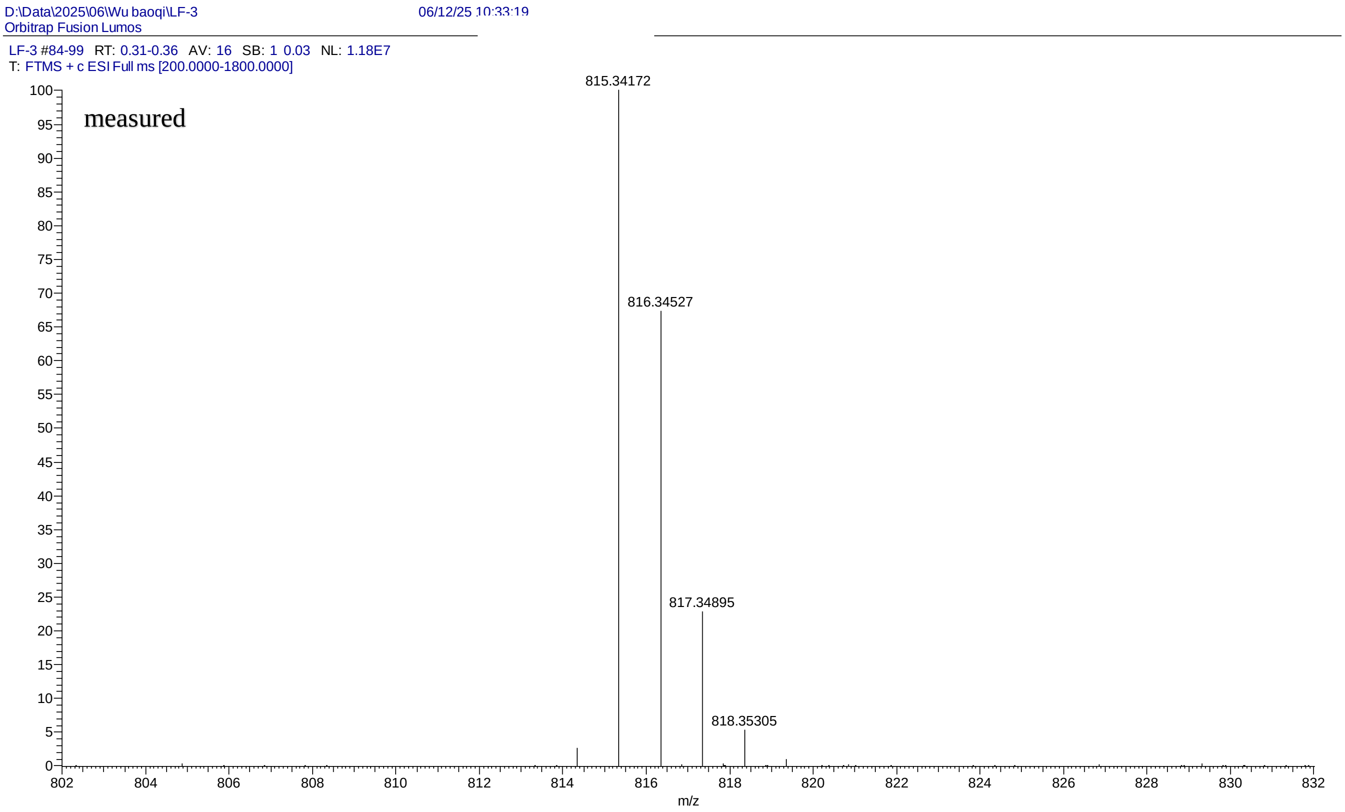

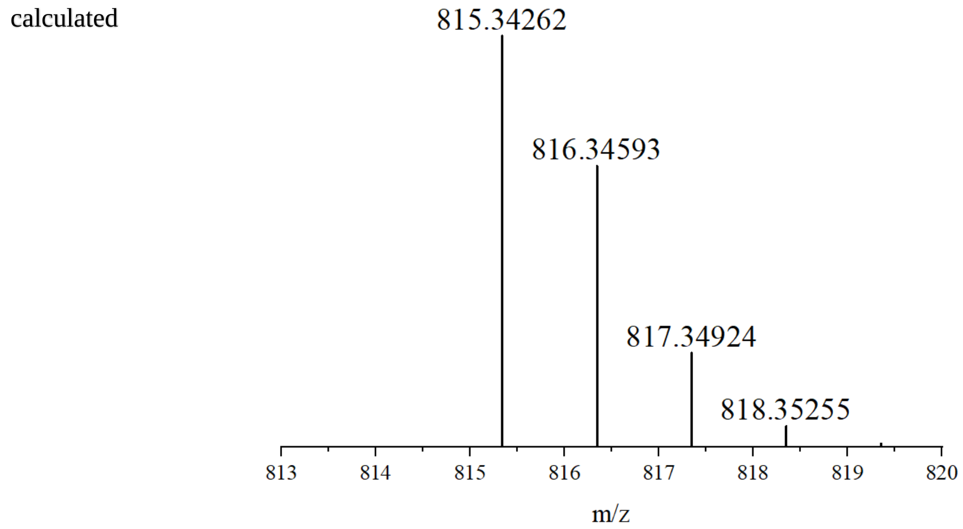

Figure S31. MALDI-TOF mass spectrums of **4**.

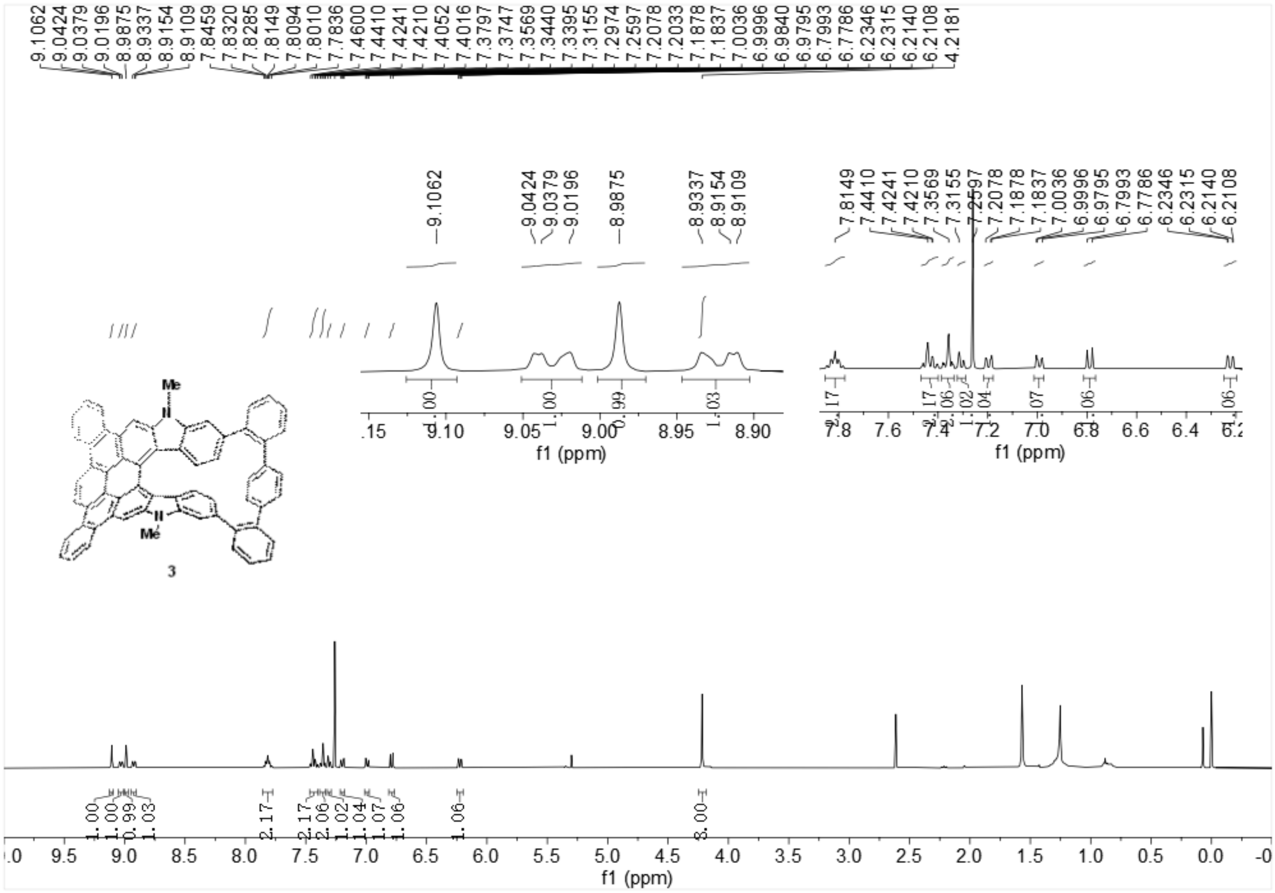

Figure S32. ^{1}H NMR spectrum of compound **3** ($CDCl_3$, 25 °C, 400 MHz).

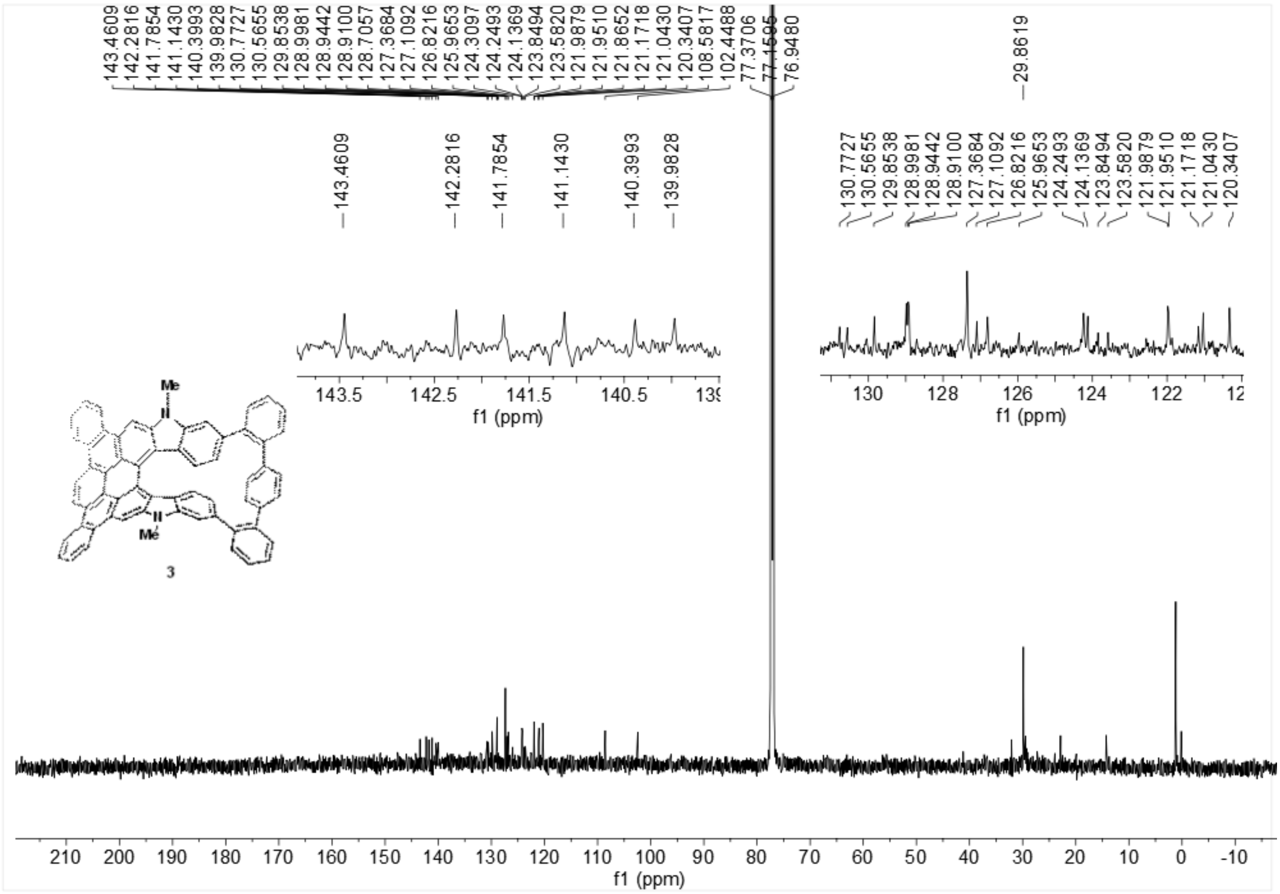

Figure S33. ^{13}C NMR spectrum of compound **3** ($CDCl_3$, 25 °C, 101 MHz).

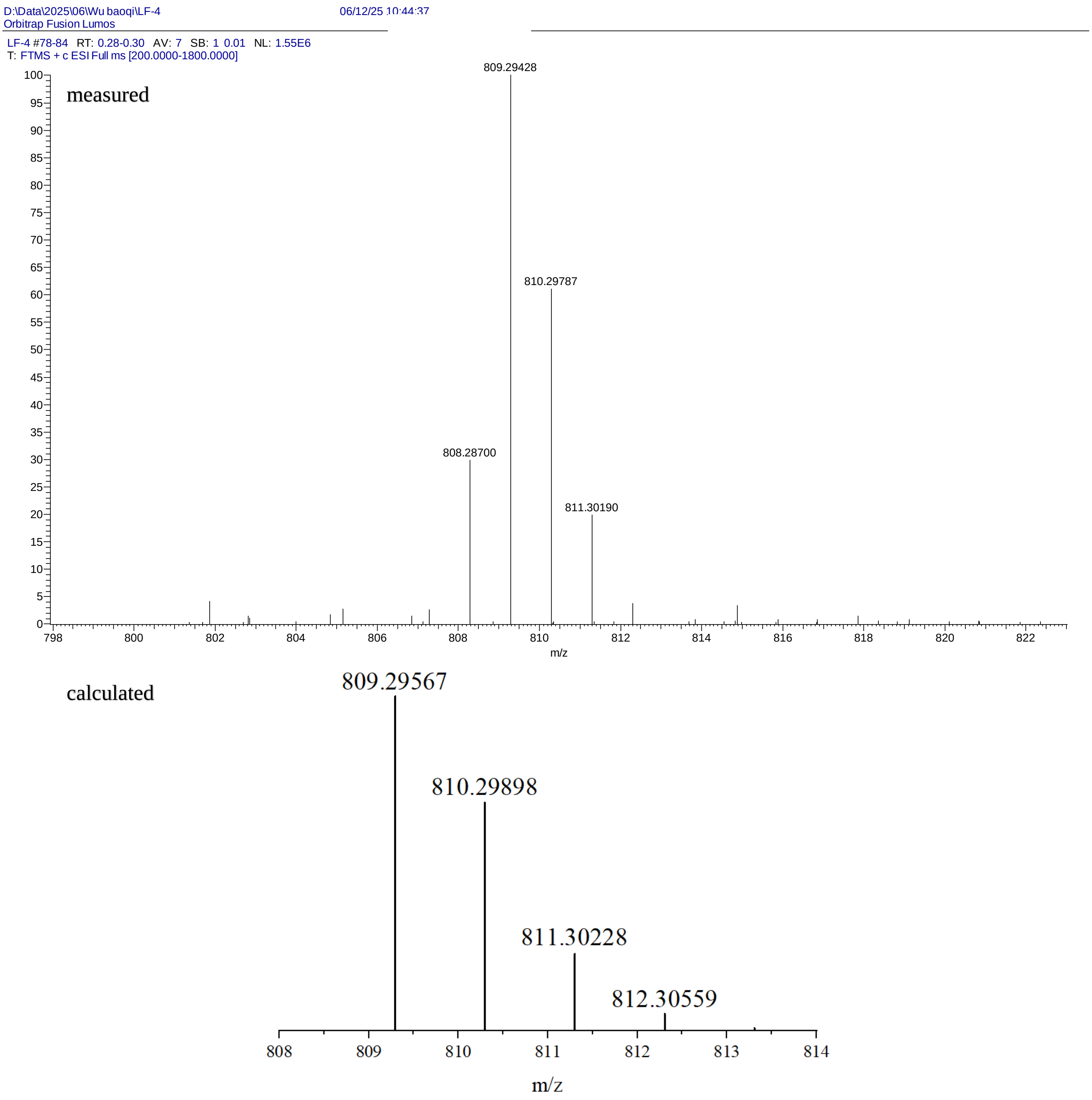

Figure S34. MALDI-TOF mass spectrums of **3**.

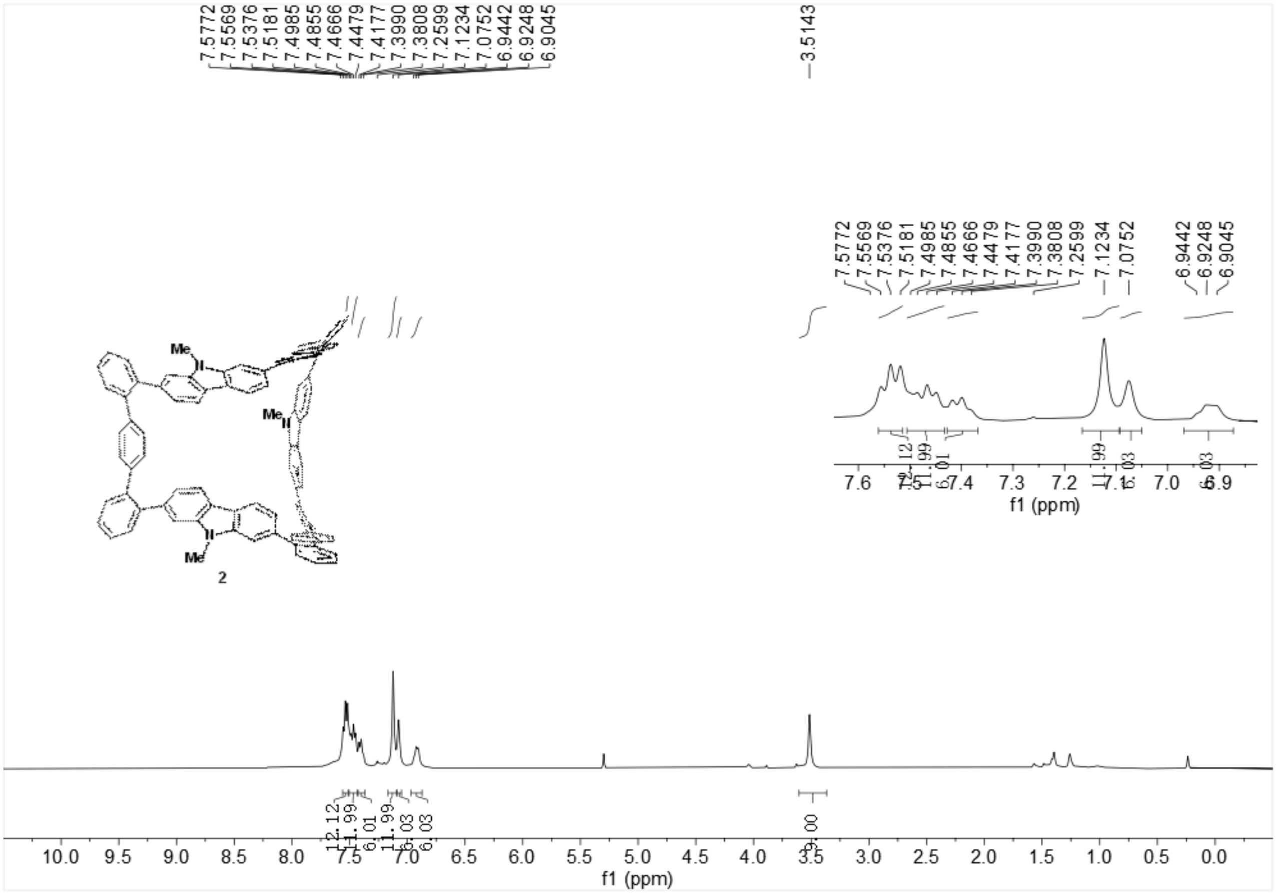

Figure S35. ^{1}H NMR spectrum of compound **2** ($CDCl_3$, 25 °C, 400 MHz).

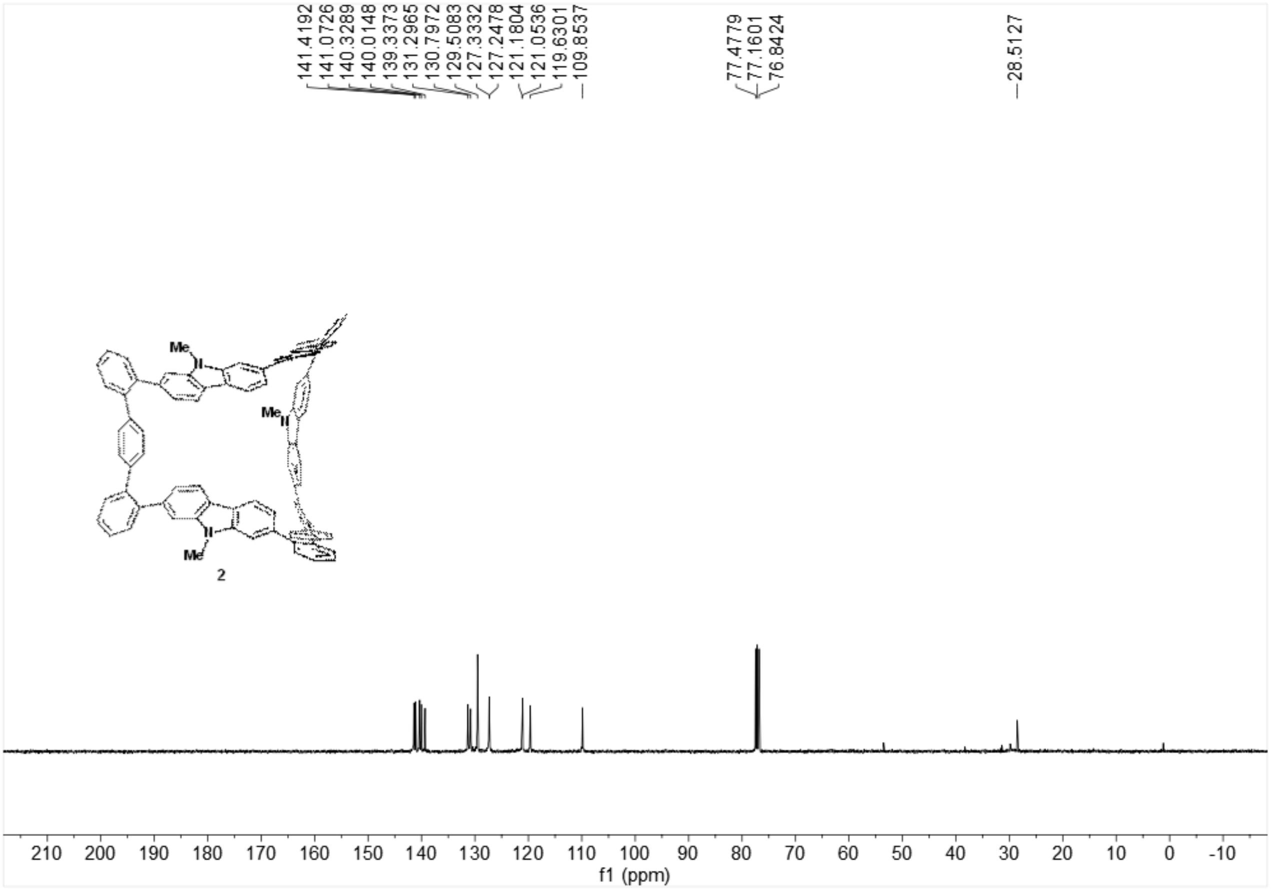

Figure S36. ^{13}C NMR spectrum of compound **2** ($CDCl_3$, 25 °C, 101 MHz).

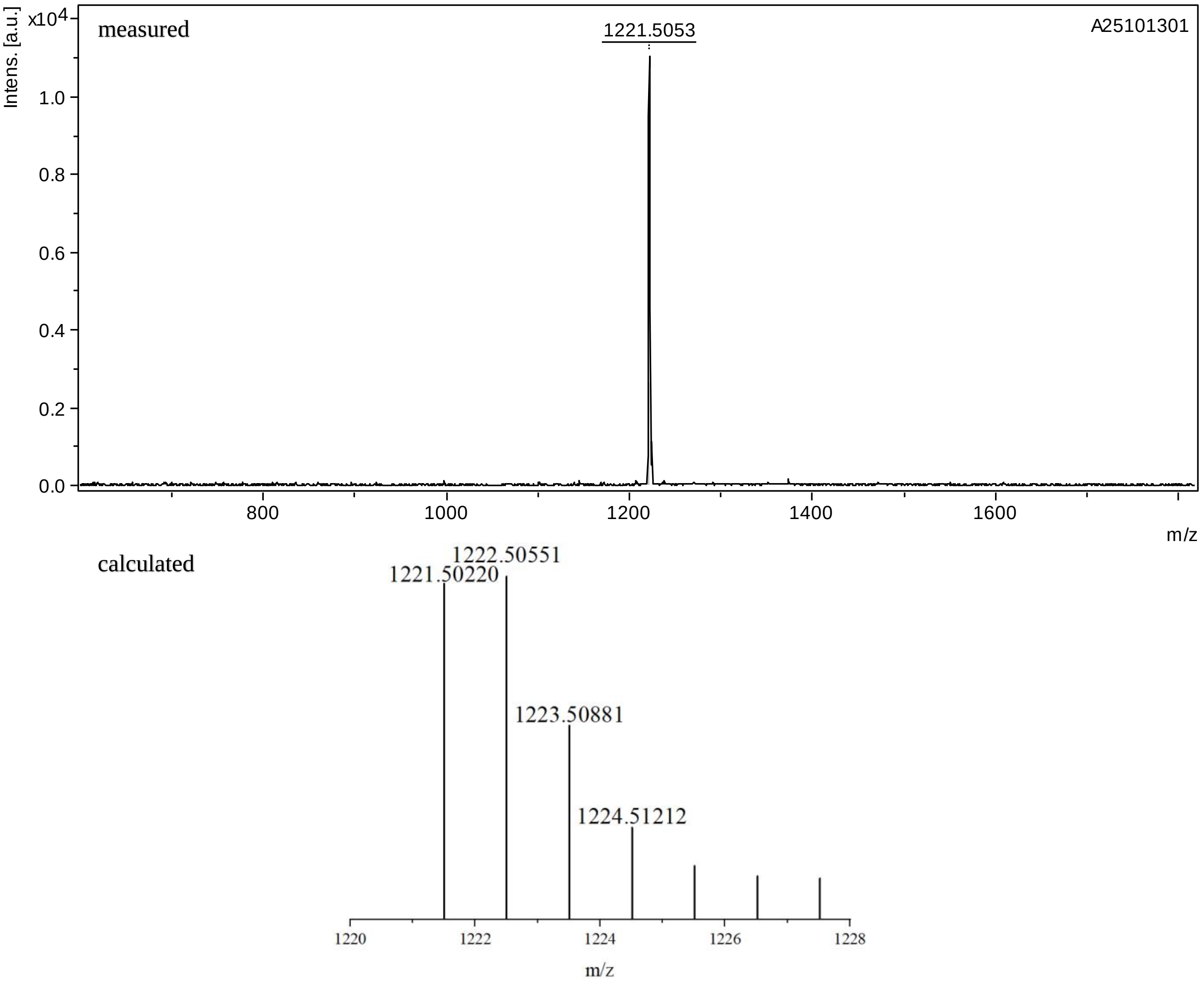

Figure S37. MALDI-TOF mass spectrums of **2**.

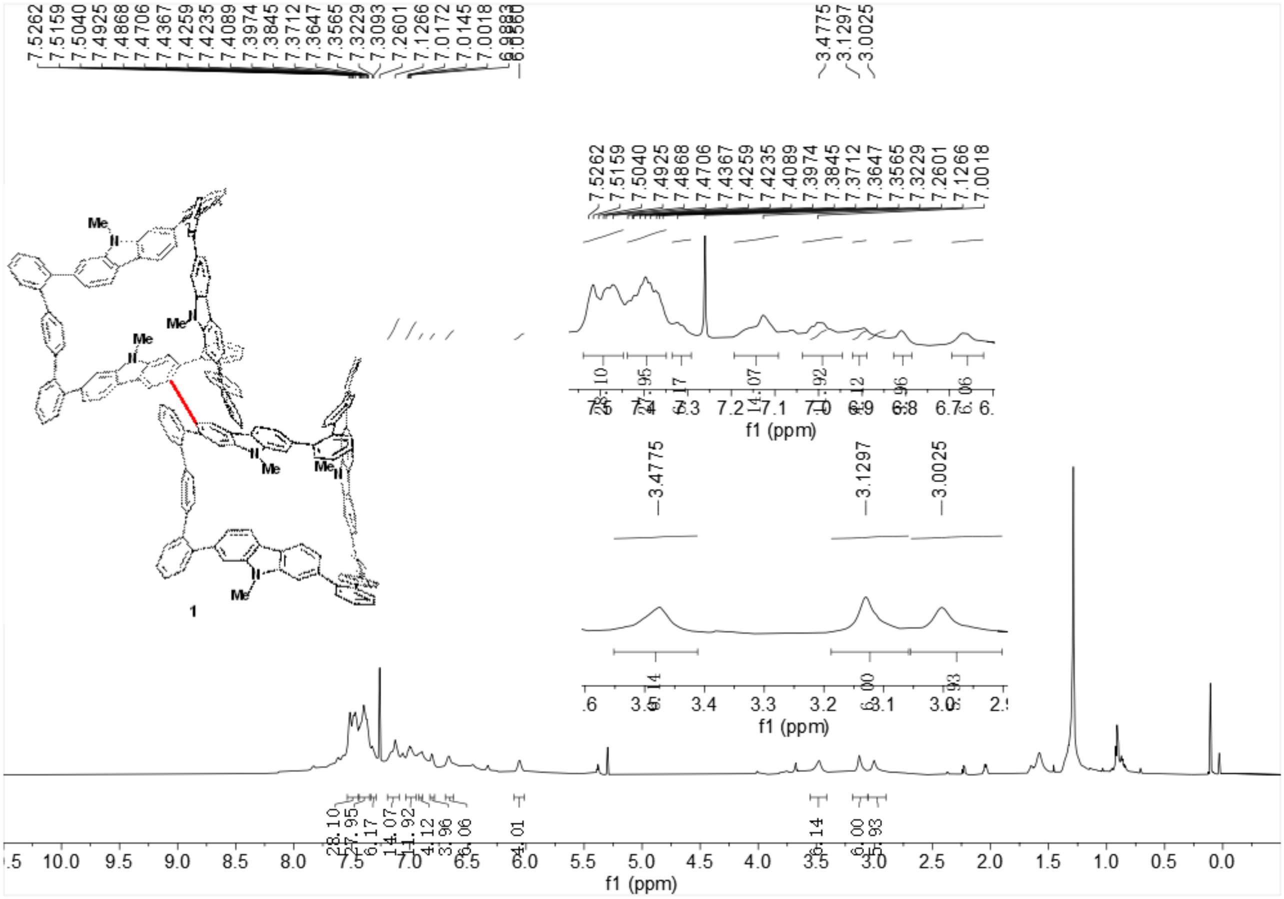

Figure S38. ^{1}H NMR spectrum of compound **1** ($CDCl_3$, 25 °C, 600 MHz).

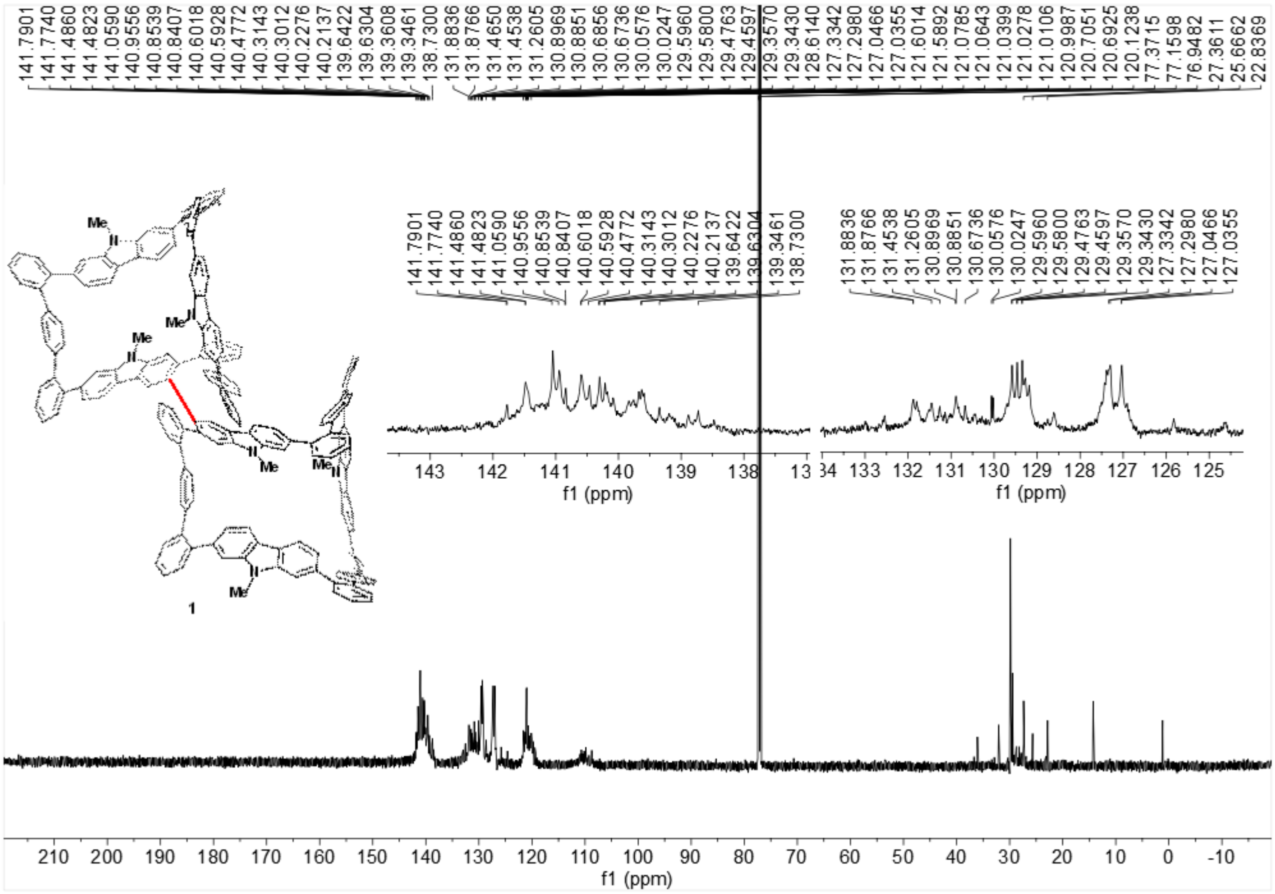

Figure S39. ^{13}C NMR spectrum of compound **1** ($CDCl_3$, 25 °C, 151 MHz).

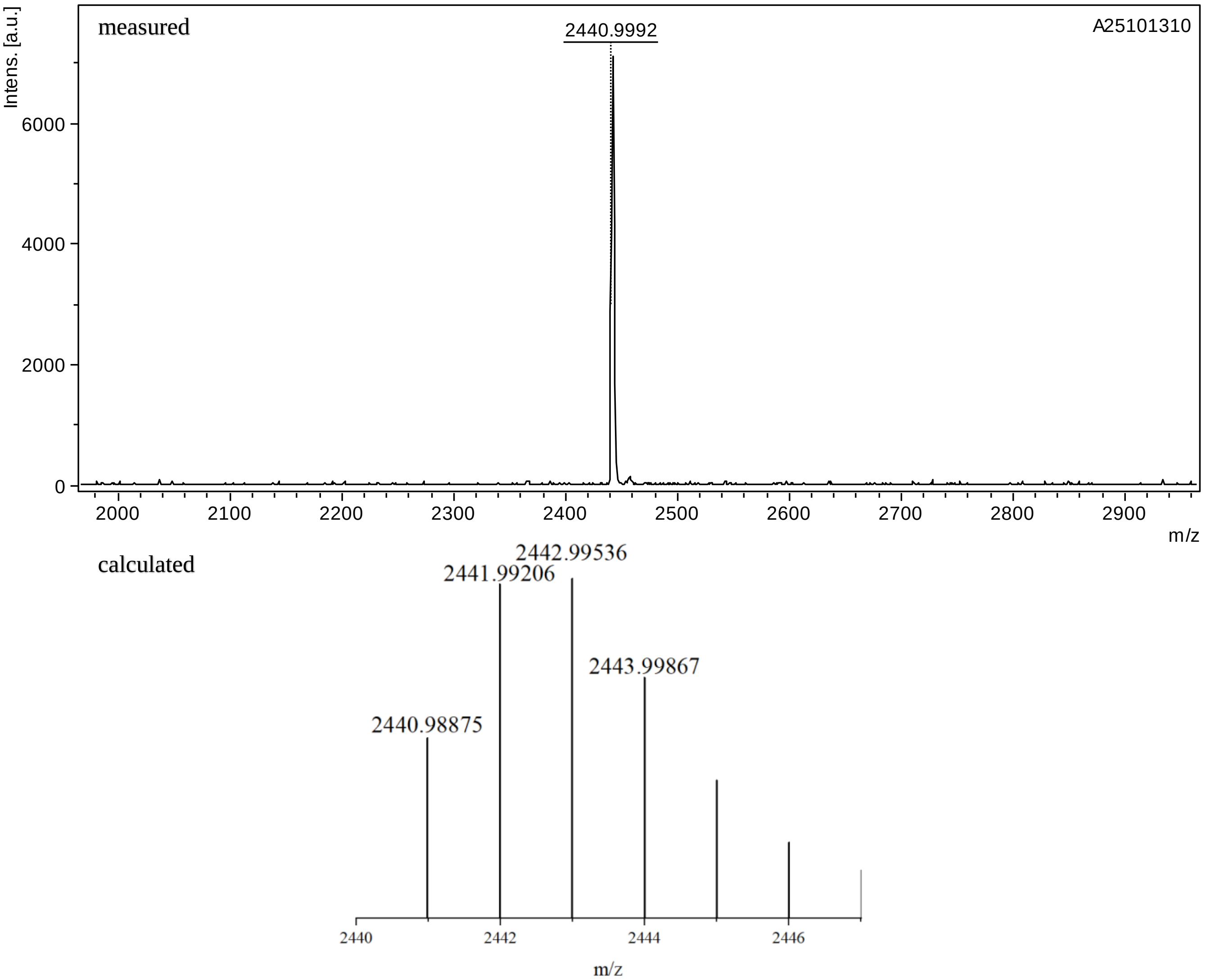

Figure S40. MALDI-TOF mass spectrums of **1**.

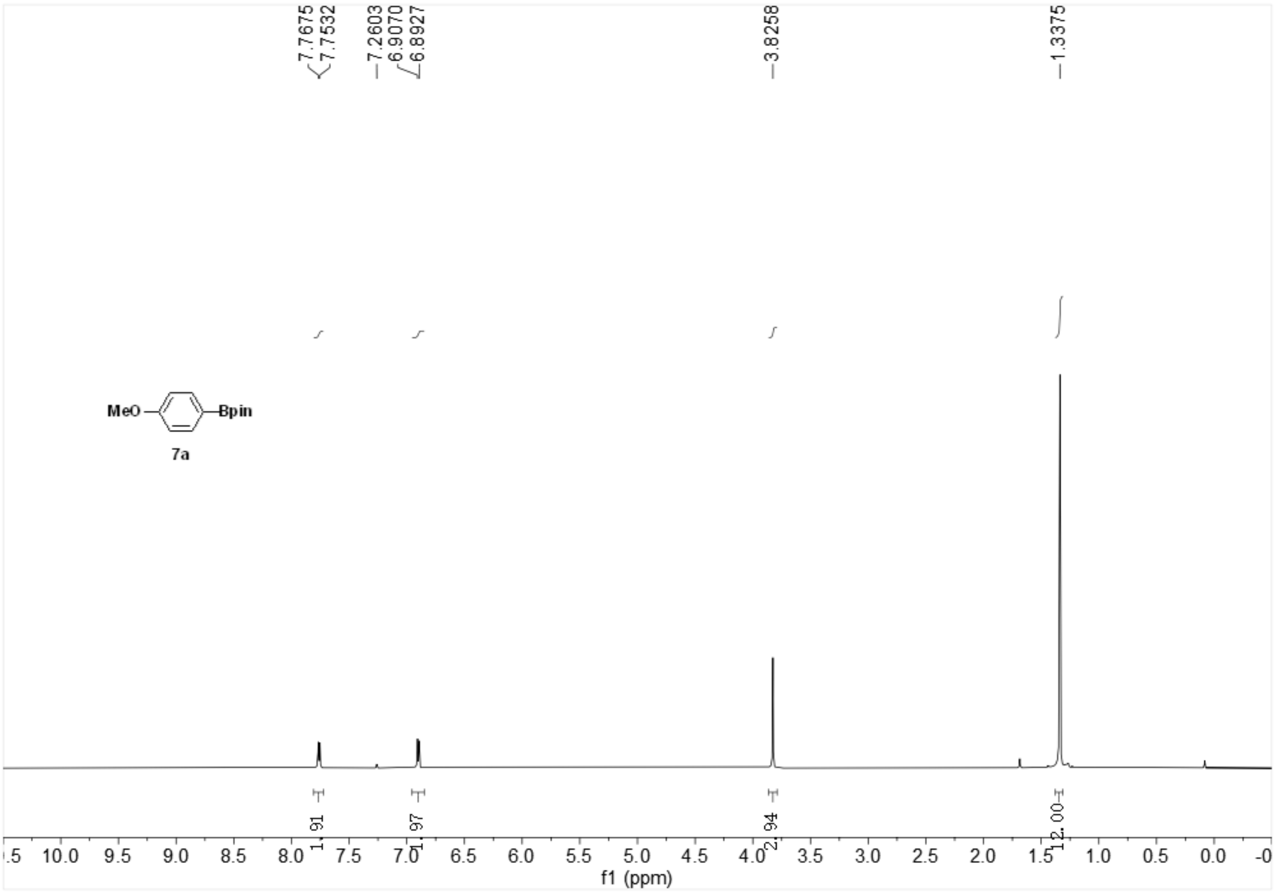

Figure S41. ^{1}H NMR spectrum of compound **7a** ($CDCl_3$, 25 °C, 600 MHz).

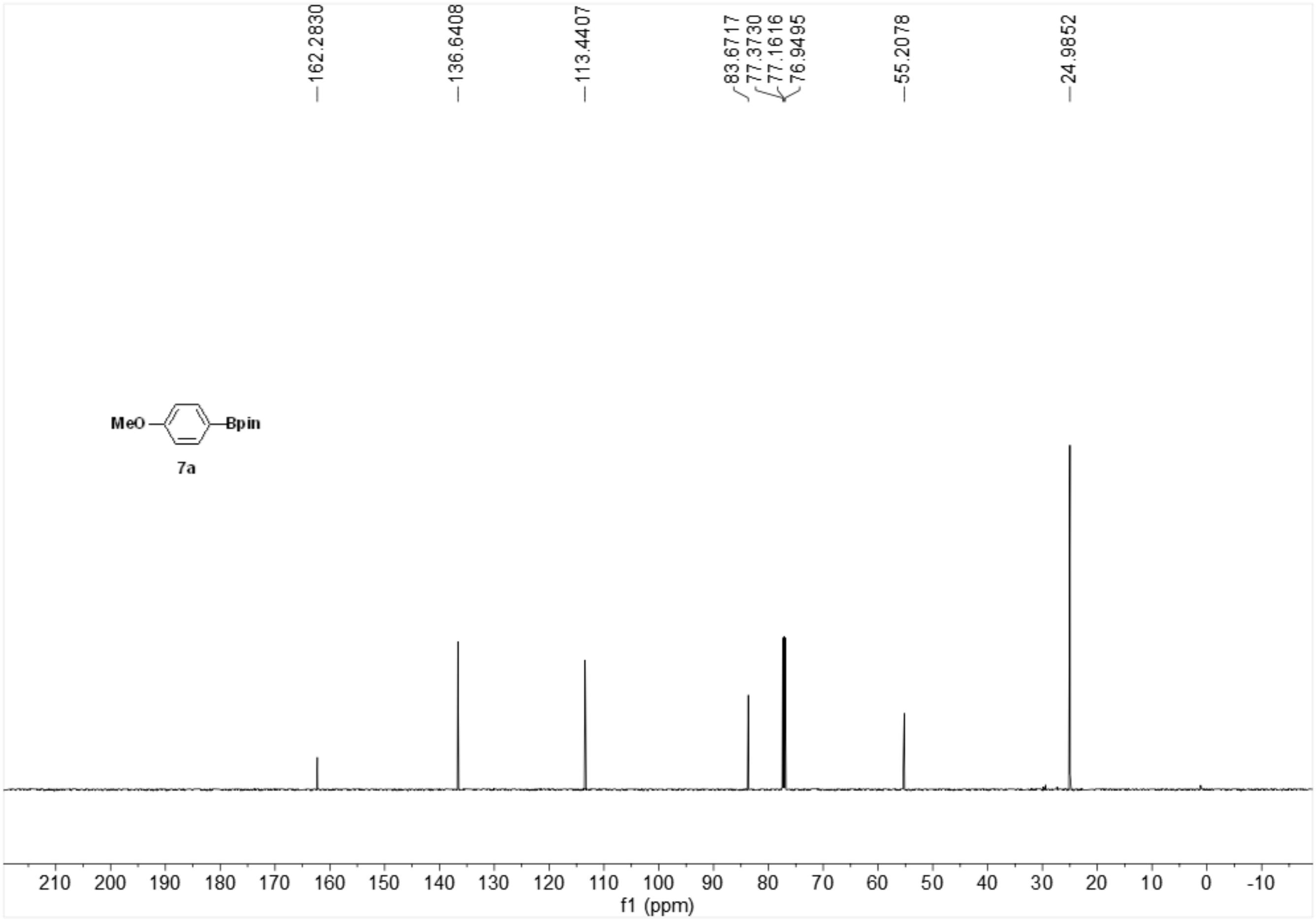

Figure S42. ^{13}C NMR spectrum of compound **7a** ($CDCl_3$, 25 °C, 151 MHz).

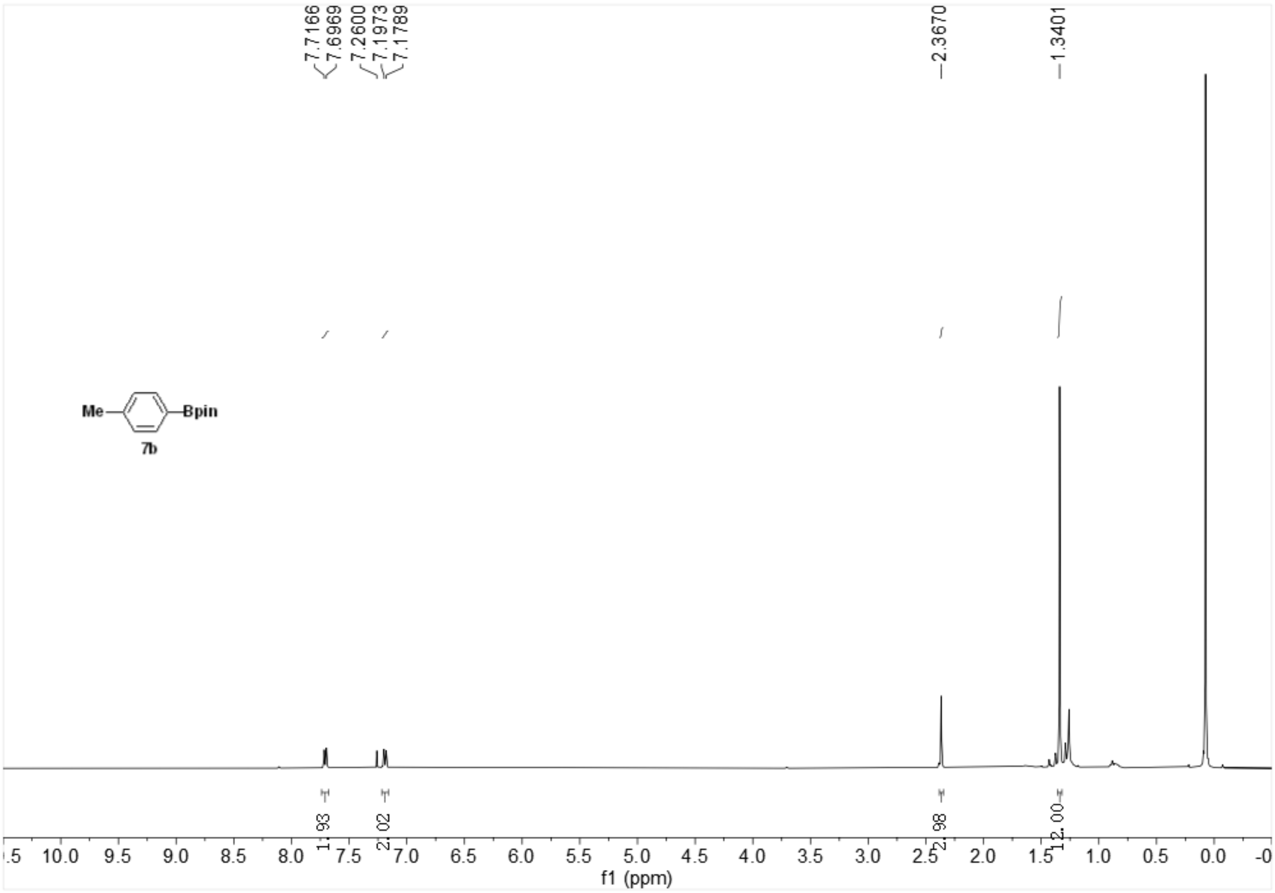

Figure S43. ^{1}H NMR spectrum of compound **7b** ($CDCl_3$, 25 ^{o}C, 400 MHz).

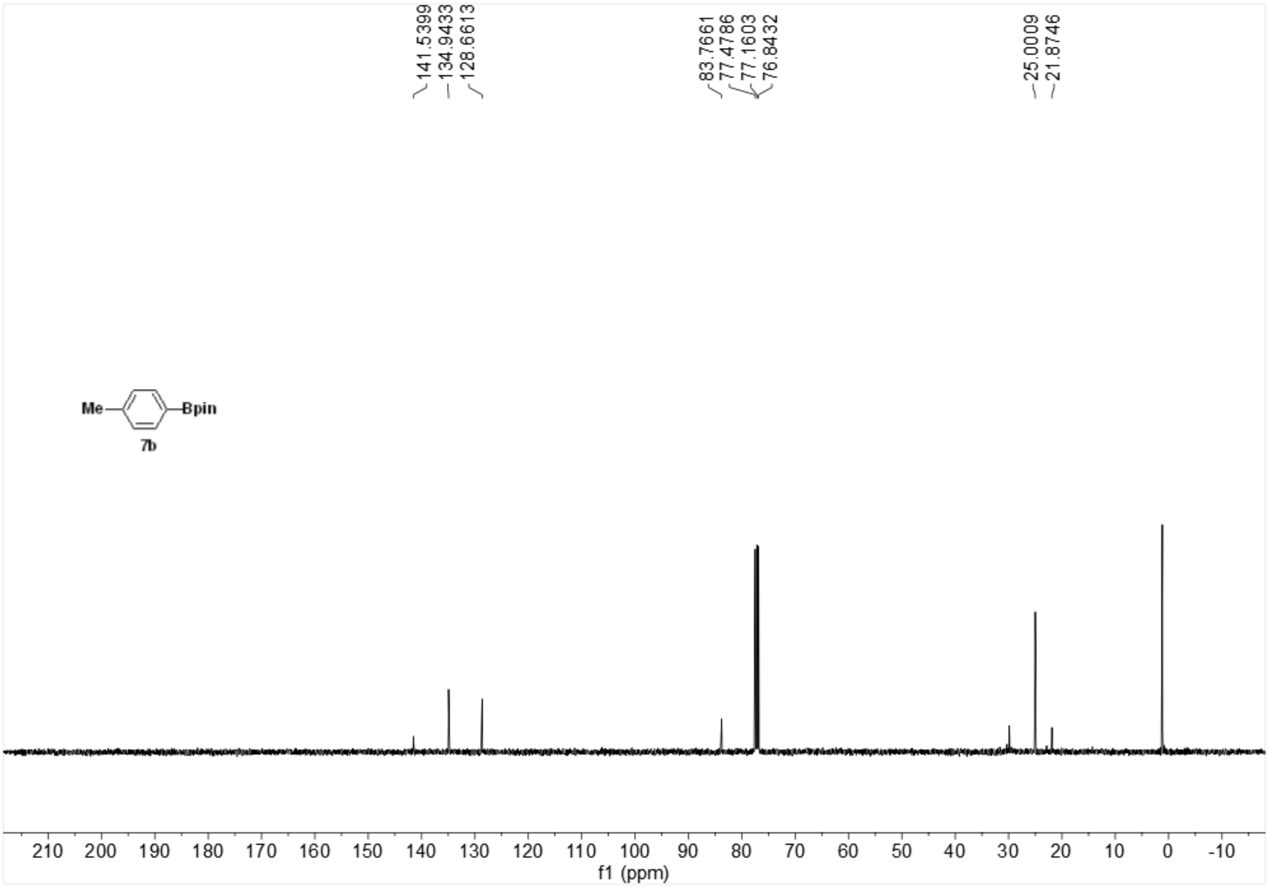

Figure S44. ^{13}C NMR spectrum of compound **7b** ($CDCl_3$, 25 ^{o}C, 101 MHz).

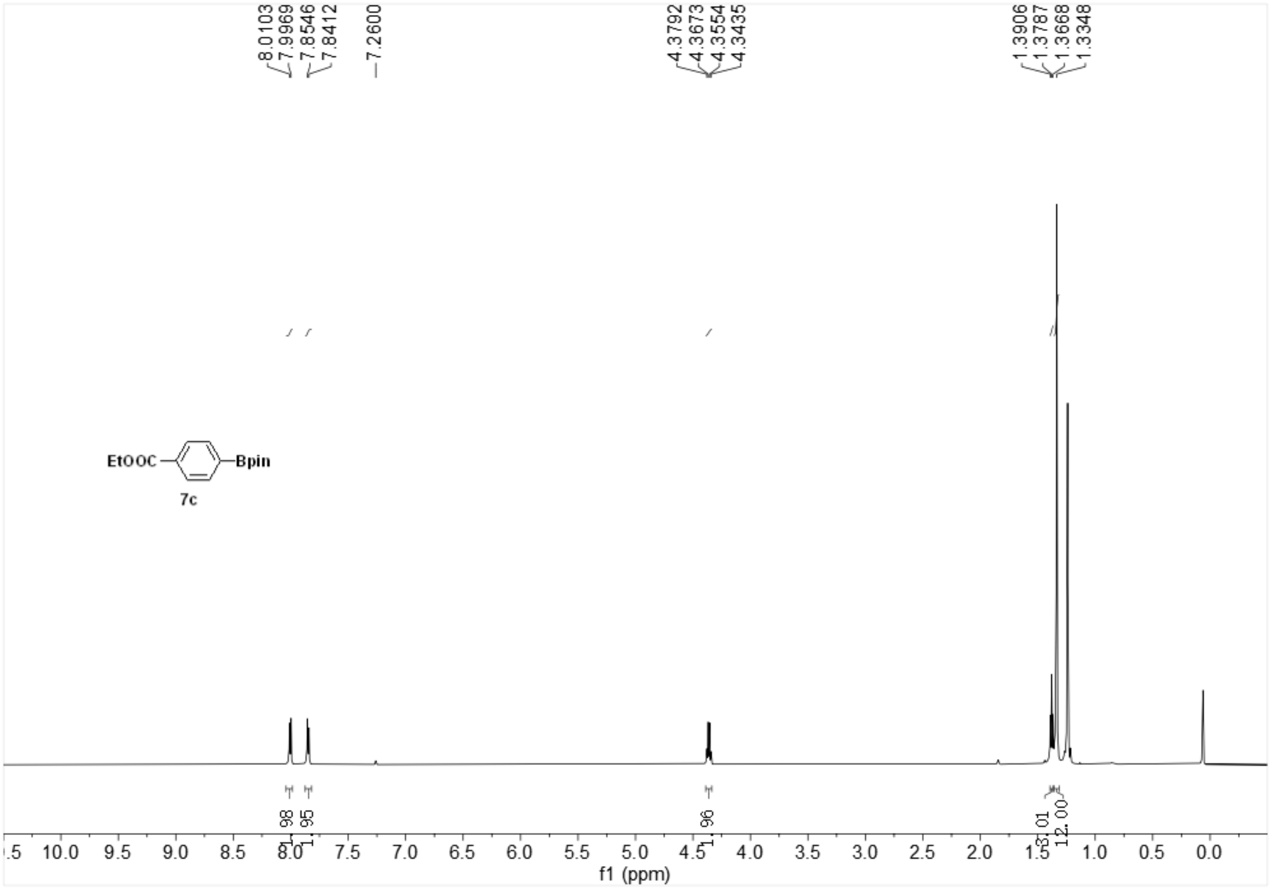

Figure S45. ^{1}H NMR spectrum of compound **7c** ($CDCl_3$, 25 °C, 600 MHz)

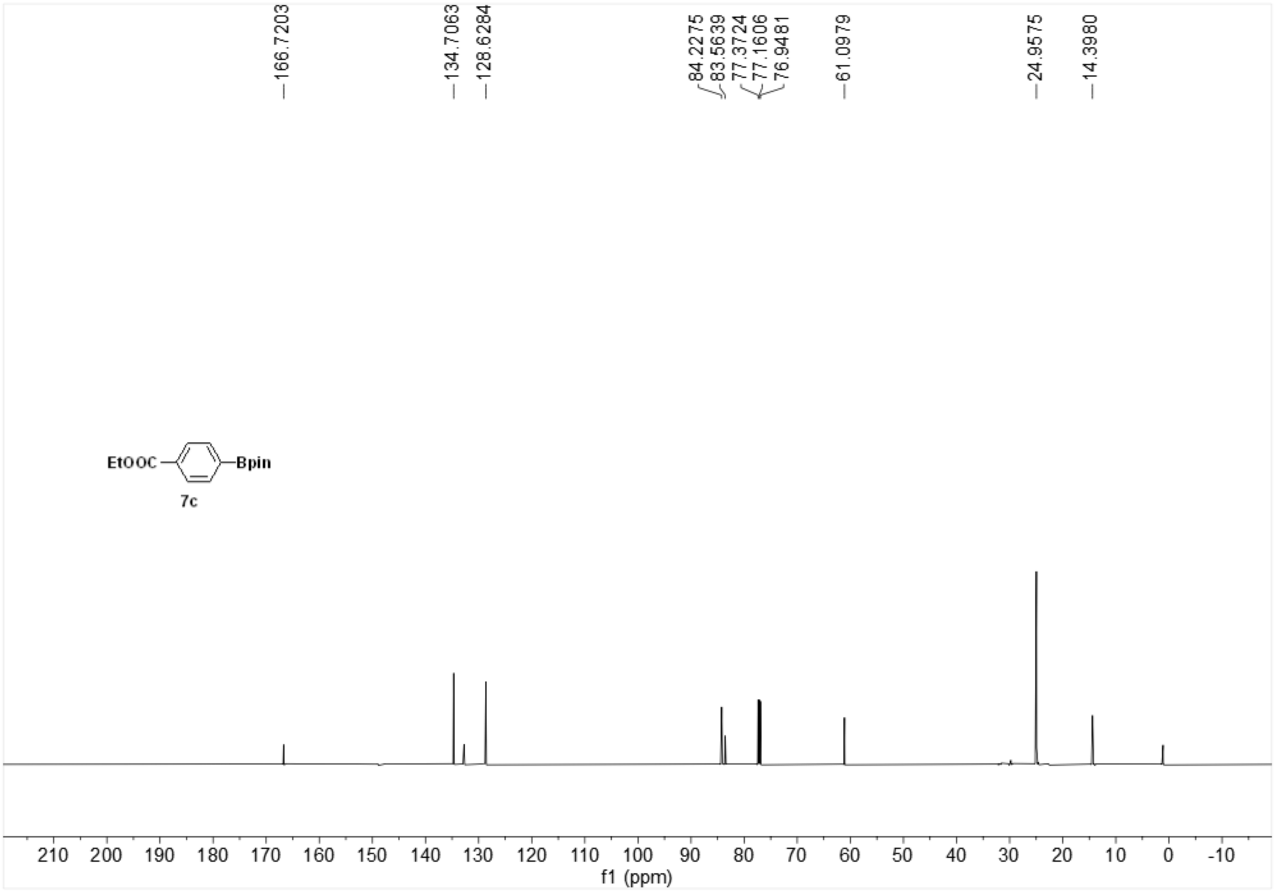

Figure S46. ^{13}C NMR spectrum of compound **7c** ($CDCl_3$, 25 °C, 151 MHz).

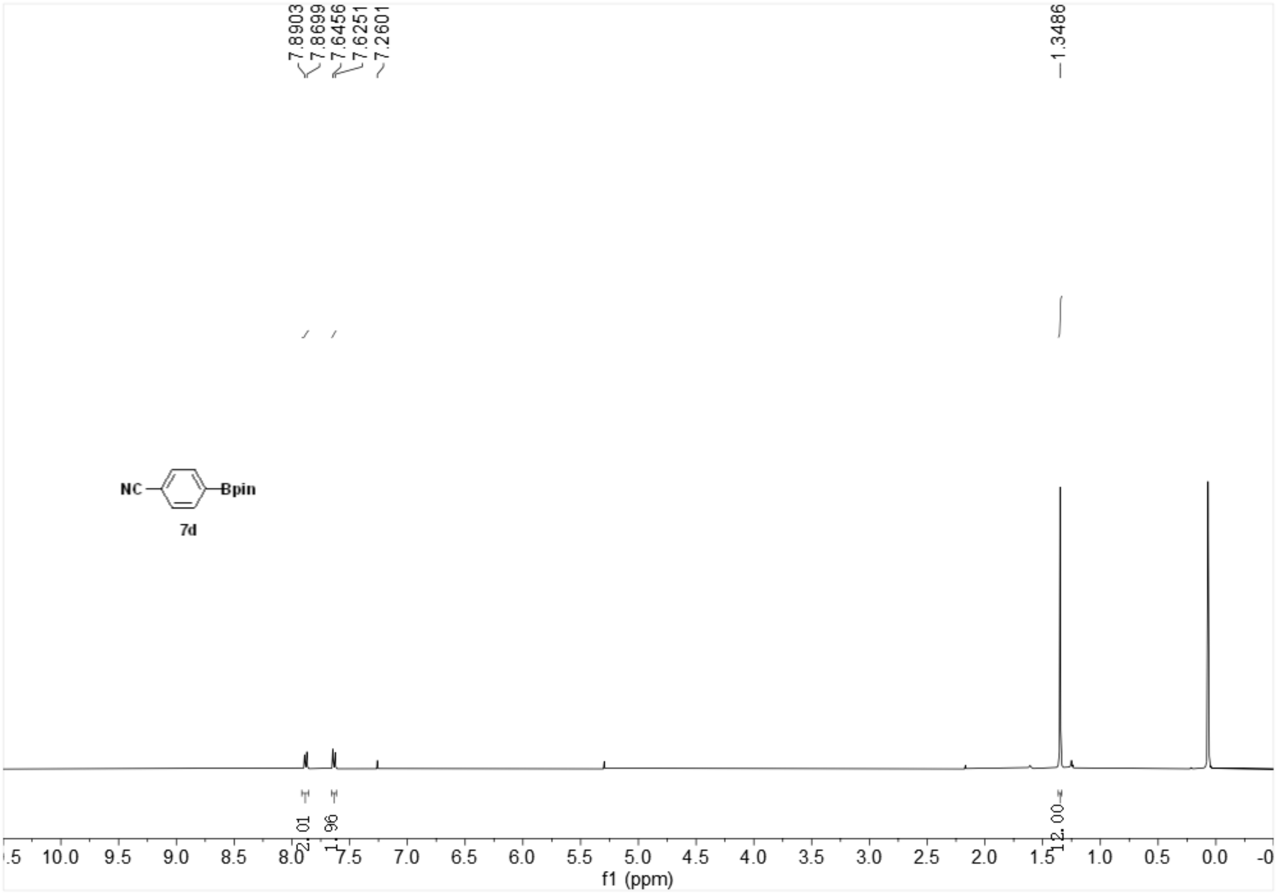

Figure S47. ^{1}H NMR spectrum of compound **7d** ($CDCl_3$, 25 °C, 400 MHz)

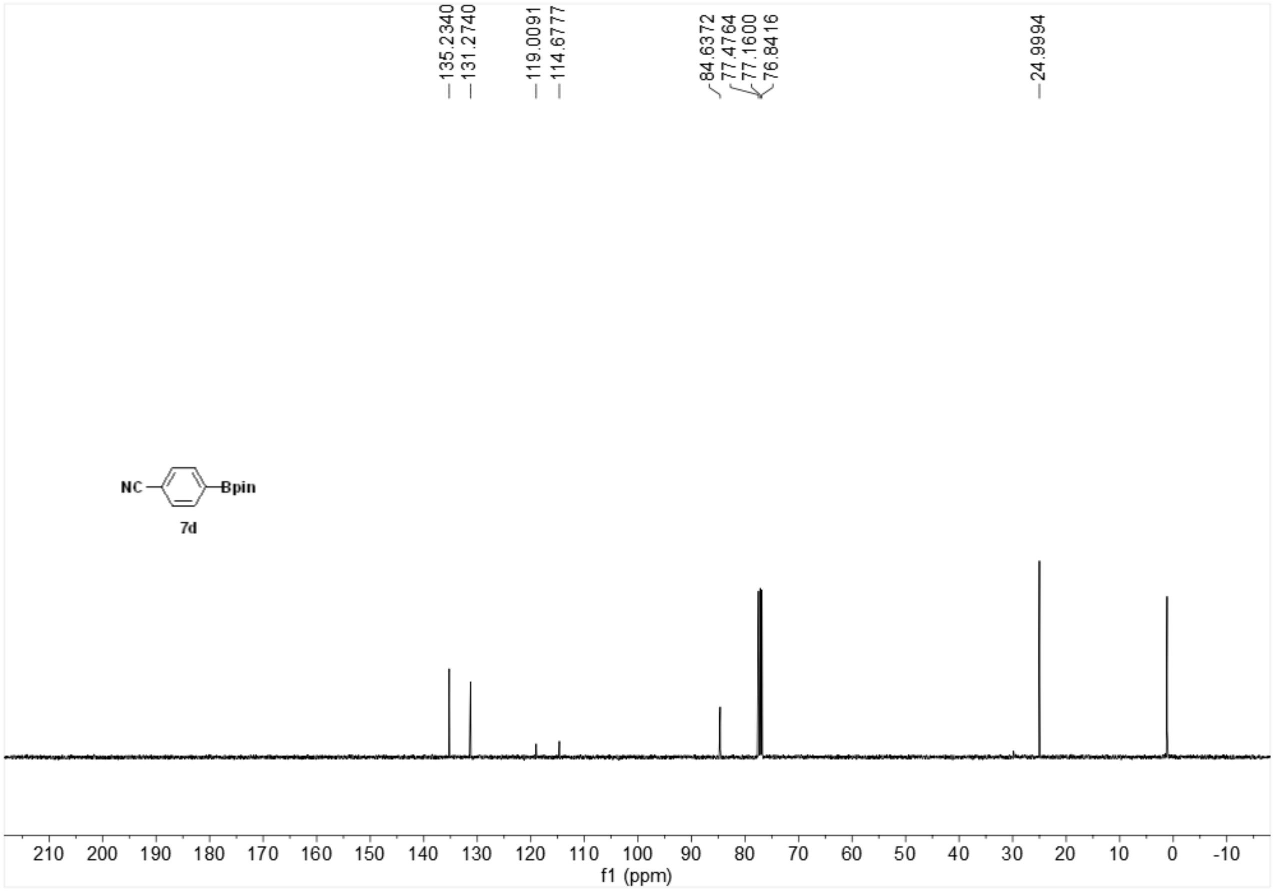

Figure S48. ^{13}C NMR spectrum of compound **7d** ($CDCl_3$, 25 °C, 101 MHz).

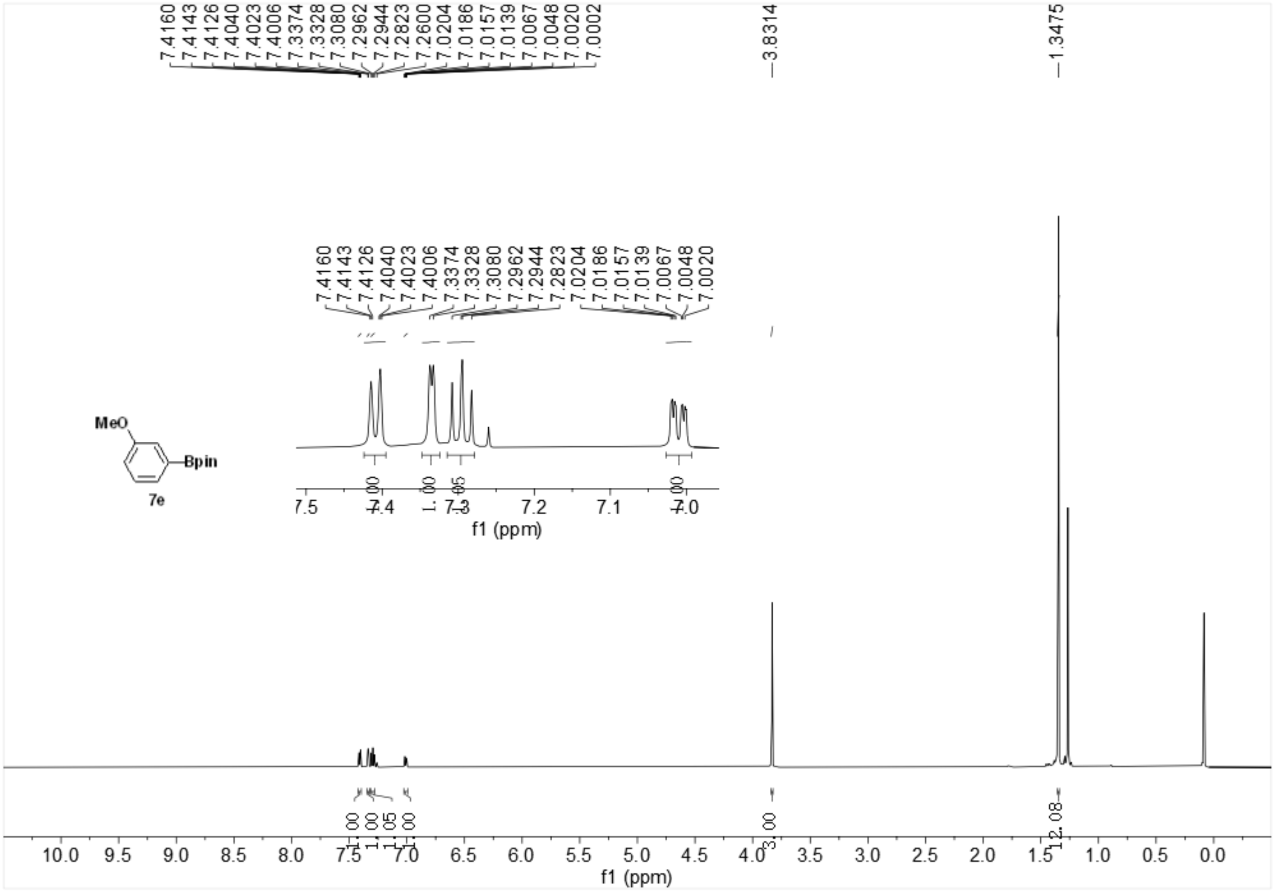

Figure S49. ^{1}H NMR spectrum of compound **7e** ($CDCl_3$, 25 °C, 600 MHz)

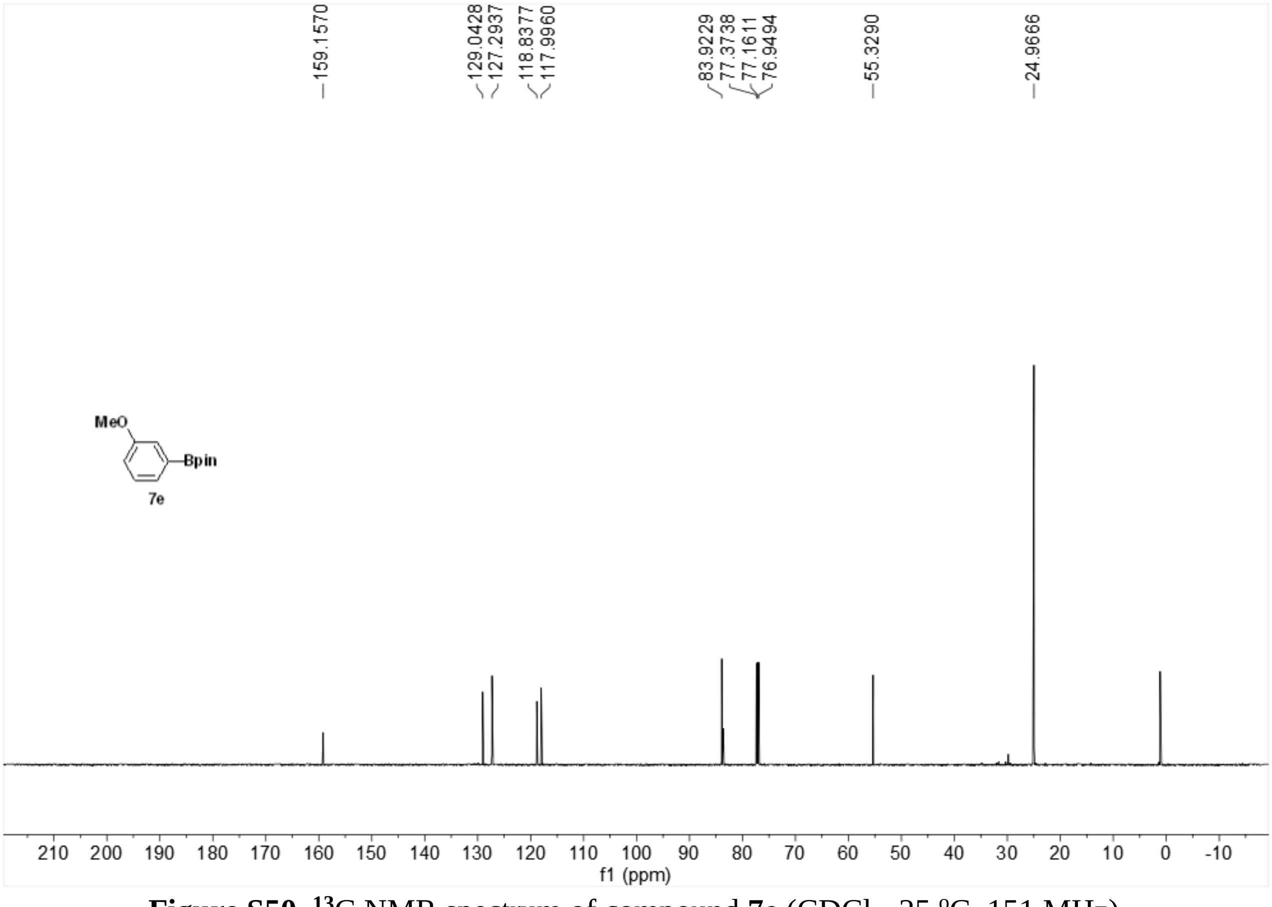

Figure S50. ^{13}C NMR spectrum of compound **7e** ($CDCl_3$, 25 °C, 151 MHz).

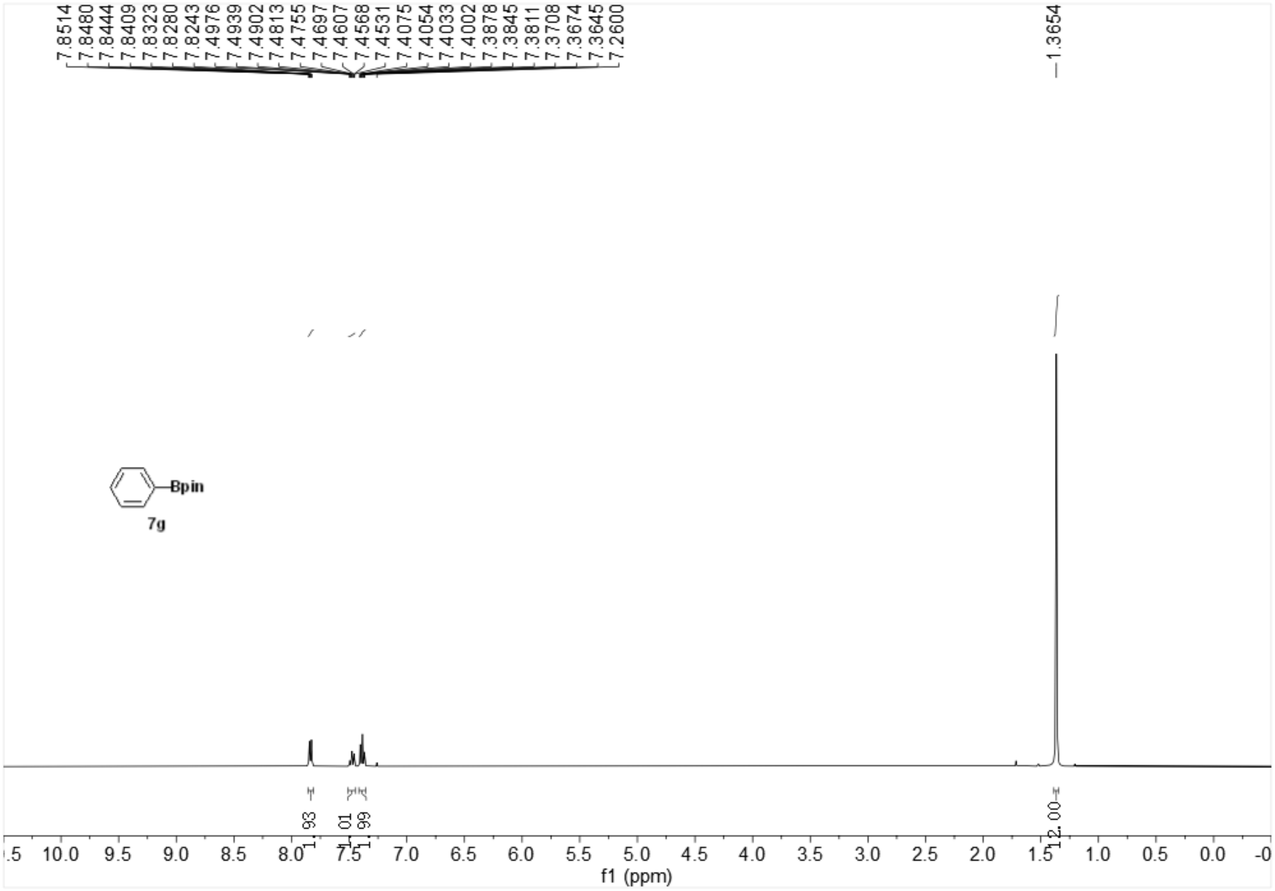

Figure S51. ^{1}H NMR spectrum of compound **7g** ($CDCl_3$, 25 °C, 400 MHz)

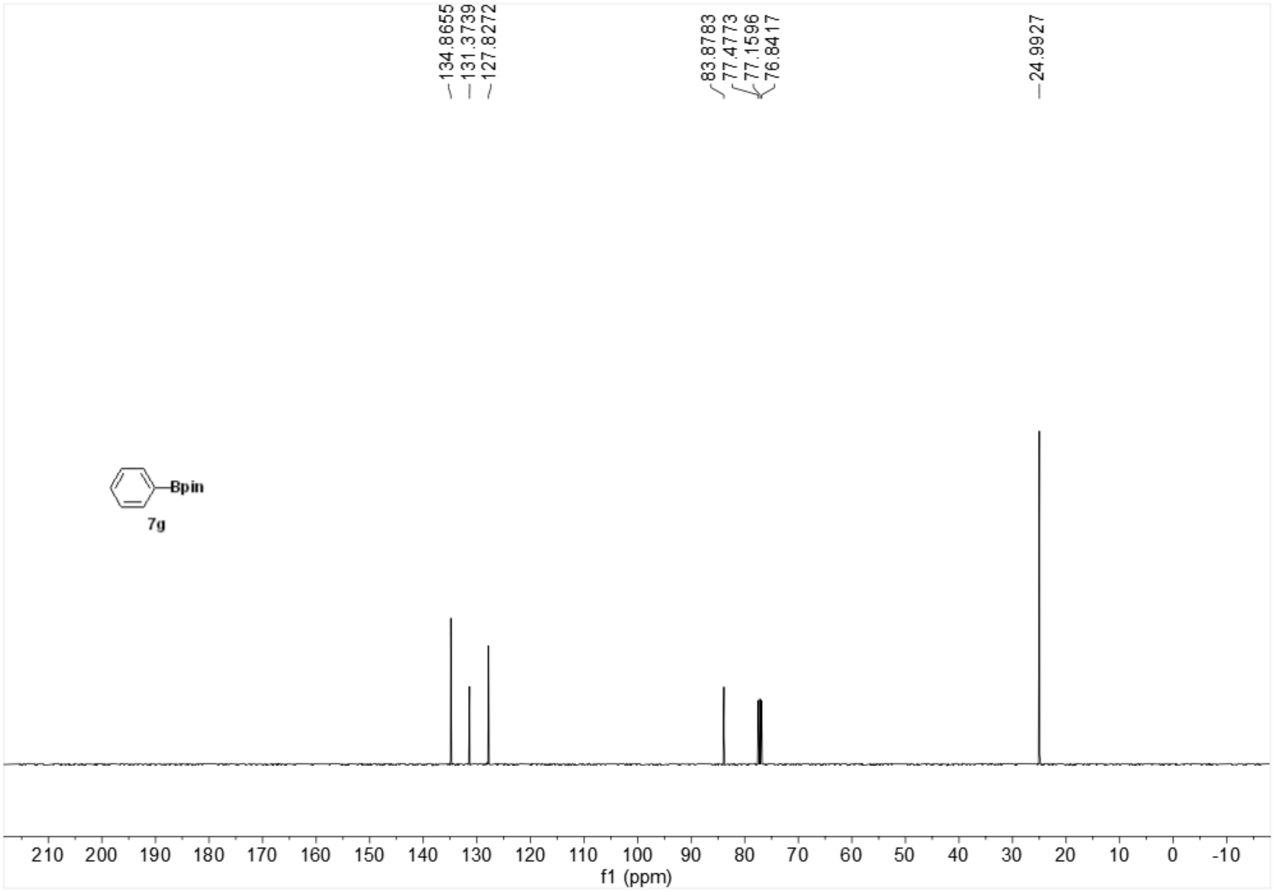

Figure S52. ^{13}C NMR spectrum of compound **7g** ($CDCl_3$, 25 °C, 101 MHz).

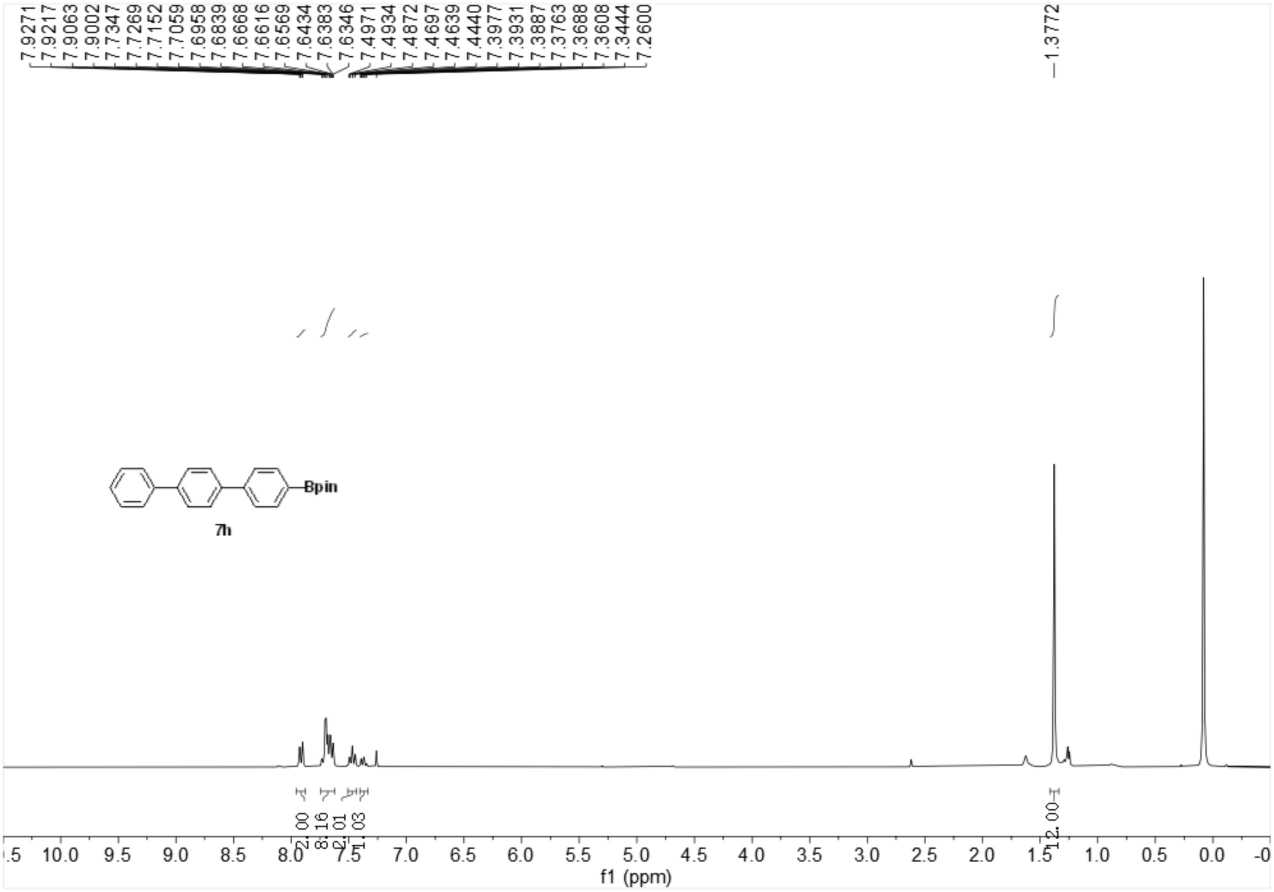

Figure S53. ^{1}H NMR spectrum of compound **7h** ($CDCl_3$, 25 °C, 300 MHz)

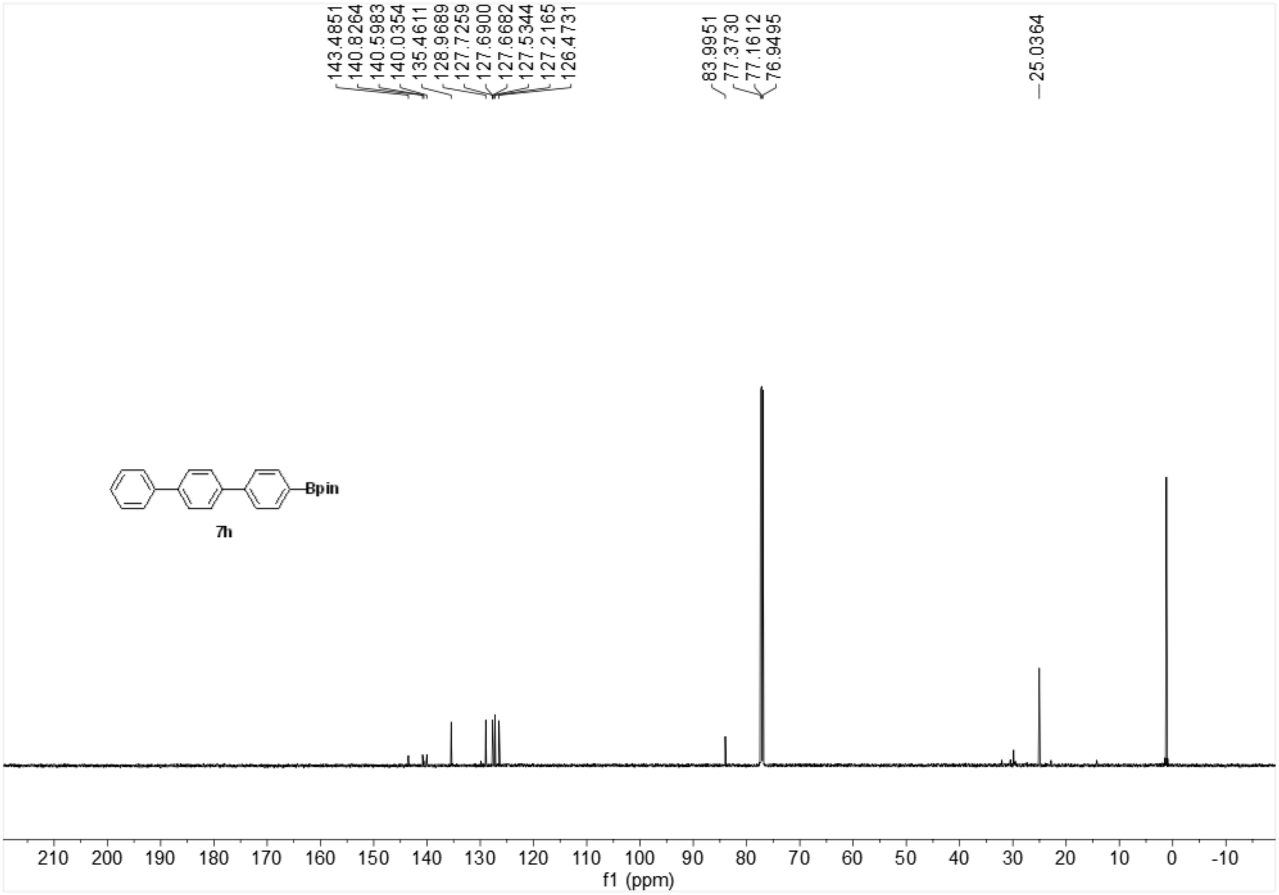

Figure S54. ^{13}C NMR spectrum of compound **7h** ($CDCl_3$, 25 °C, 151 MHz).

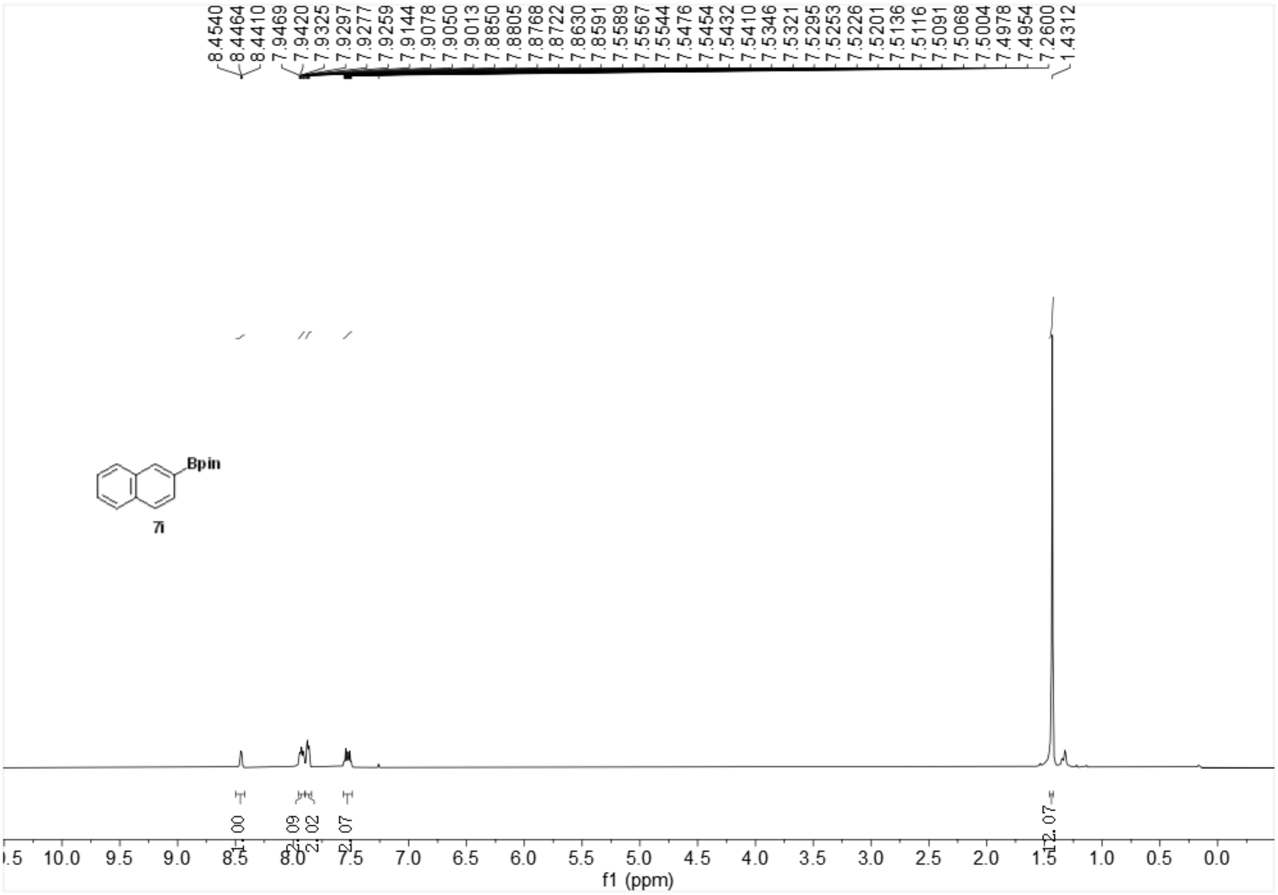

Figure S55. ^{1}H NMR spectrum of compound **7i** ($CDCl_3$, 25 ^{o}C, 600 MHz)

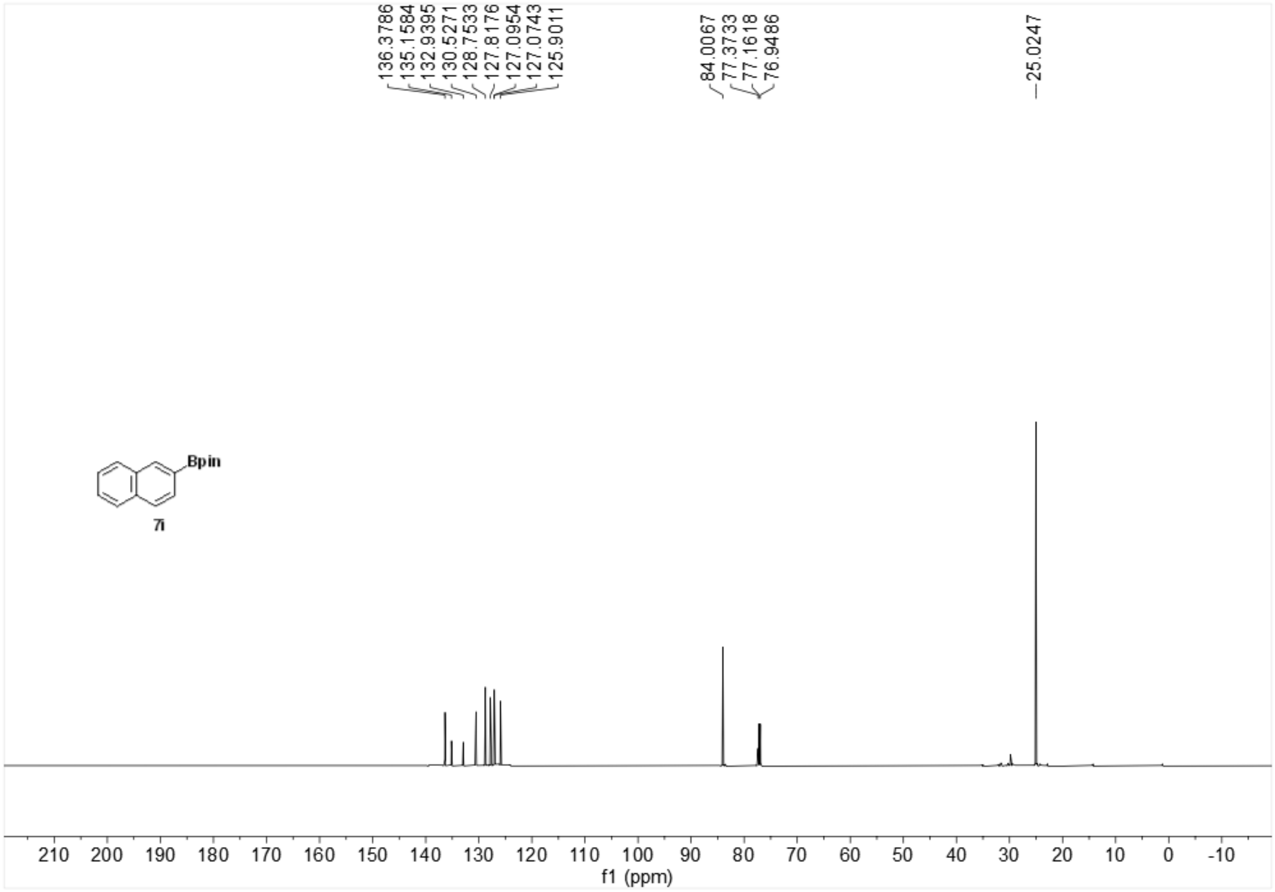

Figure S56. ^{13}C NMR spectrum of compound **7i** ($CDCl_3$, 25 ^{o}C, 151 MHz).

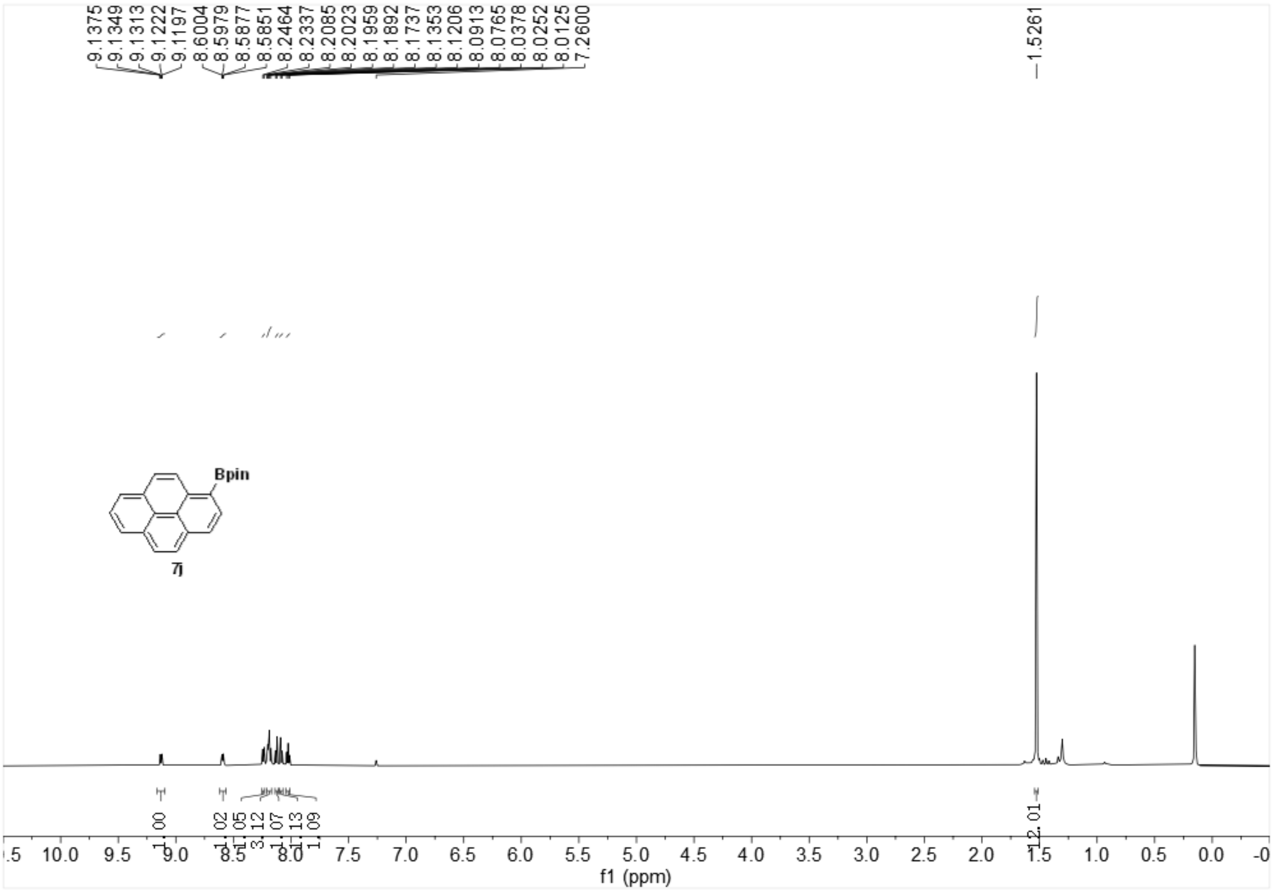

Figure S57. ^{1}H NMR spectrum of compound **7j** ($CDCl_3$, 25 ^{o}C, 600 MHz)

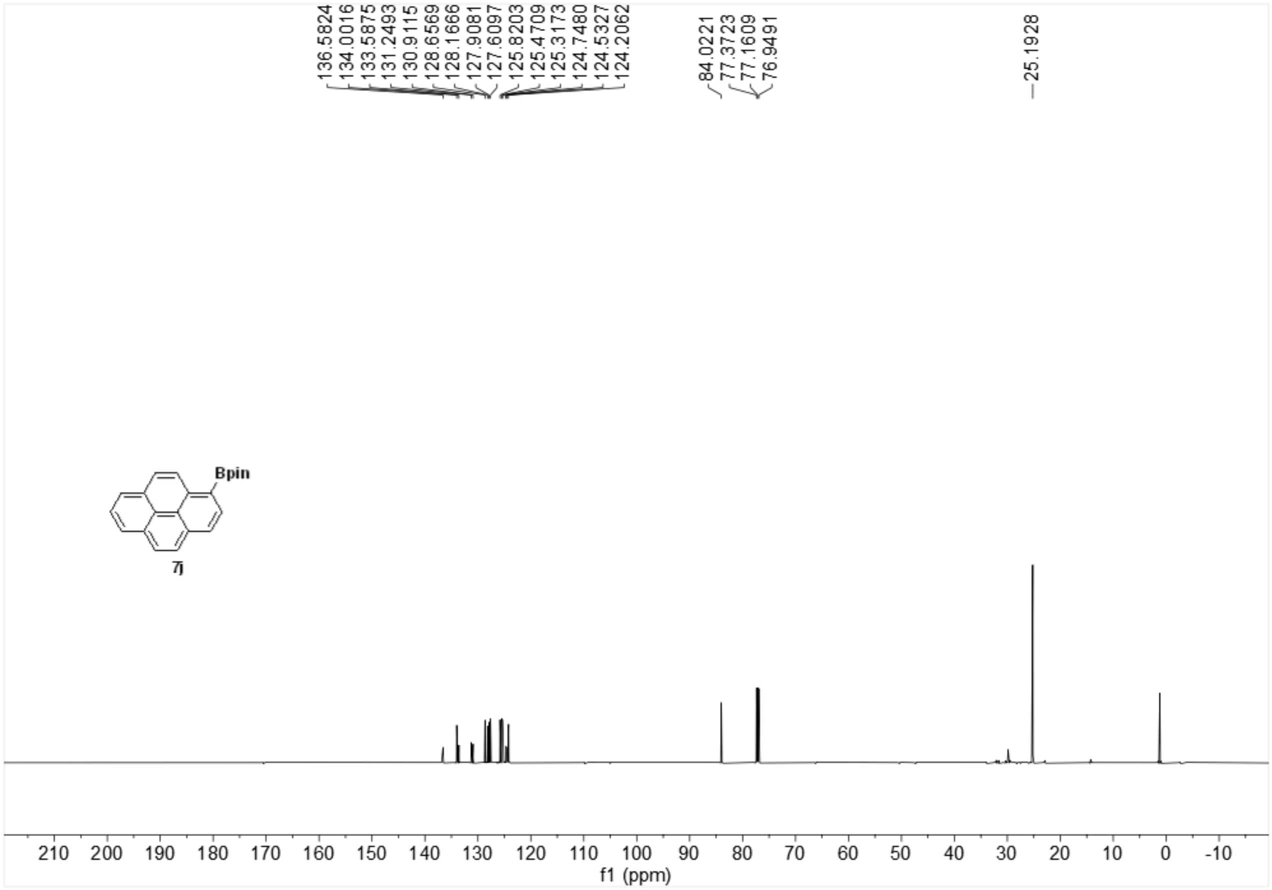

Figure S58. ^{13}C NMR spectrum of compound **7j** ($CDCl_3$, 25 ^{o}C, 151 MHz).

4. References

(1) Neese, F. Software update: The ORCA program system—Version 5.0. *WIREs Comput. Mol. Sci.* **2022**, *12*, e1606.

(2) (a) Grimme, S.; Bannwarth, C.; Shushkov, P. A Robust and Accurate Tight-Binding Quantum Chemical Method for Structures, Vibrational Frequencies, and Noncovalent Interactions of Large Molecular Systems Parametrized for All spd-Block Elements (Z = 1–86). *J. Chem. Theory Comput.* **2017**, *13*, 1989-2009; (b) Bannwarth, C.; Ehlert, S.; Grimme, S. GFN2-xTB—An Accurate and Broadly Parametrized Self-Consistent Tight-Binding Quantum Chemical Method with Multipole Electrostatics and Density-Dependent Dispersion Contributions. *J. Chem. Theory Comput.* **2019**, *15*, 1652-1671; (c) Pracht, P.; Caldeweyher, E.; Ehlert, S.; Grimme, S. A Robust Non-Self-Consistent Tight-Binding Quantum Chemistry Method for large Molecules. *ChemRxiv* **2019**, *preprint*. https://doi.org/10.26434/chemrxiv.8326202.v1

(3) Mardirossian, N.; Head-Gordon, M. ωB97M-V: A Combinatorially Optimized, Range-Separated Hybrid, Meta-GGA Density Functional with VV10 Nonlocal Correlation. *J. Chem. Phys.* **2016**, *144*, 214110.

(4) Weigend, F.; Ahlrichs, R. Balanced basis sets of split valence, triple zeta valence and quadruple zeta valence quality for H to Rn: Design and assessment of accuracy. *Phys. Chem. Chem. Phys.* **2005**, *7*, 3297-3305.

(5) Kruse, H.; Grimme, S. A geometrical correction for the inter- and intra-molecular basis set superposition error in Hartree-Fock and density functional theory calculations for large systems.. *J. Chem. Phys.* **2012**, *136*, 154101.

(6) Marenich, A. V.; Cramer, C. J.; Truhlar, D. G., Universal solvation model based on solute electron density and on a continuum model of the solvent defined by the bulk dielectric constant and atomic surface tensions. *J. Phys. Chem. B.* **2009**, *113*, 6378-6396.

(7) (a)Suzuki, K.; Kobayashi, A.; Kaneko, S.; Takehira, K.; Yoshihara, T.; Ishida, H.; Shiina, Y.; Oishic, S.; Tobita, S. Reevaluation of absolute luminescence quantum yields of standard solutions using a spectrometer with an integrating sphere and a back-thinned CCD detector. *Phys. Chem. Chem. Phys*. **2009**, *11*, 9850-9860; (b) Zhao, M. N.; Pun, S. H.; Gong, Q.; Miao, Q. Carbazole-Fused Polycyclic Aromatics Enabled by Regioselective Scholl Reactions. *Angew. Chem. Int. Ed*. **2021**, *60*, 24124-24130.

(8) Brouwer, A. M. Standards for photoluminescence quantum yield measurements in solution. *Pure Appl. Chem*. **2011**, *83*, 2213-2228.
